# Novel Combination Immunotherapy and Clinical Activity in Patients With HPV-Associated Cancers: A Nonrandomized Clinical Trial

**DOI:** 10.1001/jamaoncol.2024.6998

**Published:** 2025-02-20

**Authors:** Charalampos S. Floudas, Meghali Goswami, Renee N. Donahue, Danielle M. Pastor, Jason M. Redman, Isaac Brownell, Evrim B. Turkbey, Lisa M. Cordes, Seth M. Steinberg, Michell Manu, Deneise C. Francis, Elizabeth Lamping, Jennifer L. Marté, Mary Kackley, Elizabeth Krauss, Manuk Manukyan, Caroline Jochems, Jeffrey Schlom, James L. Gulley, Julius Strauss

**Affiliations:** 1 Center for Immuno-Oncology, Center for Cancer Research, National Cancer Institute, National Institutes of Health, Bethesda, Maryland; 2 Dermatology Branch, National Institute of Arthritis and Musculoskeletal and Skin Diseases, National Institutes of Health, Bethesda, Maryland; 3 Radiology and Imaging Sciences, Center for Cancer Research, National Cancer Institute, National Institutes of Health, Bethesda, Maryland; 4 Biostatistics and Data Management Section, Center for Cancer Research, National Cancer Institute, National Institutes of Health, Bethesda, Maryland; 5 Clinical Research Directorate, Frederick National Laboratory for Cancer Research, Frederick, Maryland; 6 Office of Research Nursing, Center for Cancer Research, National Cancer Institute, National Institutes of Health, Bethesda, Maryland

## Abstract

**IMPORTANCE:**

Patients who experience progression of advanced human papillomavirus (HPV)–associated cancers and who have previously received first-line systemic treatment have a poor prognosis and limited therapeutic options.

**OBJECTIVE:**

To assess the clinical activity of the combination of the HPV type 16 therapeutic vaccine PDS0101, the tumor-targeting interleukin 12 antibody-drug conjugate PDS01ADC, and the bifunctional anti–programmed cell death ligand 1 (PD-L1)/transforming growth factor β (TGF-β) bintrafusp alfa in advanced HPV-associated cancers.

**DESIGN, Setting, and Participants:**

This nonrandomized clinical trial was phase 1/2 and investigator initiated, and was conducted at a single US cancer research center between June 2020 and July 2022. Patients with advanced or metastatic HPV-associated cancers were eligible, including patients who were both immune checkpoint blockade (ICB) naive and ICB resistant. The cutoff date for data analysis was May 13, 2024.

**INTERVENTION:**

Patients received 1 mL of PDS0101 subcutaneously every 4 weeks for 6 doses then every 12 weeks for 2 additional doses, PDS01ADC, 16.8 µg/kg, subcutaneously every 4 weeks or PDS01ADC, 8 µg/kg, subcutaneously every 2 weeks, and bintrafusp alfa, 1200 mg, intravenously every 2 weeks.

**MAIN OUTCOMES AND MEASURES:**

Objective response rate (ORR) by Response Evaluation Criteria in Solid Tumors version 1.1 in ICB-naive patients.

**RESULTS:**

Of the 50 eligible patients, 26 (52%) were men and the median age was 56 years (range, 28-80 years). The median (IQR) follow-up was 37.7 (30.6-42.0) months. Fourteen patients (28%) were ICB naive, with an ORR of 35.7% (95% CI, 12.8%-64.9%), and median overall survival (OS) 42.4 months (95% CI, 8.3 months-not estimable); in ICB-resistant patients, the ORR was 16.7% (6 of 36 patients; 95% CI, 6.4%-32.8%) and median OS was 15.8 months (95% CI, 9.0-21.3 months). Among patients with HPV-16–positive tumors (37 patients [74%]), in the ICB-naive group (8 patients [21.6%]) the ORR was 62.5% (95% CI, 24.5%-91.5%) and a median OS measure was not reached. Grade 3 and 4 treatment-related adverse events occurred in 26 of 50 patients (52%). There were no treatment-related deaths.

**CONCLUSIONS AND RELEVANCE:**

In this trial, the combination of PDS0101, PDS01ADC, and bintrafusp alfa showed an acceptable safety profile and promising antitumor activity and improved OS in patients with HPV-16–positive cancers, in both ICB-naive and ICB-resistant patients, warranting further evaluation of the combination of PDS0101 and PDS01ADC with simultaneous PD-L1/TGF-β inhibition in these populations.

**Trial Registration:**

ClinicalTrials.gov Identifier: NCT04287868

## Key Points

**Question:**

What is the clinical activity of combining the tumor-targeting interleukin 12 antibody-drug conjugate PDS01ADC, the bifunctional anti–programmed cell death ligand 1 (PD-L1)/transforming growth factor β (TGF-β) bintrafusp alfa, and the human papillomavirus (HPV) type 16 therapeutic vaccine PDS0101 in adult patients with advanced HPV-associated cancers?

**Findings:**

In this single-center, phase 1/2 nonrandomized trial of 50 patients with previously treated advanced HPV-associated cancers, a response rate of 36.7% was achieved in immune checkpoint blockade–naive patients, increasing to 62.5% in patients with HPV-16–positive tumors.

**Meaning:**

This trial identified a population of patients with HPV-associated cancer with promising antitumor responses and improved survival, supporting further evaluation of PDS0101, PDS01ADC, and simultaneous PD-L1/TGF-β inhibition in patients with HPV-16–positive tumors.

## Teaser

This nonrandomized clinical trial examines the clinical activity and adverse events of a novel combination treatment for advanced human papillomavirus–associated cancers.

## Introduction

More than 630 000 cases of human papillomavirus (HPV)–associated cancers (eg, oropharyngeal, cervical, anal, vulvovaginal, penile) occur annually worldwide.^1^ The US Food and Drug Administration (FDA) approved nivolumab and pembrolizumab (programmed cell death 1 protein [PD-1] immune checkpoint blockade [ICB]) in platinum-experienced recurrent or metastatic head and neck squamous cell carcinoma (HNSCC), and pembrolizumab in platinum-experienced cervical carcinoma based on objective response rates (ORR) ranging from 11% to 24%.^2,3,4,5,6,7,8,9,10^ However, most patients with advanced HPV-associated cancer will progress while taking ICB and lack an effective standard-of-care treatment.

In advanced cancers, transforming growth factor β (TGF-β) suppresses antitumor immune responses and drives tumor angiogenesis and epithelial-to-mesenchymal transition, contributing to resistance to anticancer treatment.^11,12,13^ HPV-associated cancers overexpress TGF-β receptor 1 and are associated with the TGF-β pathway.^14^ Bintrafusp alfa (BA) is a bifunctional fusion protein sequestering TGF-β and blocking programmed cell death 1 ligand 1 (PD-L1) in the tumor microenvironment (TME). In phase 1 and 2 trials, BA induced responses in 30.5% of patients with ICB-naive HPV-associated cancers and in 10% of ICB-resistant HPV-associated cancers; benefit was likelier in patients developing HPV-specific T-cell immune responses.^15,16^ HPV-specific T-cell immune responses may be induced by HPV-targeting therapeutic cancer vaccines leading to improved clinical activity of BA. PDS0101 is a micellar multipeptide therapeutic vaccine targeting the E6 and E7 oncoproteins of HPV type 16, the genotype causing most HPV-associated cancers. The peptides are coadministered with the cationic lipid nanoparticle R-DOTAP (Versamune), which upregulates type 1 interferons and promotes antigen cross-presentation. In a phase 1 trial in patients with cervical intraepithelial neoplasia, PDS0101 induced HPV-specific CD4^+^ and CD8^+^ T-cell immune responses and was well tolerated, with mild injection-site reactions and minimal systemic adverse events; clinical responses were also observed in participants with non–HPV-16 types.^17^ PDS01ADC (previously designated M9241, NHS-IL12, and PDS0301) is a tumor-targeting IL-12 antibody-drug conjugate, binding to histones on free DNA fragments in areas of tumor necrosis.^18,19,20^ In a phase 1 trial in patients with advanced solid tumors, PDS01ADC was well tolerated and associated with increased TME T-cell infiltration.^21,22^ These data suggest that a combination of PDS0101, PDS01ADC, and BA might improve anticancer activity over the respective single agents or doublets: preclinical evaluation of PDS0101, PDS01ADC, and BA as single agents, doublets, and triplet in the TC-1 HPV-16–positive tumor model, revealed the greatest HPV-specific immune responses, T-cell tumor infiltration, and tumor reduction with the triplet.^23^ We conducted a phase 1/2 nonrandomized clinical trial to examine the clinical activity of the combination of PDS0101, PDS01ADC, and BA in patients with advanced HPV-associated cancers. Further details are available in eAppendix 1 in Supplement 2.

## Methods

This phase 1/2 nonrandomized clinical trial was conducted at the Center for Cancer Research of the National Cancer Institute (protocol in Supplement 1; eMethods in Supplement 2). The trial was approved by the National Institutes of Health (NIH) Institutional Review Board (IRB) in February 2020 and conducted in accordance with the Declaration of Helsinki^24^ and Good Clinical Practice standards. All participants provided written informed consent. Additional follow-up of patients was conducted on long-term follow-up and data collection protocols (NIH IRB approved, NCT00451022 and NCT00923065, respectively). The Transparent Reporting of Evaluations With Nonrandomized Designs (TREND) reporting guideline was followed. Eligible patients were 18 years or older, had histologically confirmed locally advanced or metastatic HPV-associated cancers or HPV-positive cancers, measurable lesions according to Response Evaluation Criteria in Solid Tumors (RECIST) version 1.1, Eastern Cooperative Oncology Group Performance Status of 0, 1, or 2, and adequate organ function as defined in the protocol, with progression after 1 or more lines of systemic therapy, unless the patient was ineligible for or declined standard-of-care treatment. Prior ICB was not allowed but the protocol was modified first to allow prior ICB and subsequently to require prior ICB following changes in standard-of-care first-line treatment of recurrent and/or metastatic HNSCC and cervical cancer. HPV status was not required for enrollment.

Patients received 1200 mg of intravenous BA (reduced starting doses of 600 or 300 mg allowed) every 2 weeks; 16.8 µg/kg of subcutaneous PDS01ADC every 4 weeks or 8 µg/kg every 2 weeks; and 1 mL (3 mg R-DOTAP, 2.7 mg total peptide) of PDS0101 subcutaneously (split into two 0.5-mL injections) every 4 weeks for 6 doses, then every 12 weeks for 2 additional doses. Interruptions (any drug) and dose reductions of PDS01ADC (to 12 or 8 µg/kg every 4 weeks) were allowed for adverse events management. Protocol modifications allowed BA dose reductions to 600 mg or 300 mg (including starting dose); PDS0101 discontinuation in patients with non–HPV-16 tumors; and PDS01ADC starting dose of 16.8 µg/kg every 4 weeks for up to 4 doses, followed by 8 µg/kg every 4 weeks, allowing dose reductions to 8, 6, or 4 µg/kg. Treatment continued until disease progression, unacceptable adverse events, consent withdrawal, or completion of 1-year treatment (PDS01ADC and BA could continue beyond 1 year).

Primary end point was ORR (proportion of confirmed complete or partial responses by RECIST 1.1) in patients with advanced ICB-naive HPV-associated cancers. Secondary end points included assessment of safety, progression-free survival (PFS; defined as time from treatment initiation to disease progression or death from any cause), and overall survival (OS; defined as time from treatment initiation to death from any cause) in patients with HPV-associated cancers. Exploratory analyses included responses by Immune-Related RECIST^25^ in patients with ICB-naive HPV-associated cancers, ORR in patients with ICB-resistant HPV-associated cancers, ORR by HPV serotype and by dose level of PDS01ADC or BA, and circulating HPV-16–specific T-cell responses as previously described.^26^

Enrollment of up to 20 evaluable ICB-naive patients was planned. Safety was assessed in all patients who received at least 1 dose of any study drug. Response was assessed in all patients who received at least 1 dose of any drug and had disease re-evaluation or exhibited objective disease progression. Clopper-Pearson 95% CIs of ORR were calculated. OS and PFS were estimated using the Kaplan-Meier method. Statistical analyses were performed using R version 4.3.3 (R Core Team), Prism version 10.4.0 (GraphPad), and SAS version 9.4 (SAS Institute).

## Results

Between June 9, 2020, and July 26, 2022, 51 patients were screened; 50 were enrolled and received treatment (median age, 56 years [range, 28-80]; 24 female [48%]) (Figure). Baseline characteristics are detailed in eTable 1 in Supplement 2. Tumor types included oropharyngeal (21 [42%]), cervical (14 [28%]), anal or rectal (10 [20%]), vulvar or vaginal (3 [6%]), nasopharyngeal (1 [2%]), and penile (1 [2%]). All patients had prior platinum-based chemotherapy as adjuvant treatment or for recurrent and/or metastatic disease; 45 of 50 (90%) had 2 or more lines of prior anticancer therapy and 36 of 50 (72%) had prior ICB. Forty-eight of 50 patients (96%) had confirmed HPV-positive tumors: 37 (74%) had HPV-16–positive tumors, 11 (22%) had non–HPV-16 genotypes (eg, HPV-18, HPV-45), 1 (2%) had an HPV-negative tumor, and 1 (2%) had a tumor with an unknown HPV status. Twenty-one of 50 patients (42%) received a full starting dose of PDS01ADC (16.8 µg/kg every 4 weeks) and BA (1200 mg every 2 weeks). Sixteen of 50 patients (32%) received full doses of BA with a lower dose of PDS01ADC (8 µg/kg every 4 weeks for 8 patients and 8 µg/kg every 2 weeks for 8 patients). Seven of 50 patients (14%) received a full dose of PDS01ADC with a lower dose of BA (300 mg every 2 weeks). Six of 50 patients (12%) received lower starting doses of both medications.

**Figure.**
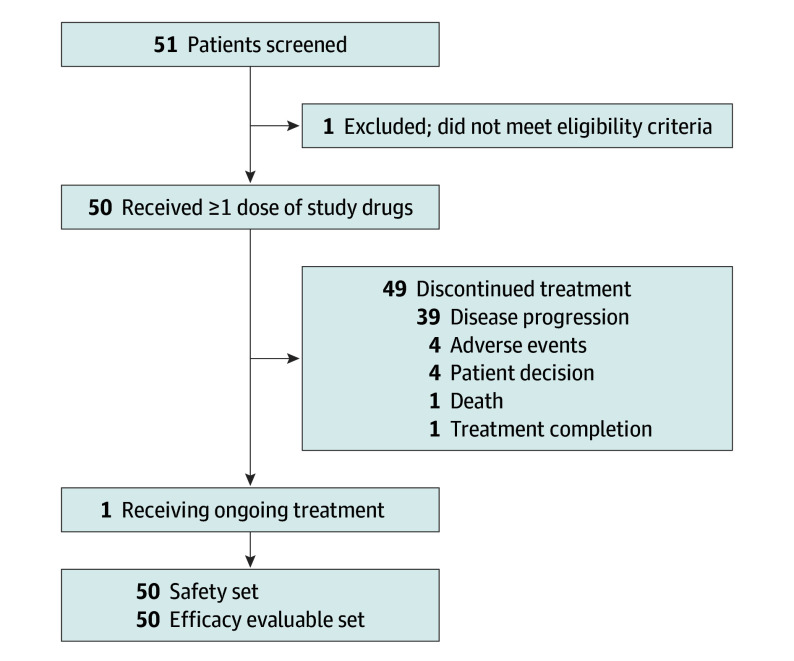
Patient Flowchart

At data cutoff date of May 13, 2024, median (IQR) follow-up by the reverse Kaplan-Meier method was 37.7 (30.6-42.0) months. Median (IQR) duration of treatment for the 49 who completed treatment was 2.3 (1.4-4.6) months; 1 patient was receiving treatment after 3.4 years.

Treatment-related adverse events (TRAEs) of any grade occurred in all patients, and grade 3 and 4 TRAEs occurred in 26 patients (52%) (Table 1; eTable 2 in Supplement 2). The most frequent TRAEs of any grade attributed to the PDS0101 vaccine were of grade 1 and 2, reported in 41 patients (82%) overall, most commonly injection site reactions (36 patients [72%]) and flu-like symptoms (15 patients [30%]). Grade 3 or higher TRAEs observed in 5% or more of patients included anemia (13 patients [26%]), hematuria (5 patients [10%]), lymphocytopenia (4 patients [8%]), alanine aminotransferase elevation (3 patients [6%]), and aspartate aminotransferase elevation (3 patients [6%]) (Table 1; eTable 2 in Supplement 2). Grade 3 and 4 TRAEs (eTable 2 in Supplement 2) were more common with full doses of both PDS01ADC and BA (eTable 3 and eResults in Supplement 2). Serious TRAEs occurred in 13 patients (26%) (eTable 2 in Supplement 2). TRAEs leading to discontinuation of treatment occurred in 12 patients (24%) and included liver function test increase (3 [6%]), bleeding (3 [6%]; specifically gastric bleeding, anal bleeding, hematuria), anemia (1 [2%]), myocarditis (1 [2%]), myositis (1 [2%]), pneumonitis (1 [2%]), colitis (1 [2%]), and gastroparesis (1 [2%]). There were no treatment-related deaths. The frequency of all mucosal bleeding adverse events attributed to BA (adverse event of special interest) was 70% (any grade) and was grade 3 in 18% (no higher grade) (eAppendix 2 in Supplement 2).

**Table 1.** Treatment-Related Adverse Events (TRAEs)

Adverse event	Patients, No (%) (N = 50)	
	Any grade^a^	Grade ≥3^b^
TRAE	50 (100)	26 (52)
Injection-site reactions	36 (72)	0
Flu-like symptoms	32 (64)	0
Anemia	26 (52)	13 (26)
Gingival bleeding	17 (34)	0
Fatigue	16 (32)	0
Hematuria	12 (24)	5 (10)
Epistaxis	12 (24)	0
Aspartate aminotransferase increased	12 (24)	3 (6)
Rash, maculopapular	11 (22)	0
Pruritus	11 (22)	0
Mucositis, oral	11 (22)	0
Lymphocyte count, decreased	10 (20)	4 (8)
Nausea	9 (18)	0
Headache	8 (16)	0
Alanine aminotransferase increased	7 (14)	3 (6)
Vomiting	7 (14)	0
Alkaline phosphatase increased	6 (12)	0
Mucosal bleeding^c^	6 (12)	2 (4)
Keratoacanthoma	5 (10)	0
White blood cell decreased	5 (10)	0
Rash, acneiform	5 (10)	0
Fever	5 (10)	0
Special interest events^d^		
Mucosal bleeding	35 (70)	9 (18)

^a^ Events of any grade reported in 10% or more of patients.

^b^ Events of grade 3 or greater reported in 2 or more patients.

^c^ Any mucosal bleeding not otherwise specified.

^d^ Composite category: all mucosal bleeding adverse events related to bintrafusp alfa.

The ORR was 35.7% (5 of 14 patients; 95% CI, 12.8%–64.9%) in the primary end point population of ICB-naive patients, with 2 additional responses by Immune-Related RECIST; 16.7% (6 of 36 patients; 95% CI, 6.4%-32.8%) in ICB-resistant patients, and 22% (11 of 50; 95% CI, 11.5%-36.0%) in the overall trial population (Table 2; eTable 4 in Supplement 2). Durable responses were seen in all tumor types (eFigure 1 in Supplement 2). Median duration of response was 20.2 months (95% CI, 5.2 months-not estimable) for the ICB-naive population and 6.5 months (95% CI, 3.7 months-not estimable) for the ICB-resistant population. For ICB-naive patients, median OS was 42.4 months (95% CI, 8.3 months-not estimable), and median PFS was 2.9 months (95% CI, 1.8–13.8 months) (eFigure 2 in Supplement 2). For ICB-resistant patients, median OS was 15.8 months (95% CI, 9.0-21.3 months), and median PFS was 1.9 months (95% CI, 1.8-2.8 months). In the 37 HPV-16–positive patients, ORR was 29.7% (11 of 37; 95% CI, 15.9%-47.0%). In HPV-16–positive ICB-naive patients, ORR was 62.5% (5 of 8 patients; 95% CI, 24.5%-91.5%), with an additional patient having a response by Immune-Related RECIST (75%; 95% CI, 34.9%-96.8%), median OS was not reached, and median PFS was 11.3 months (95% CI, 1.6-30.8 months). In HPV-16–positive ICB-resistant patients, ORR was 20.7% (6 of 29 patients; 95% CI, 8.0%-39.7%), median OS was 17.0 months (95% CI, 10.4–22.8 months), and median PFS was 2.4 months (95% CI, 1.8-5.0 months). eAppendix 2 in Supplement 2 reports subgroup results.

**Table 2.** Tumor Response in Evaluable Patients

Response^a^	All patients (N = 50)	ICBN (n = 14)	HPV-16–positive (n = 37)	HPV-16–positive ICBN (n = 8)
Objective response rate, No. (%) [95% CI]	11 (22.0%) [11.5%-36.0%]	5 (35.7%) [12.7%-64.9%]	11 (29.7%) [15.9-47.0]	5 (62.5%) [24.5%-91.5%]
Complete response, No.	4	2	4	2^b^
Partial response, No.	7	3	7	3
Stable disease, No.	6	1	6	1
Progression of disease, No.	33	8	20	2

Abbreviations: HPV, human papillomavirus; ICBN, immune checkpoint blockade naive; RECIST, Response Evaluation Criteria in Solid Tumors.

^a^ Responses assessed by study radiologist according to the RECIST version 1.1.

^b^ Confirmed responses (1 complete response considered by investigator as confirmed since lesion emerging in prior lesion site not growing in follow-up imaging and anoscopy surveillance over 2 years). Objective response rate includes the complete responses plus partial responses.

Evaluation of HPV-16–specific T cells in patients with HPV-16–positive disease with available peripheral blood mononuclear cells before and during treatment revealed patients with complete response, partial response, or stable disease were likelier to have increased multifunctional T-cell responses while taking treatment (1113 patients [85%]) compared with patients with progressive disease (2 of 7 patients [29%]) (*P* = .02) (eFigures 3 and 4, eAppendix 2 in Supplement 2).

## Discussion

Based on preclinical data,^18,23^ we conducted a phase 1/2 nonrandomized clinical trial of PDS0101, PDS01ADC, and BA in patients with advanced HPV-associated cancers. In this study, the combination had an acceptable toxic effect profile, with improved clinical activity compared with standard ICB, and notable response rates and survival outcomes in patients with ICB-naive or ICB-resistant HPV-16–positive tumors.

In our study, 5 of 8 patients with advanced platinum-experienced ICB-naive HPV-16–positive disease had objective responses by RECIST version 1.1, with median OS not reached and 24-month survival rates of 75% noted. By comparison, in patients with advanced platinum-experienced ICB-naive HPV-related cancers, standard PD-1 blockade produced response rates ranging from 11% to 24%,^2,3,4,5,6,7,8,9,10^ with median OS ranging from 7 to 12 months (eTable 4 in Supplement 2).^2,3,5,6,7,8^ Among patients with ICB-resistant HPV-16–positive disease, ORR was 20.7% and median OS was 17.0 months; median OS for similar patients with platinum-experienced ICB-resistant disease is 3 to 4 months.^15^ In a patient population with ICB-resistant disease, where the majority have no clear effective standard of care, a response rate exceeding 20% with the combination of PDS0101, PDS01ADC, and BA is notable, as is the improved survival rate. In addition, responses were durable and observed in all HPV-associated tumor types evaluated.

The combination had an acceptable safety profile, with grade 3 and 4 TRAEs occurring in 52% of all patients, and no treatment-related deaths. In comparison, the combination of ipilimumab and nivolumab produces grade 3 or 4 toxic effects in 33% to 59% of patients.^27,28,29^ Furthermore, in a larger trial of BA monotherapy in patients with recurrent or metastatic cervical cancer, the rate of grade 3 or higher TRAEs was 31.5%, of which 17.1% were bleeding events.^30^ Patients receiving reduced starting doses of PDS01ADC and BA had fewer grade 3 and/or 4 TRAEs and fewer responses but comparable OS (eFigure 5 in Supplement 2). Additional details are given in eAppendix 3 in Supplement 2.

Reduced dosing of PDS01ADC and BA affected response rates, with all but 1 of the responses occurring in patients with HPV-16–positive tumors. This suggests all 3 study drugs were contributors to the clinical activity observed. In previous studies, BA as monotherapy led to greater development of HPV-16–specific T-cell responses in patients deriving clinical benefit.^15,26^ In this study, the development of multifunctional HPV-16–specific T cells, particularly in patients with best overall response of stable disease or better, suggests the addition of PDS0101 vaccine and PDS01ADC boosts T-cell activity against HPV oncoproteins.

### Limitations

This study had several limitations. The single-group design and the small sample size limit the interpretability of the results. Owing to the heterogeneity of the study population, involving different primary tumor sites, HPV-16 status, and prior ICB treatment status, some analyses involve smaller subgroups and should be viewed as hypothesis generating. This also impacts our ability to identify a dose level combination optimally balancing efficacy and toxic effects for each tumor type, based on the results of this study; studies in individual cohorts would be needed for this task.

## Conclusions

This phase 1/2 nonrandomized clinical trial found the combination of PDS0101, PDS01ADC, and BA has an acceptable safety profile and showed promising clinical activity and OS outcomes in patients with advanced HPV-16–positive cancers, either ICB naive or ICB resistant, supporting further investigation of PDS0101, PDS01ADC, and simultaneous PD-L1 and TGF-β inhibition in this patient population.

## Notes

Supplement 1.
Trial Protocol

Supplement 2. **eAppendix 1.** Rationale for the Population, Combination, Dose and Schedule
eMethods.
**eAppendix 2.** Supplementary Results **eAppendix 3.** Supplementary Discussion **eTable 1.** Participant Characteristics **eTable 2.** Grade 3, Grade 4 and Serious TRAEs in the Study **eTable 3.** Grade 3 and 4 TRAEs by Dosing of PDS01ADC and Bintrafusp Alfa **eTable 4.** Overview of Objective Response Rates and Median Overall Survival Across the Patient Groups and Historic Comparator **eFigure 1.** Changes in Target Lesions During Treatment With Combination Immunotherapy in Patients With HPV-16–Positive Disease **eFigure 2.** Overall Survival (OS) by Immune Checkpoint Inhibitor (ICB) Naive or Resistant Disease Status **eFigure 3.** Schematic of Treatment and Sampling Schedule **eFigure 4.** HPV-16 Specific T Cell Responses at Baseline and During Combination Therapy in Patients With HPV-16–Positive Tumors **eFigure 5.** Overall Survival (OS) in the HPV-16–Positive ICB-Resistant Patient Subgroup by PDS01ADC and BA Dose
eReferences.

Supplement 3.
Data Sharing Statement
