# Supplementary material for: Novel Combination Immunotherapy and Clinical Activity in Patients With HPV-Associated Cancers: A Nonrandomized Clinical Trial
Source: JAMA Oncol. 2025 Feb 20;11(4):394–9. doi: 10.1001/jamaoncol.2024.6998 (PMC11843463; doi:10.1001/jamaoncol.2024.6998)
Supplement: Supplement 1. — Trial Protocol [file jamaoncol-e246998-s001.pdf]

Abbreviated Title: Combo Immunotx in HPV Cancers

CC Protocol #: *pending*

Version Date: February 3, 2020

NCT Number: *pending*

RSC Number: 2744

Title: Phase I/II Trial of Combination Immunotherapy in Subjects with Advanced HPV Associated Malignancies

NCI Principal Investigator: Julius Strauss, MD
Laboratory of Tumor Immunology and Biology (LTIB)
Center for Cancer Research (CCR)
National Cancer Institute (NCI)
[redacted]

Investigational Agents:

Drug Name:	PDS0101	MSB0011359C (M7824)	NHS-IL12 (M9241)
IND Number:	019267	019267	019267
Sponsor:	Center for Cancer Research, NCI	Center for Cancer Research, NCI	Center for Cancer Research, NCI
Manufacturer:	PDS Biotechnology	EMD Serono, Inc.	EMD Serono, Inc.

Commercial Agents: None

PRÉCIS

Background:

- Metastatic or refractory/recurrent HPV associated malignancies (cervical, anal, oropharyngeal cancers etc.) are poorly palliated by standard therapies. There is an unmet need for active treatments for these tumors.
- In a phase I trial of M7824 (NCT02517398) 15 out of 43 (34.9%) patients with HPV associated malignancies had radiographic tumor responses according to RECIST 1.1 or iRECIST.
- While the response rate observed with M7824 appears to be higher than single agent PD-1 inhibitors alone (15-20%), the majority of patients with these diseases still do not seem to benefit from immunotherapy.
- Preclinical studies suggest that the use of a combination of multiple immunotherapy agents may have improved anti-tumor efficacy.
  - Specifically, preclinical studies have shown that the combination of three immunotherapy agents (1) a therapeutic vaccine against HPV positive cancers (PDS0101), (2) a bifunctional fusion protein targeting PD-L1 and TGF beta (M7824), and (3) a tumor targeted immunocytokine (NHS-IL12) produces greater anti-tumor activity than any single or dual combination of these agents.

Objectives:

- To evaluate the objective response rate (ORR) according to Response Evaluation Criteria (RECIST 1.1) of the combination of (1) a therapeutic vaccine against HPV positive cancers (PDS0101), (2) a tumor targeted immunocytokine (NHS-IL12) and (3) a bifunctional fusion protein targeting PD-L1 and TGF beta (M7824) in subjects with advanced HPV associated malignancies.

Eligibility:

- Age $\geq$ 18 years old.
- Subjects with cytologically or histologically confirmed locally advanced or metastatic HPV associated malignancies:
  - Cervical cancers;
  - P16+ Oropharyngeal cancers;
  - Anal cancers;
  - Vulvar, vaginal, penile, and squamous cell rectal cancers;
  - Other locally advanced or metastatic solid tumors (e.g. lung, esophagus) that are known HPV+.
- Prior first line systemic therapy is required unless the patient declines standard treatment after appropriate counseling has been provided.
- Subjects must have measurable disease.
- Prior checkpoint inhibitor use is excluded.

Design:

- This is a phase I/II trial of combination immunotherapy.
- The trial will be conducted using a Simon optimal two-stage design.
- Patients will receive HPV vaccine + NHS-IL12 + M7824.
- The first six patient will be evaluable for dose limiting toxicities (DLTs) and accrual will only continue to 8 patients if less than 2 out of the first 6 pts experience a DLT.
- If three or more out of eight patients have objective responses accrual will be expanded to enroll 20 evaluable patients.

TABLE OF CONTENTS

PRÉCIS 2
TABLE OF CONTENTS 4
STATEMENT OF COMPLIANCE 7
1. INTRODUCTION 7
1.1 Study Objectives 7
1.2 Background and Rationale 8
2 ELIGIBILITY ASSESSMENT AND ENROLLMENT 29
2.1 Eligibility Criteria 29
2.2 Screening Evaluation 32
2.3 Registration Procedures 33
2.4 Baseline Evaluation 34
3 STUDY IMPLEMENTATION 34
3.1 Study Design 34
3.2 Drug Administration 38
3.3 Study Intervention Compliance 39
3.4 Dose Modifications 39
3.5 Study Calendar 49
3.6 Cost and Compensation 51
3.7 Criteria for Removal from Protocol Therapy and Off Study Criteria 51
4 CONCOMITANT MEDICATIONS/MEASURES 52
4.1 The Following Treatments Should Not Be Administered During the Trial 52
5 BIOSPECIMEN COLLECTION 53
5.1 Correlative Studies for Research/Pharmacokinetic Studies 53
5.2 Samples for Genetic/Genomic Analysis 55
5.3 Sample Storage, Tracking and Disposition 56
6 DATA COLLECTION AND EVALUATION 58
6.1 Data Collection 58
6.2 Data Sharing Plans 59
6.3 Response Criteria 59
6.4 Toxicity Criteria 66
7 NIH REPORTING REQUIREMENTS / DATA AND SAFETY MONITORING PLAN .. 66

7.1 Definitions 66
7.2 OHSRP Office of Compliance and Training / IRB Reporting 66
7.3 NIH Required Data and Safety Monitoring Plan 67
8 SPONSOR PROTOCOL SAFETY REPORTING 67
8.1 Definitions 67
8.2 Assessment of Safety Events 69
8.3 Reporting of Serious Adverse Events 69
8.4 Waiver of expedited reporting to CCR 69
8.5 Safety Reporting Criteria to the Pharmaceutical Collaborators 70
8.6 Reporting Pregnancy 70
8.7 Regulatory Reporting for Studies Conducted Under CCR-Sponsored IND 70
8.8 Sponsor Protocol Non-Adherence Reporting 70
9 CLINICAL MONITORING PLAN 71
10 STATISTICAL CONSIDERATIONS 71
10.1 Statistical Hypothesis 71
10.2 Sample Size Determination 72
10.3 Populations for Analyses 72
10.4 Statistical Analyses 73
11 COLLABORATIVE AGREEMENTS 74
11.1 Cooperative Research and Development Agreement (CRADA) 74
12 HUMAN SUBJECTS PROTECTIONS 74
12.1 Rationale For Subject Selection 74
12.2 Participation of Children 75
12.3 Participation of Subjects Unable to Give Consent 75
12.4 Risks/Benefit Assessment 75
12.5 Consent and Assent Process and Documentation 76
13 REGULATORY AND OPERATIONAL CONSIDERATIONS 77
13.1 Study Discontinuation and Closure 77
13.2 Quality Assurance and Quality Control 77
13.3 Conflict of Interest Policy 78
14 PHARMACEUTICAL AND INVESTIGATIONAL DEVICE INFORMATION 78
14.1 PDS0101 (IND # 019267) 78

14.2 M7824 (MSB0011359C) (IND # 019267) 80
14.3 NHS-IL12 (IND # 019267) 83
15 REFERENCES 85
16 APPENDICIES 89
16.1 Appendix A Performance Status Criteria 89
17 Investigator Agreement 90

STATEMENT OF COMPLIANCE

The trial will be carried out in accordance with International Conference on Harmonisation Good Clinical Practice (ICH GCP) and the following:

- United States (US) Code of Federal Regulations (CFR) applicable to clinical studies (45 CFR Part 46, 21 CFR Part 50, 21 CFR Part 56, 21 CFR Part 312, and/or 21 CFR Part 812)

National Institutes of Health (NIH)-funded investigators and clinical trial site staff who are responsible for the conduct, management, or oversight of NIH-funded clinical trials have completed Human Subjects Protection and ICH GCP Training.

The protocol, informed consent form(s), recruitment materials, and all participant materials will be submitted to the Institutional Review Board (IRB) for review and approval. Approval of both the protocol and the consent form must be obtained before any participant is enrolled. Any amendment to the protocol will require review and approval by the IRB before the changes are implemented to the study. In addition, all changes to the consent form will be IRB-approved; a determination will be made regarding whether a new consent needs to be obtained from participants who provided consent, using a previously approved consent form.

1. INTRODUCTION

1.1 STUDY OBJECTIVES

1.1.1 Primary Objective

- To evaluate the objective response rate (ORR) according to Response Evaluation Criteria (RECIST 1.1) of the combination of (1) a therapeutic vaccine against HPV positive cancers (PDS0101), (2) a tumor targeted immunocytokine NHS-IL12 (M9241) and (3) a bifunctional fusion protein targeting PD-L1 and TGF beta (M7824) in subjects with advanced HPV associated malignancies.

1.1.2 Secondary Objectives

- To evaluate the safety of the combination of (1) a therapeutic vaccine against HPV positive cancers (PDS0101), (2) a tumor targeted immunocytokine NHS-IL12 (M9241) and (3) a bifunctional fusion protein targeting PD-L1 and TGF beta (M7824) in subjects with advanced HPV associated malignancies.
- To assess progression-free survival time (PFS) according to RECIST 1.1.
- To assess overall survival (OS).
- To assess duration of response and ratio of patients that are hospitalized because of adverse events attributed to disease progression

1.1.3 Exploratory Objectives

- To conduct exploratory immunologic studies to understand and improve the administered treatment, including:
    - peripheral immune subset analysis before and on treatment;
    - soluble factors circulation (e.g. sCD27 and sCD40 ligand) before and on treatment;

- tumor tissue immune infiltration before and after treatment.

- To assess responses to therapy using iRECIST.
- To assess responses to therapy by HPV serotype or PD-L1 expression.
- To assess correlation between circulating tumor DNA and response to therapy

1.2 Background and Rationale

1.2.1 HPV Associated Malignancies

In the United States, there are more than 30,000 cases of HPV associated cancer annually [1] (**Table 1**). Metastatic HPV associated malignancies (cervical, anal, oropharyngeal cancers etc.) are often incurable and poorly palliated by standard therapies. Responses to chemotherapy are variable but generally short-lived with median PFS around 3 to 7 months [2-5]. In a Gynecologic Oncology Group randomized trial comparing four cisplatin-based doublets as first line therapy for cervical cancer the response rates were 22-29% and median PFS was 4 to 6 months with median OS 10 to 13 months [6]. The addition of bevacizumab to combination chemotherapy has been reported to increase OS by 3.7 months, but virtually all patients die of their disease within 2 years [7]. Randomized trials of second line therapy are lacking but response rates for single agents are generally reported to be less than 20% [8]. Early evidence suggests that immune checkpoint therapy also has a low response rate in this disease with a phase 1b trial (KEYSTONE 028) showing a 12.5% response rate (3/24 patients) and a phase II study showing a response rate of 13.3% (13/98 patients) to pembrolizumab in patients with recurrent or metastatic cervical cancer [9].

Table 1: Estimated annual incidence of HPV associated cancers in the US

Site	Incidence of HPV associated cancers	Cases attributed to HPV (%)
Oropharyngeal	15,738	11,000 (70.1%)
Cervix	11,771	10,700 (90.6%)
Vulvar	3554	2400 (68.8%)
Vaginal	802	600 (75%)
Penis	1168	700 (63.3%)
Rectal (squamous cell)	750	700 (91.1%)
Anus	5,010	4,600 (91.1%)
Total	38,793	30,700 (79.1%)

For oropharyngeal cancer, the best estimates of the chemotherapy responsiveness are inferred from looking at the oropharyngeal site in subset analyses from clinical trials for head and neck cancers. In a pivotal clinical trial that established platinum, 5-fluorouracil (5-FU), plus cetuximab as first line therapy in head and neck cancer, patients with oropharyngeal tumors experienced PFS of 4 to 6 months and OS of 8 to 11 months [3]. Immune checkpoint therapy has become the standard second line therapy for metastatic oropharyngeal cancer but response rates are still low with this therapy. As an example, the phase 1b trial (KEYNOTE-012) of pembrolizumab which led to its FDA approval for recurrent or metastatic head and neck squamous cell carcinoma (HNSCC) as second line therapy had a 17.7% response rate (34/192 patients). Response rates were slightly better in HPV positive HNSCC (21.9%) [10], but still occurred in only a minority of the patients.

In regard to metastatic anal cancer, only a handful of randomized trials have been performed in the last 30 years. In the metastatic setting, most of the evidence is limited to small phase II trials, retrospective series, and case reports. A recent retrospective series looking at 77 patients, 44 (55%) of whom received 5-FU in combination with cisplatin, 24 (31%) of whom received carboplatin + paclitaxel, and 11 (14%) of whom received another regimen showed a median PFS of 7 months and median OS of 22 months [5]. As with HNSCC, recent early phase trials evaluating immune checkpoint therapy in this disease have shown that responses here too are limited to around 20% of patients treated. A recent phase II trial of nivolumab for metastatic squamous cell anal cancer showed a 21% response rate (7/33 patients) and a recent phase IB trial (KEYNOTE-028) of pembrolizumab showed a 20% response rate (5/25 patients) [11]. In a recently published phase II trial, Massarelli et al. evaluated the combination of a therapeutic HPV vaccine and Nivolumab [12]. In contrast to previous single therapy immune checkpoint inhibitor trials, they found an overall response rate of 33% showing the potential benefit of Immuno-Oncology (I/O) combination therapy for these diseases.

Table 2. Response in patients treated with ISA101 and Nivolumab (Massarelli JAMA Oncol Sept 27, 2018)

Table 2. Response Overall, in Oropharyngeal Cancer, and by Treatment History[a]

	No. (%)					
		Patients With Oropharyngeal Cancer				
Response per RECIST, version 1.1	All Patients (N = 24)	All (n = 22)	Platin-Refractory Disease (n = 17)	Cetuximab-Refractory Disease (n = 8)	Platin- and Cetuximab-Refractory Disease (n = 6)	Received Second-line Treatment (n = 12)
Overall response rate	8 (33)	8 (36)	6 (35)	5 (63)	3 (50)	5 (42)
Complete response	2 (8)	2 (9)	2 (12)	1 (13)	0	2 (17)
Partial response	6 (25)	6 (27)	4 (24)	4 (50)	3 (50)	3 (25)
Stable disease	3 (13)	2 (9)	1 (6)	0	1 (17)	1 (8)
Disease control rate[b]	11 (46)	10 (45)	7 (41)	5 (63)	4 (67)	6 (50)
Progression of disease	13 (54)	12 (55)	10 (59)	3 (38)	2 (33)	6 (50)

Abbreviation: RECIST, Response Evaluation Criteria in Solid Tumors.

[a] Refractory indicates progression of disease within 6 months of treatment.

[b] Disease control rate is calculated by adding the percentages of patients whose tumors exhibited complete response, partial response, and stable disease and indicates lack of progression per RECIST.

Unfortunately, little data exists evaluating immunotherapy for rarer HPV associated malignancies including metastatic vulvar, vaginal, penile, squamous cell rectal or neuroendocrine cervical cancer.

1.2.2 Rationale for Combination of I/O Agents

Preclinical studies are now revealing that an effective immuno-oncology strategy for enhancing anti-tumor responses is the use of multiple immune-mediating agents, each targeting different components of the immune system. These include: (a) induction of an immune response via vaccination directed against tumor-associated antigens, (b) reduction of immunosuppressive entities in the TME by the use of anti-PD1/PDL1 MAb checkpoint inhibitors, and/or reduction of immunosuppressive cytokines such as TGF-β with the use of a bifunctional anti-PDL1/TGF-β2 "Trap" designated M7824, and (c) potentiation of the immune response in the TME by the use of the tumor targeting NHS-IL12 immunocytokine.

The LTIB, in collaboration with the GMB, has been able to develop and/or co-develop these agents via a series of CRADAs. Each has been interrogated in a series of preclinical studies and in Phase I/II clinical studies in the CCR as well as by other investigators. Preclinical studies and early clinical studies are demonstrating the relative lack of additional toxicities with enhanced clinical benefit employing combinations of 2 of these agents. Preclinical studies are now demonstrating that the combined use of agents from each of the immune-mediating categories described above elicits the most immune-mediated anti-tumor activities.

1.2.2.1 HPV Vaccine

PDS0101 is a novel nanoparticle-based immunotherapy (therapeutic vaccine) developed to treat genital cancers such as anal, cervical, vulvar cancer etc. and their precancers (late-stage dysplasia/neoplasia), as well as head and neck cancers caused by infection with the human papilloma virus (HPV). PDS0101 is a subcutaneously administered therapy consisting of two vialed components mixed by the investigator at the time of administration. The immunotherapy is composed of liposomal nanoparticles of the pure immunologically active R enantiomer of the cationic (positively charged) lipid DOTAP (dioleoyl-trimethylammonium propane) mixed with 6 lipidated antigenic peptides derived from the HPV-16 E6 and E7 proteins.

In-vivo and in-vitro studies performed in normal and transgenic mouse models as well as in-vitro studies using human lymphocytes in culture demonstrated potent immune modulating effects by R-DOTAP. The pure R stereoisomer of DOTAP acts as a potent activator of the MAP kinase signaling pathway leading to activation and maturation of dendritic cells and subsequent induction of the production and release of cytokines and chemokines critical for promotion of an effective immune response. The positive charge on the surface of R-DOTAP liposomes facilitates their uptake by the dendritic cell. This in turn facilitates uptake of the HPV peptide antigen micelles and the antigenic processing and presentation by dendritic cells to T-cells. The ability of cationic liposomes to destabilize endosomes facilitates antigen delivery into the cytoplasm of the dendritic cells and other antigen presenting cells [13]. Effective induction of cytotoxic T-lymphocyte (CTL) responses and tumor regression has been demonstrated in the pre-clinical studies.

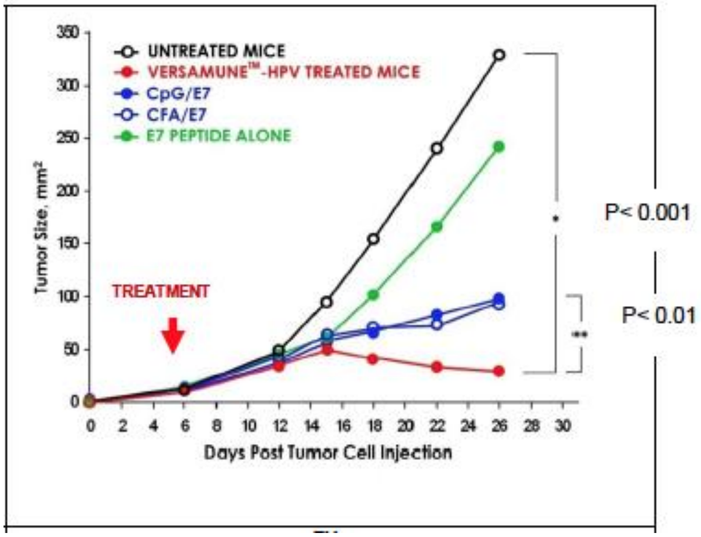

Figure 1. Anti-tumor efficacy of DOTAP/E7 vs. CpG-E7 and CFA-E7 in C57BL/6J mice bearing TC-1 HPV+ tumors.

Toxicology studies of R-DOTAP and a prototype R-DOTAP/HPV16-E7 single peptide formulation were evaluated in rats and in non-human primates (monkeys). No deaths or clinical signs related to treatment were observed in any of the studies. No treatment effects on body weight were noted. No treatment-related and/or toxicologically significant effects for any clinical chemistry parameters evaluated were noted during any of the studies. All hematology parameters, red blood cell counts, hemoglobin, mean corpuscular volume, mean corpuscular hemoglobin and mean corpuscular hemoglobin concentration remained normal.

Toxicology of the PDS0101 clinical formulation (6-peptide HPVmix and ImmunoMAPKRDOTAP) was evaluated in humanized transgenic mice. The transgenic mice expressed the human HLA-A2 antigen recognition and presentation molecule, and their antigen presenting cells were therefore capable of recognizing and responding to the HPV A2 epitope human peptide antigens in the drug formulation. Similar to toxicology studies in rats and monkeys with the prototype drug formulation, few clinically relevant toxicological effects were observed at any dose level in the transgenic humanized mice. However, for the clinical PDS0101 formulation mean hematocrit was decreased in the low R-DOTAP + HPVmix and high R-DOTAP + HPV mix groups by approximately 3% and 5%, respectively; and although minimal, may be related to treatment. No treatment-related toxicological findings were observed during gross necropsy. Microscopic histopathologic findings (minimal fibrosis) considered related to treatment with the test article were limited to the skin at the injection site in 3 out of 8 in the high R-DOTAP + HPV mix group only.

In 2014-2016, a Phase I/IIA human clinical trial was performed at 3 sites in the US in 12 subjects with high-risk HPV infection and biopsy-proven cervical intraepithelial neoplasia (CIN1) to study the safety and immunogenicity of PDS0101.The study initiated with Cohort 1 and progressed through Cohort 3, with each subsequent cohort receiving a higher dose of PDS0101 (i.e., 1.0, 3.0, and 10.0 mg R-DOTAP); 3 in Cohort 1 (1-mg R-DOTAP dose), 3 in Cohort 2 (3 mg R-DOTAP dose), and 6 in Cohort 3 (10-mg RDOTAP dose). Successive cohorts received a constant dose of

2.4 mg HPV-16 E6 and E7 peptides (400 µg/peptide) with 1 of 3 escalating doses of R-DOTAP. All subjects received 3 doses of vaccine given SC, approximately 21 days apart. 10/12 subjects had a positive vaccine-induced response (a ≥3-fold increase over baseline at ≥1 of the 4 post-vaccination visits by either the IFN-γ or granzyme B ELISpot assays, with background counts subtracted). Individual subject results from both the IFN-γ and the granzyme B assays showed that all doses of RDOTAP were effective in inducing HPV-specific T-cell responses (**Figure 2A**). IFN-γ (all T-cells) and the granzyme B (CD8+ T-cells) responses were elicited in both HPV-16+ and HPV-16- subjects, and in subjects with various HLA types.

Administration of PDS0101 was also well tolerated. The majority of AEs were due to administration site reactions. Most administration site reactions were mild or moderate in severity. Some more severe administration site reactions including swelling and redness were reported in the 3- and 10-mg R-DOTAP cohorts. Most administration site reactions resolved the same day or within a few days. Per the subject symptoms diary, administration site reactions were more severe and of longer duration in subjects in the 10-mg R-DOTAP cohort. No clinically significant differences in the types and pattern of TEAEs were observed between Vaccinations 1, 2, or 3. No DLTs were observed; thus, the MTD was the 10-mg R-DOTAP dose. No serious adverse events (SAEs), study discontinuations due to adverse events, or deaths occurred. No clinically relevant abnormal hematology, blood chemistry, urinalysis, or physical findings were observed. Regression of CIN was noted in a number of patients (**Figure 2B**). Regression was seen in CIN related to a variety of HPV types including HPV-16, HPV-18 as well as other high-risk HPV types. The 3-mg R-DOTAP PDS0101 dose (containing 2.7 mg HPV-16 E6 & E7 antigens) has been chosen for further evaluation in larger Phase II trials given it seems to afford the best combination of safety and potency. The original dose of HPV-16 E6 & E7 antigens has been minimally increased from 2.4 mg to 2.7 mg for planned phase II studies. This was done due to some concern with possible oxidation of one of the peptides at the original lower dose. For simplicity the dose of all peptides has been minimally increased.

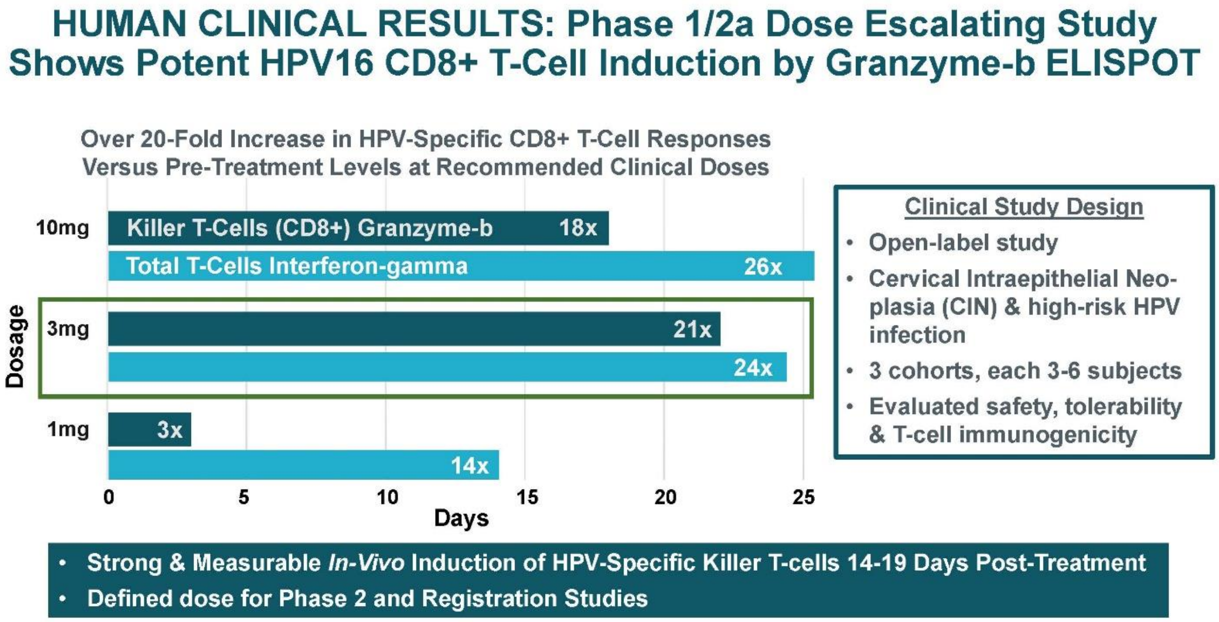

HUMAN CLINICAL RESULTS: PDS0101 Induced Possible Strong Clinical Benefit Independent of Patient Genetic Sub-Type

Cohort	Low Dose (1.0mg)			Mid Dose (3.0mg)			High Dose (10.0mg)					
Subject	005-001	005-002	005-004	002-001	002-002	002-003	002-004	002-005	002-007	003-001	003-002	005-007
HPV DNA Type(s) at Baseline	68	31	**16**, 31, 35, 39, 52, 56	**16**, 58	**16**, 51, 53	33, 52, 54, 58, 83	**16**	52	18, 52, 58	35, 52, 58	42, 52	52, 58
HLA-Type	02	01, 02	01	02	29	03, 30	02, 26	02, 68	02, 03	74, 80	02, 30	N/A
T-Cell Response	✓	High Baseline	✓	✓	High Baseline	✓	✓	✓	✓	✓	✓	✓
HPV DNA Clearance (*not* type specific)	✓	✓	No	Pending	Pending	Pending	Pending	NF	Pending	NF	NF	✓
Earliest Documented Clearance. s/p Vaccine #3 (months)	3m	1m	---									13m
Regression (ASCUS or Normal)	✓	✓	No	Pending	Pending	Pending	Pending		Pending			✓
Earliest Documented Regression s/p Vaccine #3 (months)	3m	1m	---									13m

NF (No Follow-up) – One patient at site 002 was lost to follow-up at D93 of study.
Data from the 2 patients at site 003 (Augusta Cancer Center) were unavailable for post-study follow up and confirmation of regression or viral clearance

Confidential

Figure 2 A (top). ELISpot responses in patients treated with PDS0101. **B (bottom).** CIN Regression in patients treated with PDS0101.

Avelumab (anti-PD-L1) and M7824 (anti-PD-L1/TGF- βR2)

Studies in the LITB were the first to describe [14, 15] avelumab, a fully human anti-PD-L1 IgG1 MAb, and its ability to mediate ADCC for a range of human carcinoma cells employing NK cells as effectors. Avelumab (Bavencio) has since been approved by the FDA for the therapy of Merkel cell carcinoma (the first drug approved for this indication) and second line urothelial carcinomas. We have also shown that endogenous IL-12 will enhance avelumab-mediated NK lysis, thus forming the basis for the combined use of avelumab or M7824 with NHS-IL12. The first-in-human clinical study of avelumab with over 1,700 patients was led by Gulley with the dose escalation and initial PK and PD done entirely within the CCR [15]. Because of the activity this study was amended to include multiple expansion cohorts internationally. Because PD-L1 is also expressed on some immune cell subsets, our finding [16] that there were no adverse events in the patients treated with avelumab above that seen with other anti-PD-L/PD-L1 MAbs was important for planning further studies with avelumab or M7824.

TGF-β is a well-known inhibitor of immune cell function, especially in the TME [17-22]. Prior studies have indicated the advantage in the use of a TGF-β inhibitor in combination with checkpoint therapy [23, 24] In collaboration with our CRADA partner EMD Serono, we have supported the development of a bifunctional fusion protein aimed at bringing a TGF-β TRAP to tumor cells via binding to PD-L1. M7824 is a novel fusion protein consisting of anti PD-L1 antibody linked to the extracellular domain of two TGF-βR2 (serving as a TGF-β TRAP) (**Figure 3**).

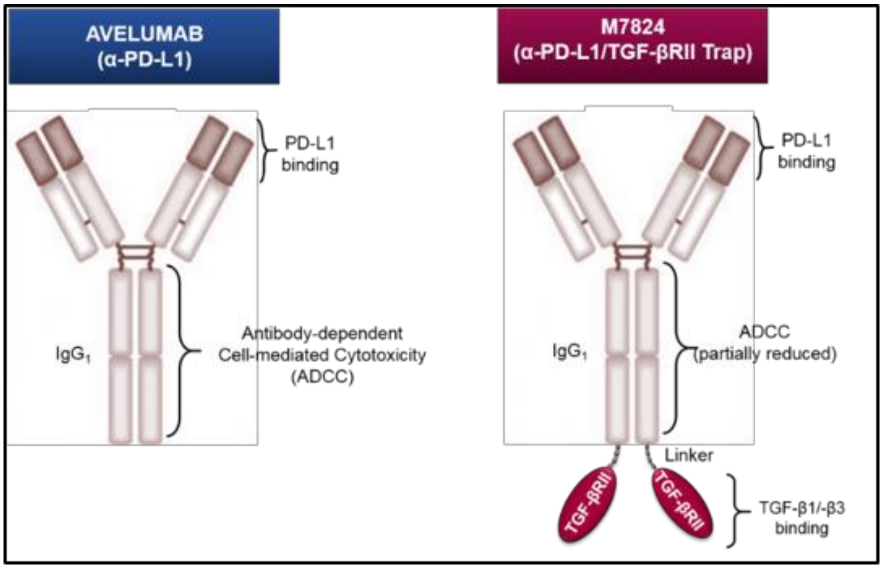

Figure 3. Schematic drawings of Avelumab and M7824.

We were the first to report [25, 26] that M7824 (a) mediates ADCC, (b) increases tumor cell gene expression of molecules involved in T-cell trafficking to the tumor, (c) enhances TRAIL- and antigen-specific CD8+ T-cell lysis of tumor cells, and (d) reduces TGF-β–induced immunosuppressive activity. We have also recently demonstrated [27] in in vivo preclinical murine carcinoma models (**Figure 4A and B**) that M7824 has the ability to decrease TGF-β–induced signaling in the TME. Specifically, phosphorylation of SMAD2 was significantly

decreased in tumors following M7824 treatment, and not with an M7824(mut) molecule, devoid of the PD-L1 binding site (**Figure 4C**). In both breast and colon carcinoma murine models, M7824 decreased tumor burden and increased survival, which required both CD8+ and NK cell activity (**Figure 4D**).

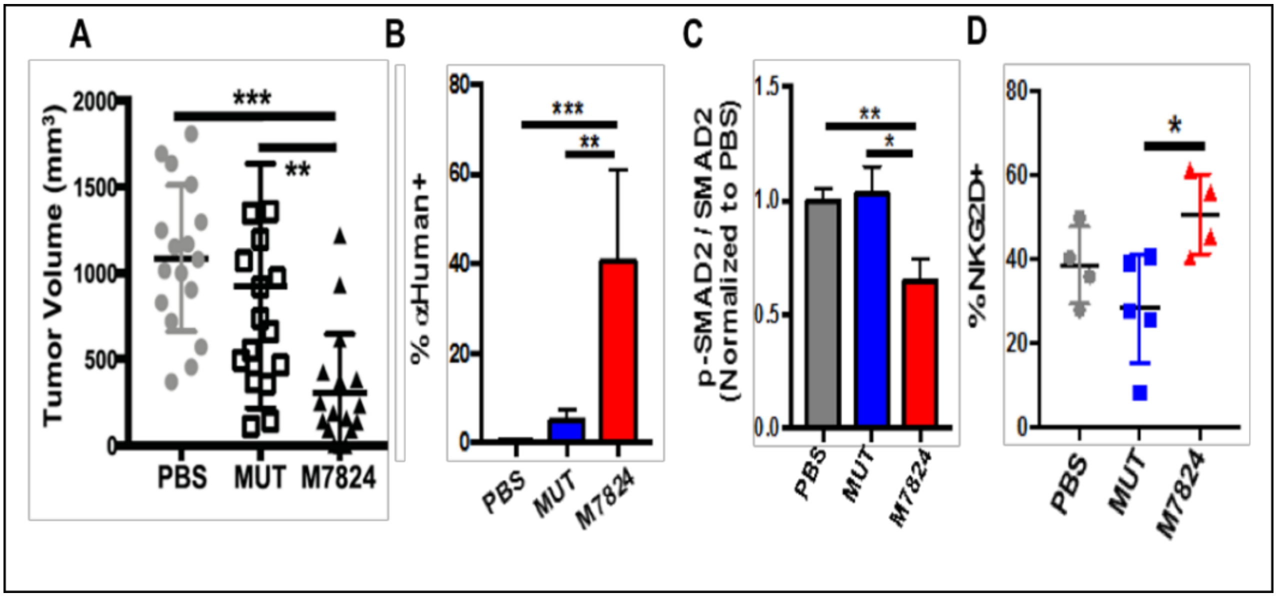

Figure 4 (A) Comparison of anti-tumor activity of M7824, and M7824(mut) devoid of PD-L1 binding site in the EMT6 breast cancer model. **(B)** Accumulation of M7824 in the TME resulting in reduction of SMAD2 signaling **(C)** and increased T- and NK-cell activation **(D).** Knudson …Schlom J. OncoImmunology. 2018:e1426519.

Two recent studies [28, 29] demonstrated the advantage in anti-tumor activity in the use of the bifunctional agent vs. the use of a PD- 1/PD-L1 checkpoint in combination with a systemic TGF-β inhibitor. TGF-β is also known to alter human carcinoma cells from a more epithelial to a more mesenchymal phenotype and such tumor cells are more resistant to a range of therapies (**Figure 5**). In LTIB studies, TGF-β–induced mesenchymalization of carcinoma cells was shown [30] to be reversed by M7824, rendering tumor cells more susceptible to chemotherapy and immune-mediated killing [25, 27, 30]

The first-in-human trial of M7824 (anti-PD-L1/TGF-βR2) was conducted at the CCR [31]. Nineteen heavily pretreated patients with solid tumors (non-melanoma) were treated; the MTD was not reached. M7824 was shown to saturate peripheral PD-L1 and sequester all released (acid released) plasma TGFβ1, -β2, and -β3 throughout the dosing period at >1 mg/kg (recommended phase 2 dose is 1200 mg or about 10 mg/kg). There were signs of clinical efficacy across all dose levels (**Figure 6** and **Figure 7**), including one ongoing CR (cervical cancer), two durable PRs (pancreatic and anal cancers), one near PR (cervical) and two cases of prolonged stable disease.

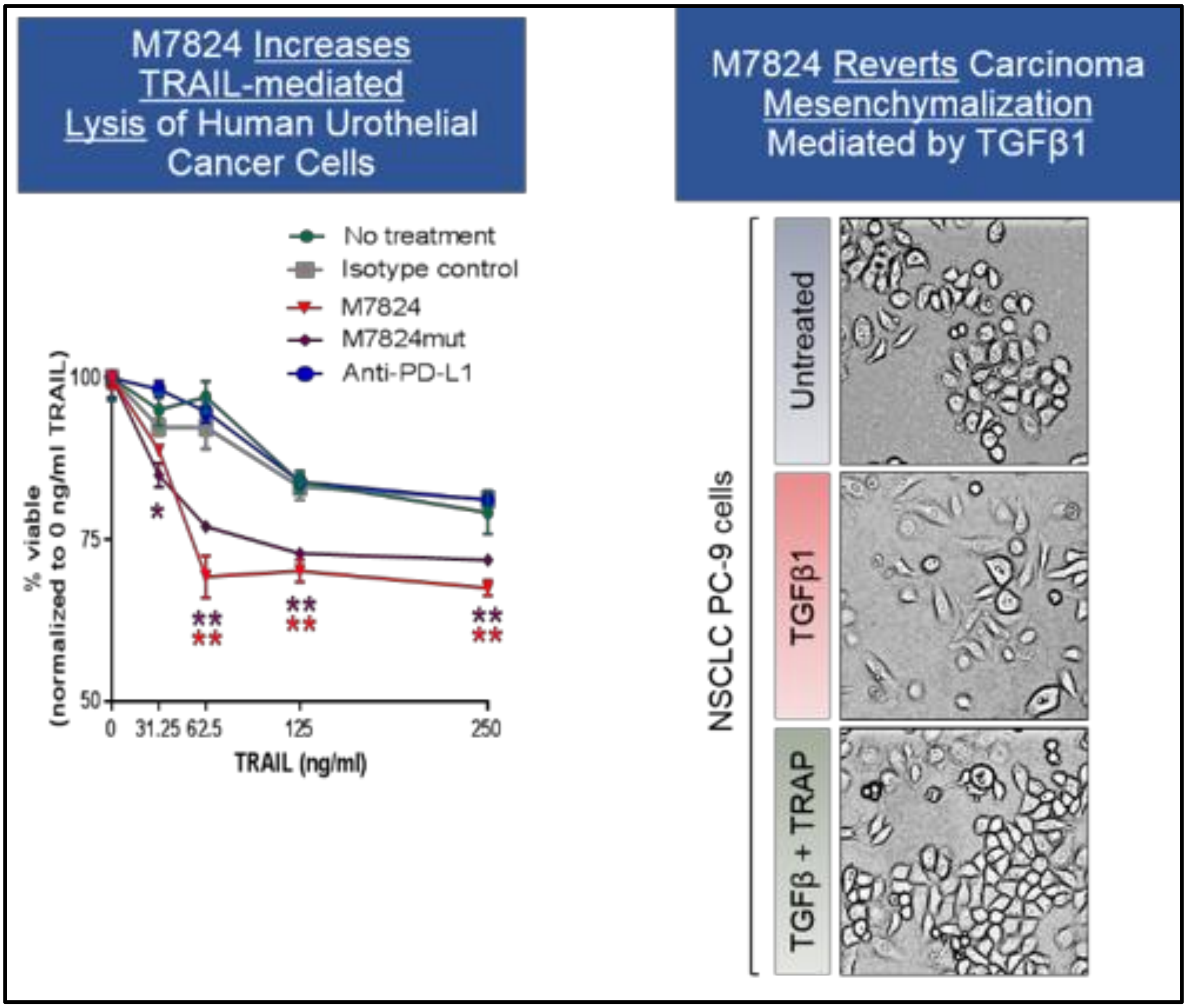

Figure 5. Functions of M7824.

Phase I Trial of M7824 (anti-PD-L1/TGFβR2)

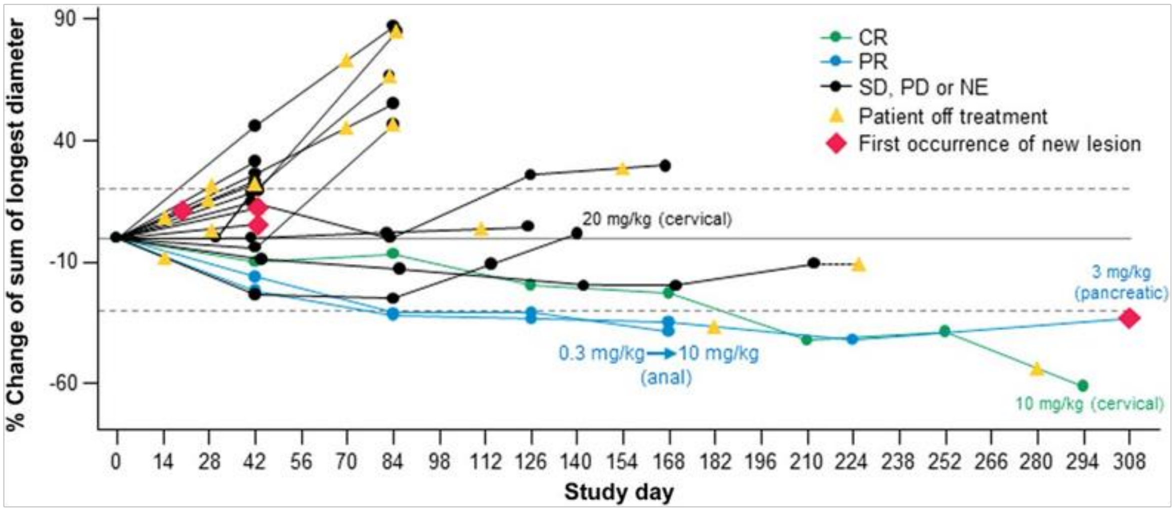

Strauss…Schlom…Gulley. Clin Cancer Res. 2018;24:1287-95.

M7824 Reduction of Circulating TGF-β

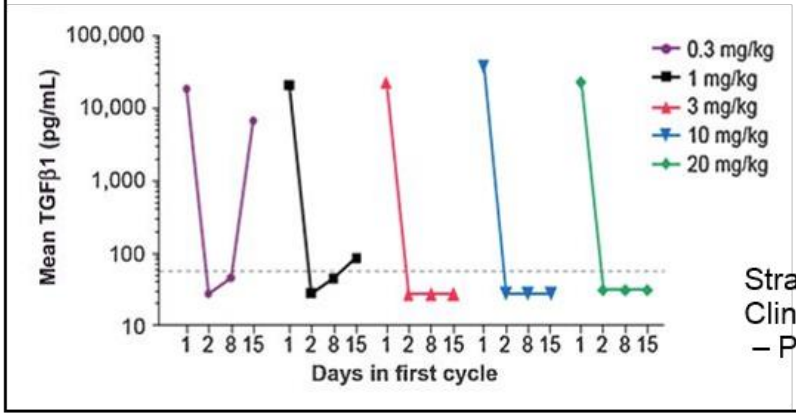

Strauss…Donahue, Schlom…Gulley.
Clin Cancer Res. 2018;24:1287-95.
– Phase I Trial: M7824

Figure 6. Phase I trial of M7824, spider plot of tumor responses and reduction of serum levels of TGFβ1.

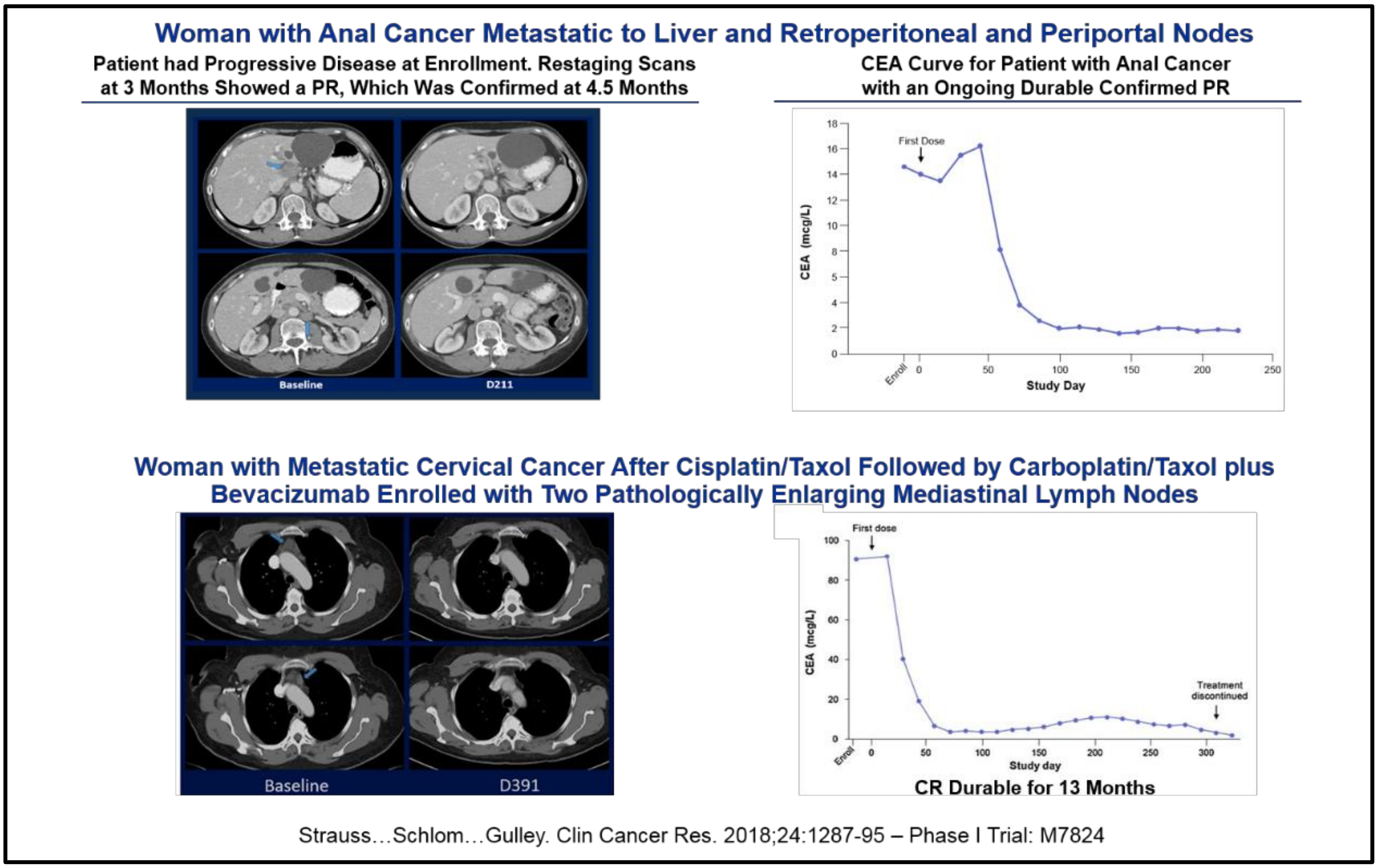

Figure 7. M7824 Phase I, responses in two patient**s.**

In the Phase I study [31] 15/43 (34.9%) of patients with HPV associated malignancies had radiographic tumor responses according to RECIST 1.1 or iRECIST with M7824 (anti-PD-L1/TGF-βR2) therapy. Prior studies by others have shown approximately a 15% objective response in this population employing checkpoint inhibition therapy.

Sufficient peripheral blood mononuclear cells were available from 12 patients on the phase I trial to test for HPV-specific T-cell responses before and after M7824 treatment. Considering all post-therapy timepoints examined, 7 of 12 patients (58%) had an increase in HPV-specific T cells post- vs pre-M7824 therapy. Immune responses after 1 and 3 cycles of M7824 were compared between patients who developed a clinical response of stable disease or better vs those with progressive disease (**Figure 8**). In clinical responders, HPV-specific T cells developed in 3 of 4 patients (75%) after 1 cycle of M7824 and in 4 of 4 patients (100%) after 3 cycles. On the other hand, in patients with progressive disease, HPV-specific T cells developed in only 1 of 5 patients (20%) after 1 cycle of M7824 and in 1 of 4 patients (25%) after 3 cycles (Supplementary **Table 3**). There was also an increased magnitude of HPV-specific T cells generated in clinical responders vs nonresponders: clinical responders had on average 12- to 27-fold more HPV-specific T cells producing cytokines or positive for CD107a (LAMP1, a functional marker of T and NK cell activity) after 1 and 3 cycles of M7824 compared with nonresponders (**Figure 3A**).

Another observation worth noting is that 2 of the 3 patients with HPV+ cancer who received prior lymphodepleting chemotherapy (e.g., cyclophosphamide, fludarabine) were assessed for immune responses post- vs pre-M7824 therapy, and neither had evidence of an HPV T-cell–specific immune response. In contrast, in patients with HPV+ cancer who did not receive prior lymphodepleting chemotherapy, 6 of 9 (67%) had a positive T-cell–specific immune response.

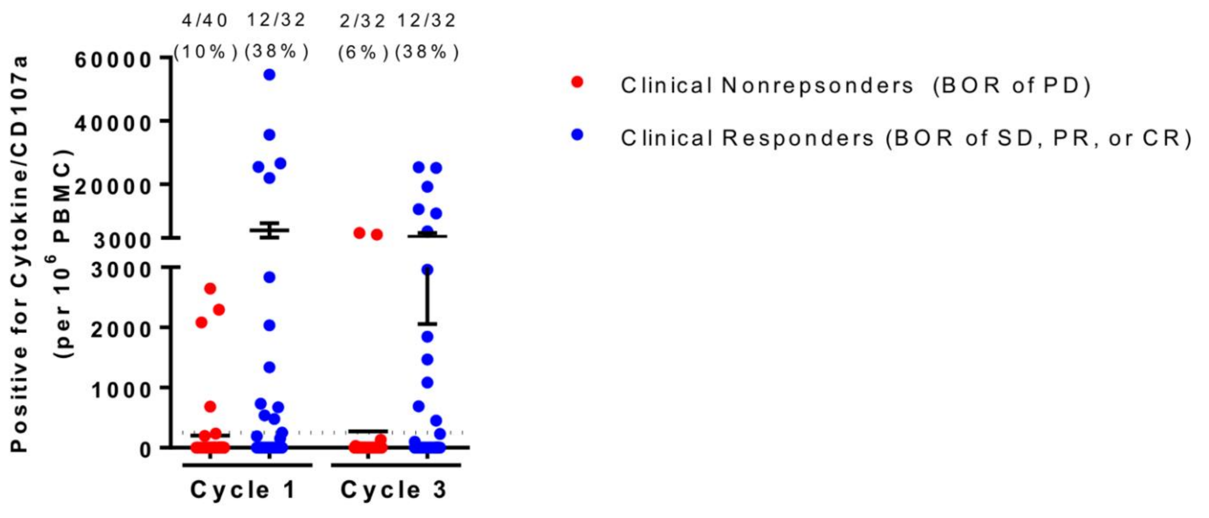

Figure 8. Immune responses in HPV+ patients with disease control (BOR of SD, PR, or CR) vs nonresponders (BOR of PD) to M7824 therapy by intracellular cytokine staining following in vitro stimulation with a mixture of overlapping 15-mer peptide pools encoding HPV-16 E6 and E7, as previously described.[21]

NHS-IL12 Immunocytokine (M9241)

NHS-IL12 is a tumor targeting immunocytokine that binds to DNA-histone in necrotic areas of tumor. Studies by others have shown the ability of radiolabeled NHS-IL12 to target murine and human tumors (**Figure 9**). Prior studies in the LTIB have shown the ability of this agent to elicit anti-tumor activity in multiple murine models, and in combination with anti-PD-L1 and with radiation and chemotherapy [32]. Gulley (GMB, CCR) and the LTIB conducted the Phase I dose escalation trial of the NHS-IL12 immunocytokine [33]. Due to known prior toxicities using a rec.

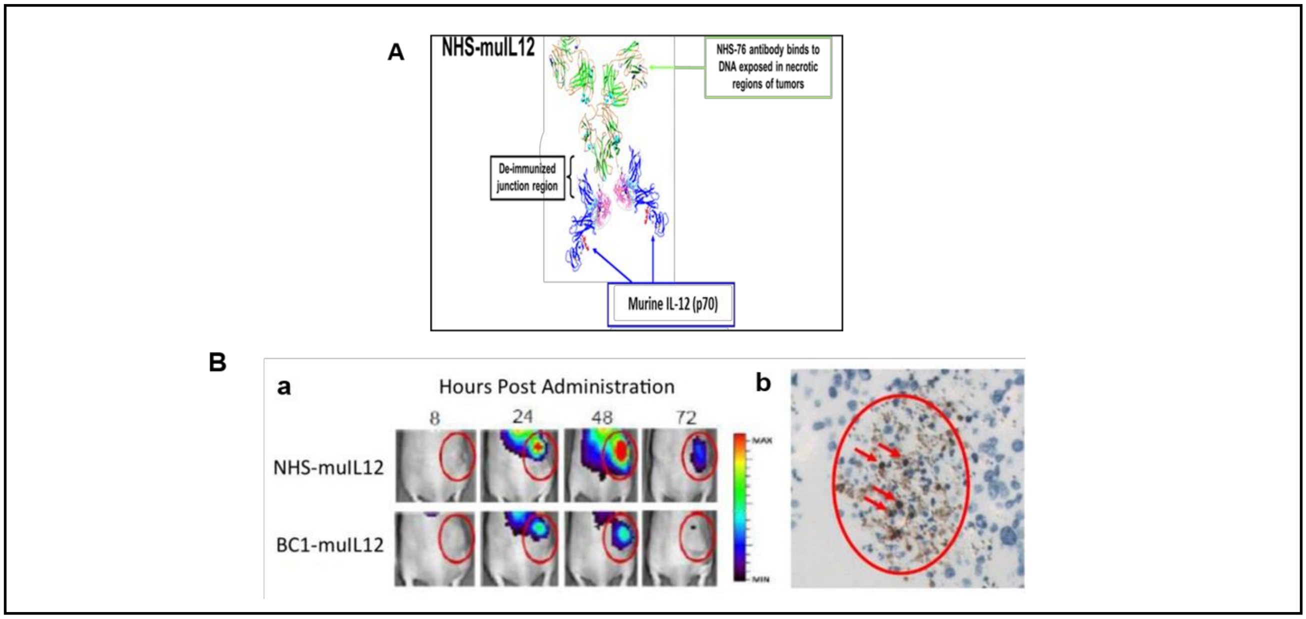

Figure 9. NHS-IL12 Immunocytokine. **(A)** NHS76 is a fully human 2nd generation TNT antibody bound to 2 murine IL-12 (p70) molecules. **(B) a:** Specific tumor targeting of transplanted lung carcinoma by the MAb NHS-IL12(mu). Control MAb BC1-IL12(mu). **b:** NHS-IL12 tumor targeting of nuclear DNA histones.

IL-12 protein, multiple ascending dose levels were employed and the agent was administered every 4 weeks. Time-dependent increase in serum IFN-γ and subsequent rise in IL-10 increased with dose and varied greatly among patients (**Figure 10A**). Increases in TCR diversity and TIL density in the TME following NHS-IL12 dosing were observed in those patients with elevation IFN-γ serum levels (**Figure 10B**). Toxicity was acceptable with an MTD of 16.8 mcg/kg. Although no objective tumor responses were seen, of 30 patients with measurable disease, 15 had stable disease, with some durable (from 6–30+ months).

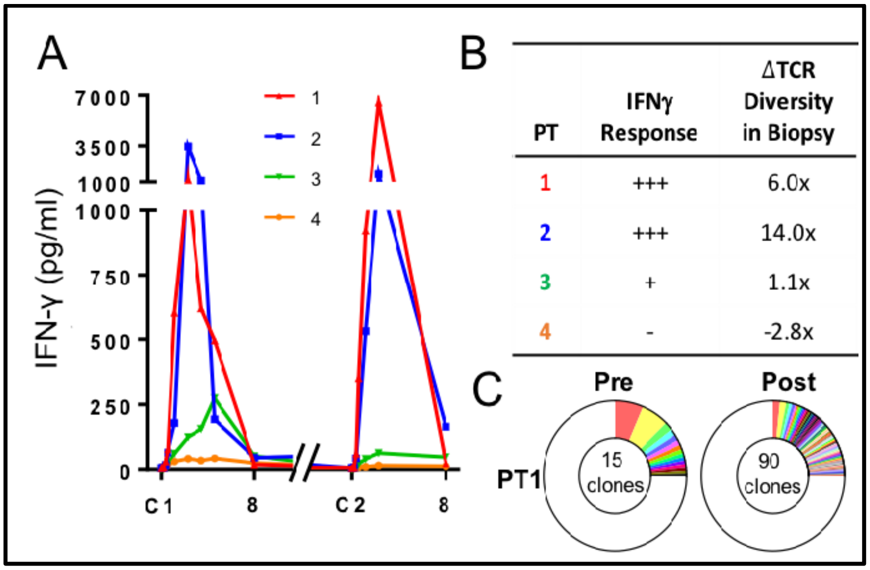

Figure 10. (A) Diversity of IFN-γ spikes in sera among patients 3 days post-NHS-IL12 treatment for cycles 1 (C1) and 2 (C2): as examples, 2 patients with high (pts 1 and 2, red and blue) and low (pts 3 and 4, green and orange) levels are shown. **(B)** Changes in TCR clonal diversity in the tumor biopsy correlate with serum IFN-g responses post-NHS-IL12 treatment. **(C)** # of clones in tumor comprising the top 25% of the repertoire of patient 1 pre- and post-NHS-IL12, demonstrating an increase in TCR diversity in a high IFN-g responder after therapy.

Our recent preclinical studies have also shown the enhanced anti-tumor effect of avelumab when used in combination with NHS-IL12 in syngeneic mice bearing an intravesical bladder tumor **(Figure 11, left panel)** and that this effect is mediated via an IFN-γ–dependent mechanism. Studies in NSG-β2m⁻/⁻ mice bearing HTB1, a human bladder carcinoma, and reconstituted with human PBMC, also showed the enhanced anti-tumor effect of the combination that coincided with increased NK and $CD8^+$ in the TME (**Figure 11, right panel**).

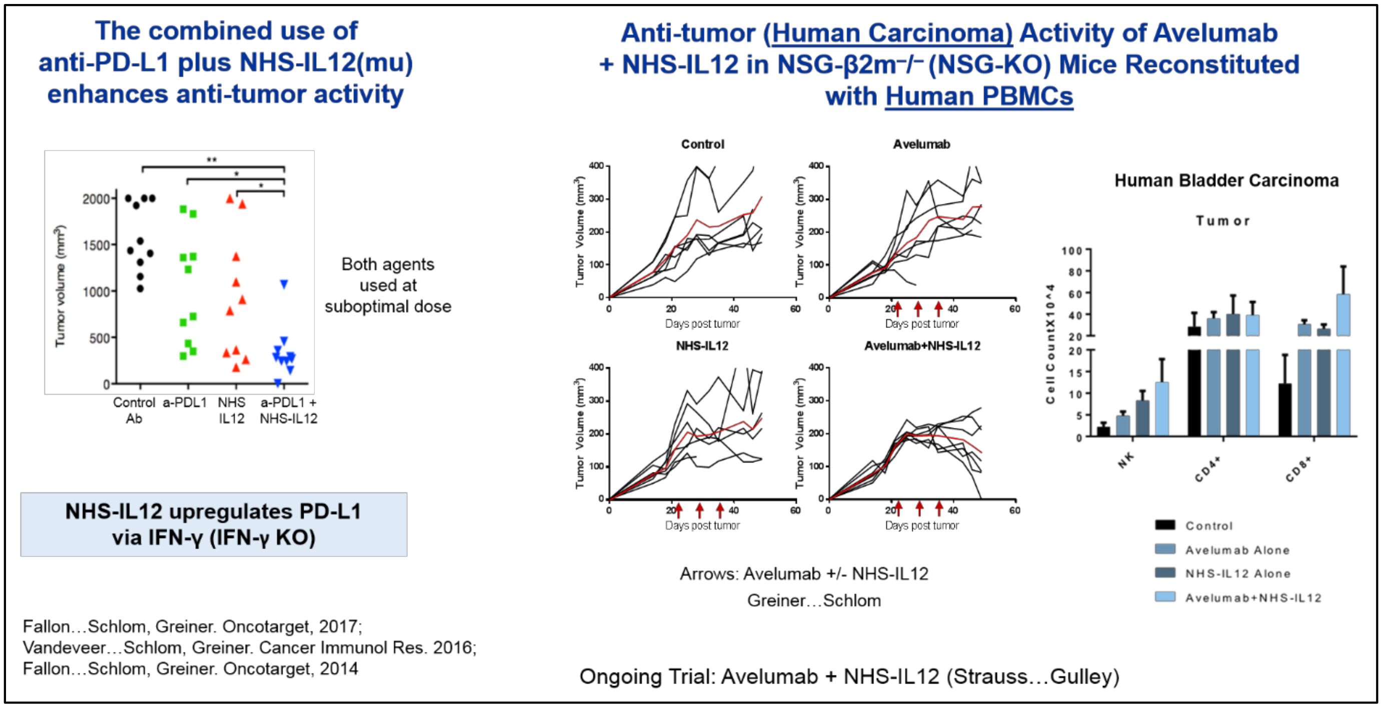

Figure 11. Anti-tumor activity of NHS-IL12 + anti-PD-L1 in NSG-β2m⁻/⁻ mice bearing human bladder cancer (HTB1).

This further provided the rationale for an ongoing CCR led clinical study of avelumab in combination with NHS-IL12 (NCT02994953), currently evaluating safety of the combination. In addition, to the combination being found to be safe and well-tolerated, evidence of radiographic tumor responses has been observed. As an example, a patient with checkpoint refractory metastatic urothelial cancer with a large sacral mass and retroperitoneal adenopathy at enrollment has had an ongoing complete response for 18+ months.

Combination Immunotherapies

In addition to harnessing the potential multi-functionality of M7824, we have conducted several combination studies employing this agent with other immune modulating agents. We have previously shown [26] enhanced anti-tumor activity when M7824 was combined with vaccine in EMT6, a metastatic breast carcinoma murine model (**Figure 12**).

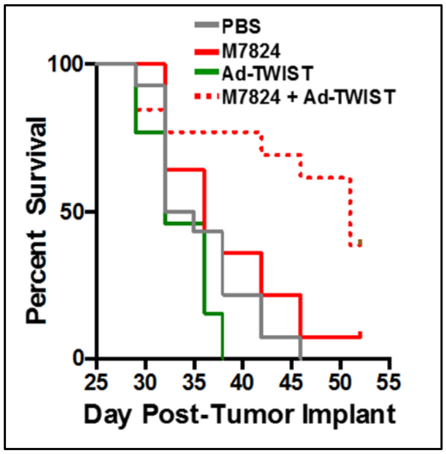

Figure 12. Combined effect of Ad-Twist vaccine and M7824 in EMT6 breast carcinoma model (Knudson, 2018)

Furthermore, combining M7824 with NHS-IL12 also greatly enhanced anti-tumor activity in the EMT6 breast carcinoma model (**Figure 13**). Tumor challenge studies showed the presence of immune memory in mice that received the combination of M7824 and NHS-IL12, which conferred protection against re-challenge with tumor. Similar results were also seen in the MB38-CEA murine colorectal carcinoma model.

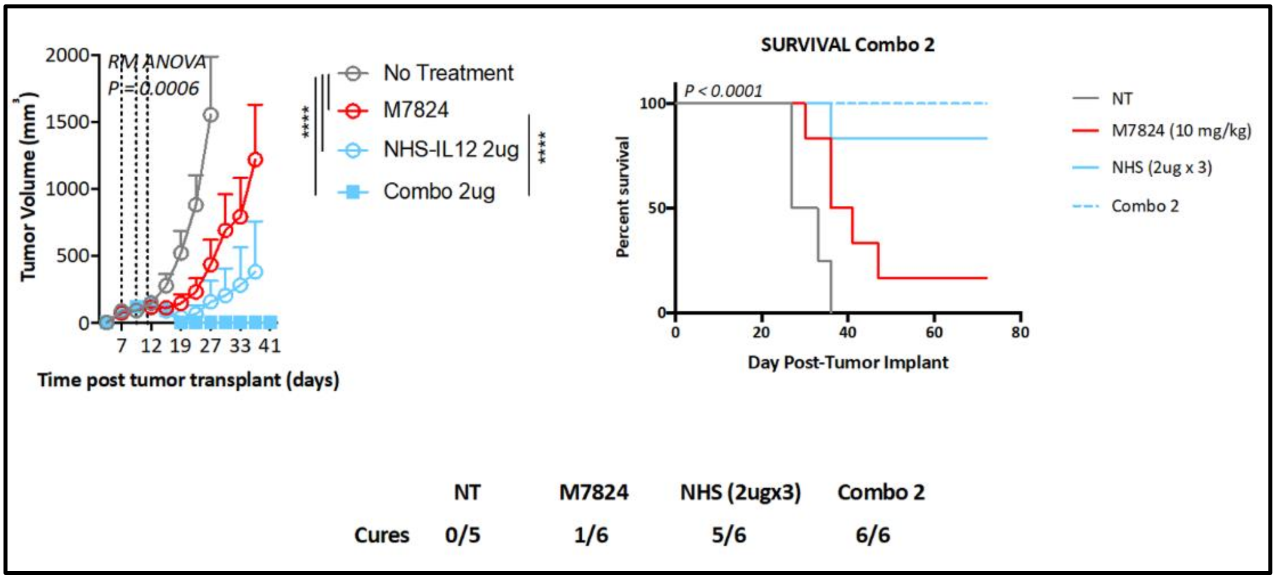

Figure 13. Anti-tumor effect of M7824 + NHS-IL12 combination treatment in EMT6

Multimodal therapies were also carried out in mice bearing HPV⁺ tumors. The combination of PDS0101 vaccine and M7824 showed significant anti-tumor efficacy in the NSG $\beta 2m^{-/-}$ humanized mouse model reconstituted with human PBMC and bearing human cervical HPV^{+} cancer (SiHa) subcutaneously (**Figure 14**).

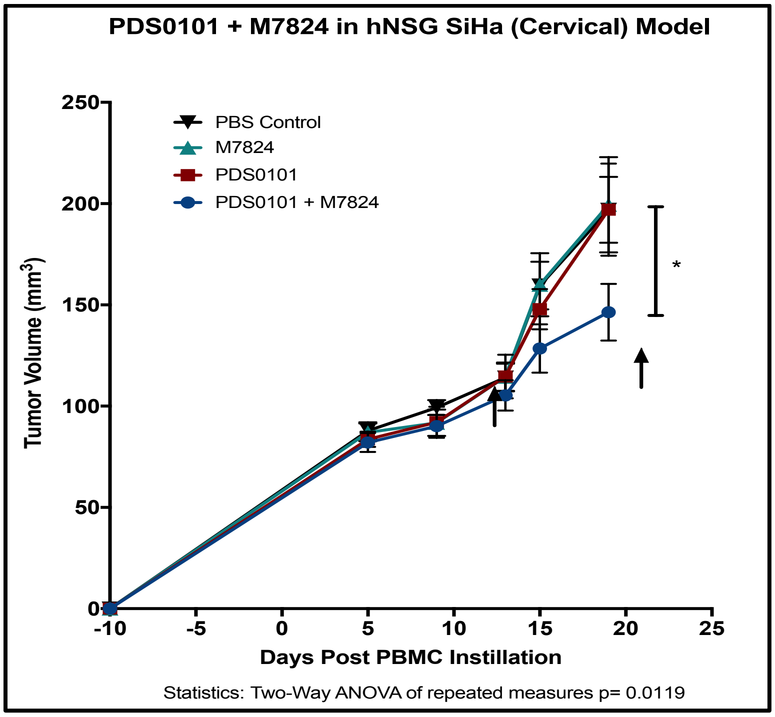

Treatment	Tumor volume >200 mm^3
PBS Control	6/12
M7824	4/12
PDS0101	5/12
PDS0101+M7824	1/12

Figure 14. Tumor growth in humanized mice bearing SiHa (human HPV$^+$ cervical cancer) and treated with PBS control, PDS0101 vaccine, M7824, or PDS0101+M7824.

In contrast to the previous clinical trials, where we saw anti-tumor efficacy of M7824 or PDS0101 alone in HPV-associated tumors, in this model there was no single therapy effect of M7824 or PDS0101, but the combination significantly decreased tumor burden. Furthermore, in C57BL/6J mice bearing TC-1 (a murine lung carcinoma line transfected with HPV16 E6 and E7 oncoproteins), which is a very aggressive model of HPV$^+$ carcinoma, the combination of PDS0101 vaccine, M7824 and NHS-IL12 resulted in the greatest level of anti-tumor activity compared to the use of individual agents or dual combinations (**Figure 15**). The use of the combination of PDS0101 vaccine, M7824 and NHS-IL12 also resulted in an intense influx of CD4$^+$ and CD8$^+$ T cells in tumors (**Figure 16, upper panels** and **Figure 17**), increased activation and proliferation of T-cells (**Figure 16, lower panels**), as well as an enhanced HPV specific T cell response measured by ELISpot not seen with lesser combinations (**Figure 18**).

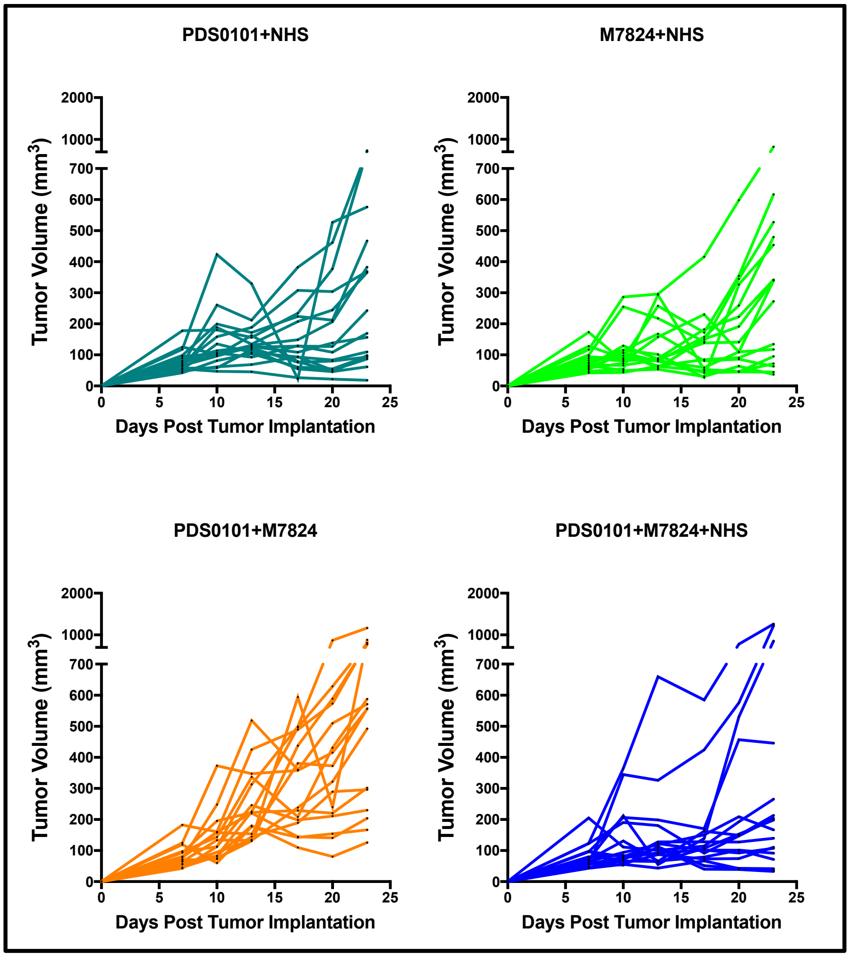

Figure 15 A. Tumor growth in C57BL/6J mice bearing TC-1 HPV+ tumors, and treated with combinations of PDS0101, M7824, and NHS-IL12.

Treatment	Tumor Volume <300mm^3
PBS Control	0/16
DOTAP	0/8
M7824	0/16
PDS0101	3/16
NHS	6/16
M7824+NHS	8/16
PDS0101+NHS	10/16
PDS0101+M7824	5/16
PDS0101+M7824+NHS	13/17

Figure 15 B. Tumor control (low volume) in C57BL/6J mice bearing TC-1 HPV+ tumors, and treated with combinations of PDS0101, M7824, and NHS-IL12.

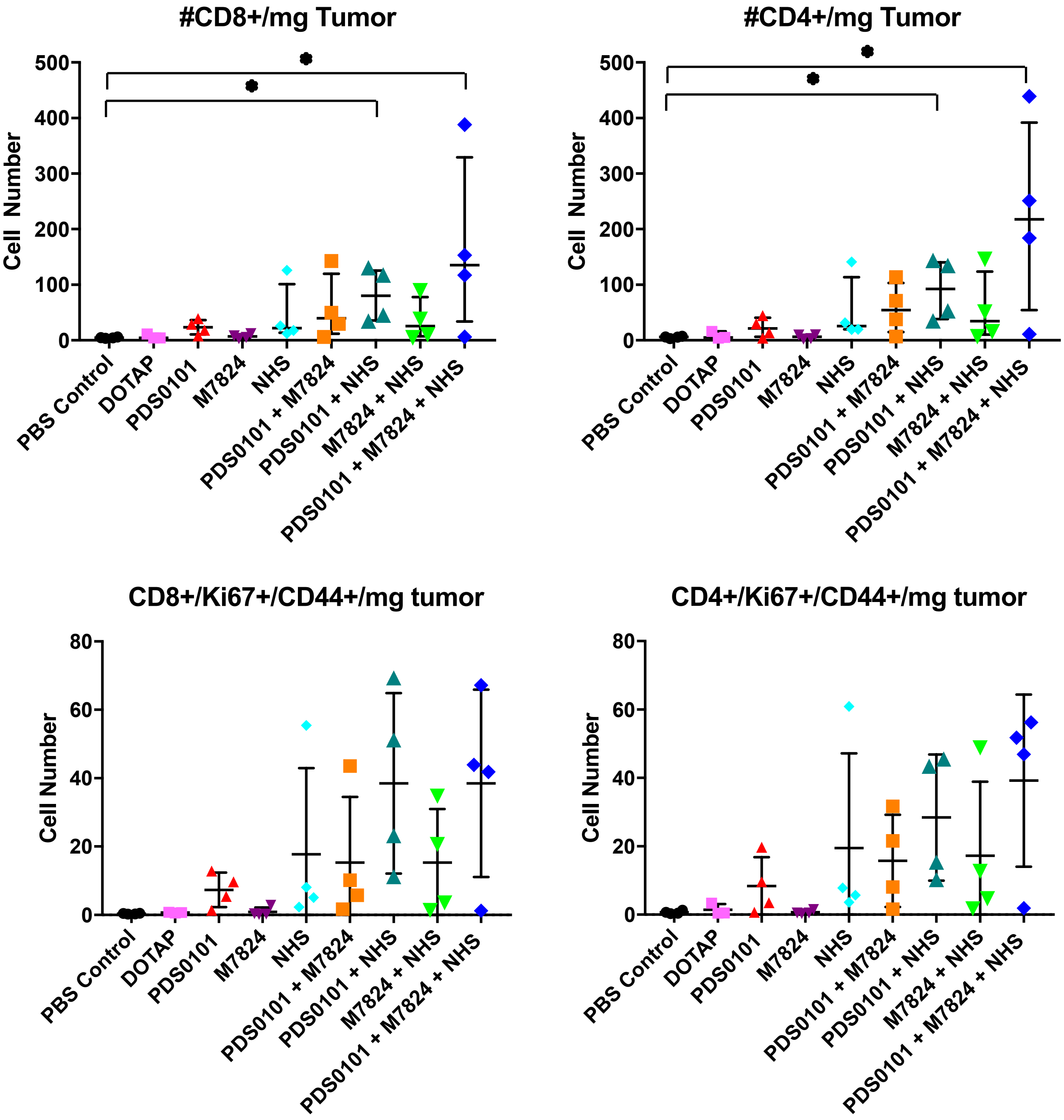

Figure 16. Increased T-cell infiltration by tumor weight, and increased T-cell activation and proliferation in combination treated mice. (Rumfield et al; unpublished)

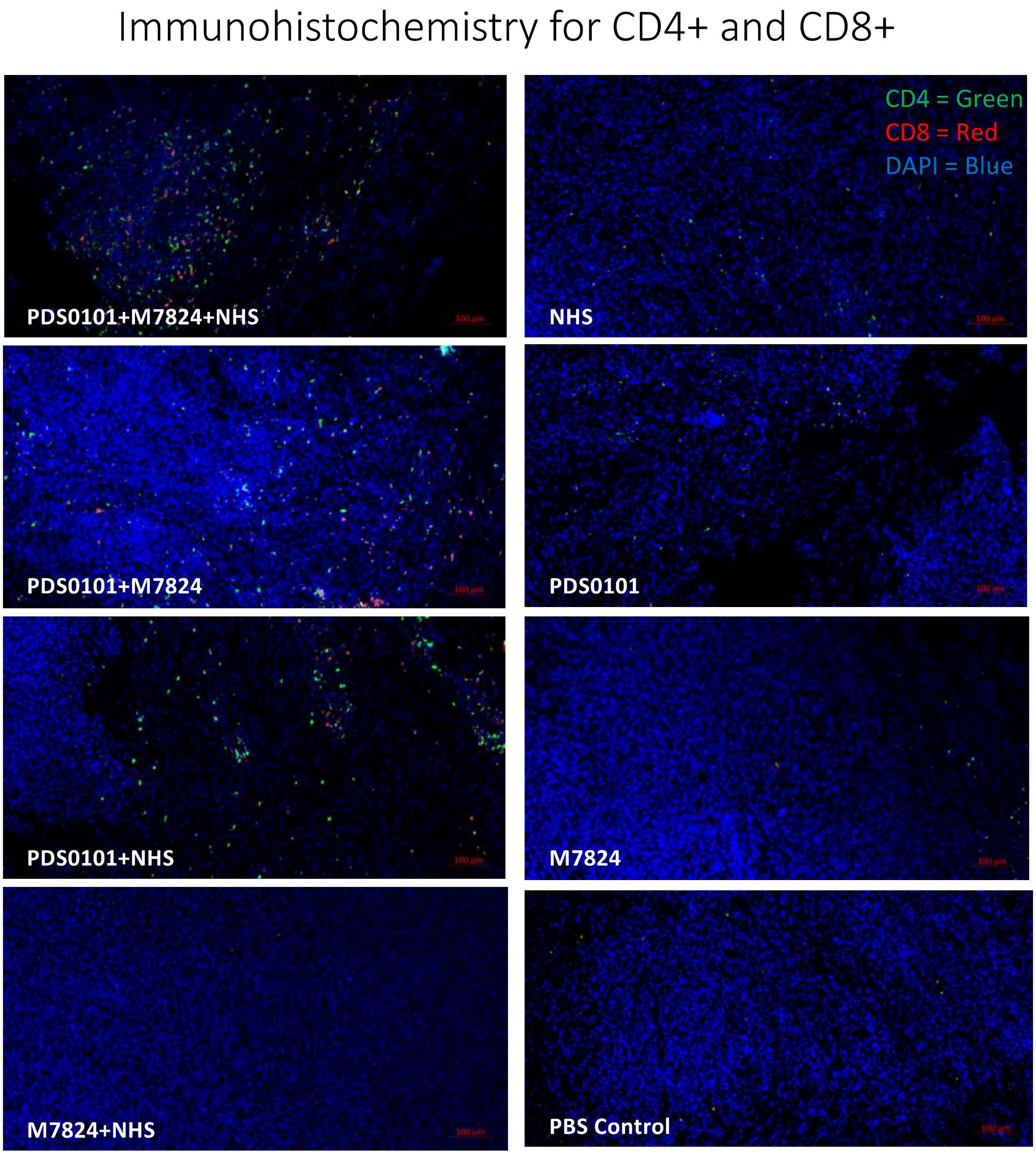

Figure 17. Immunohistochemistry shows increased T-cell infiltration in C57BL/6J mice bearing TC-1 HPV+ tumors, and treated with the combination of PDS0101, M7824, and NHS-IL12.

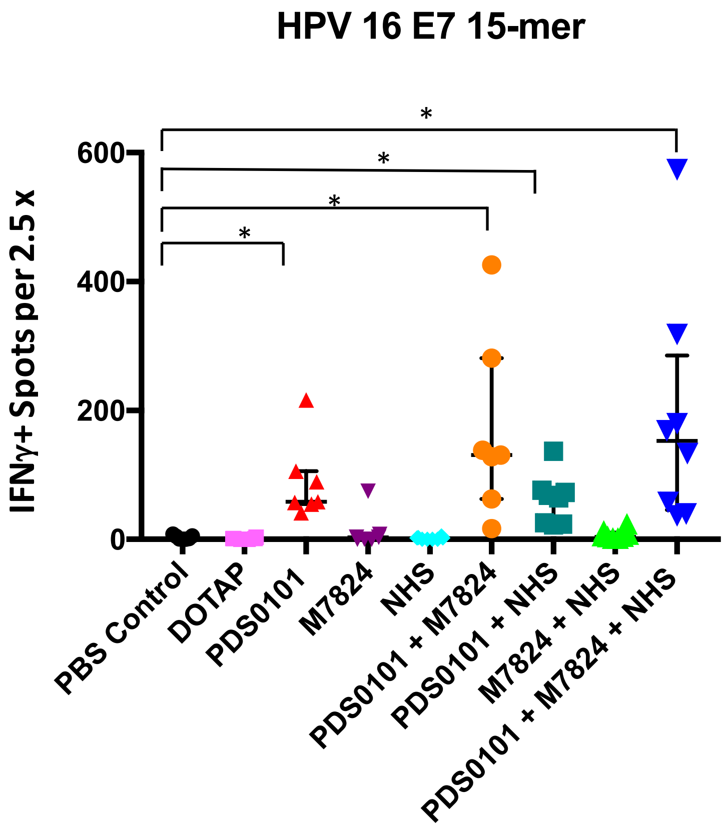

Treatment	Tumor Volume <300mm³	E7 ELISpot
PBS Control	0/16	-
DOTAP	0/8	-
M7824	0/16	-
PDS0101	3/16	+
NHS	6/16	-
M7824+NHS	8/16	-
PDS0101+NHS	10/16	+
PDS0101+M7824	5/16	+
PDS0101+M7824+NHS	13/17	-

Figure 18. PDS0101 generated HPV-specific T-cells, as measured by ELISpot. Table shows a summary of tumor volume and E7-specific IFNγ ELISpot for C57BL/6J mice bearing TC-1 HPV+ tumors, and treated with combinations of PDS0101, M7824, and NHS-IL12

1.2.3 Safety and Efficacy of I/O Combinations in Humans

Table 3: Previously evaluated combinations of I/O agents demonstrating an acceptable safety profile.

Avelumab (anti-PD-L1) + NHS-IL12	NCT02994953
CEA/ MUC1 vaccine + M7824	NCT03315871

Dose Rational: In the phase I dose escalation trial of combination Avelumab (anti-PD-L1) + NHS-IL12, escalating doses of NHS-IL12 were evaluated at 8,12, and 16.8 mcg/kg given every 4 weeks in combination with standard dosing of Avelumab. The combination including NHS-IL12 at the highest dose evaluated (16.8 mcg/kg given every 4 weeks) was found to be well tolerated. The only DLT observed on the trial was a transient grade 3 AST/ALT elevation at NHS-IL12 at 12 mcg/kg but otherwise no other DLT was observed and the MTD was not reached. Therefore, for

this protocol, we plan to use NHS-IL12 at a dose of 16.8 mcg/kg every 4 weeks in combination with the standard phase II dose of M7824 of 1200 mg given every two weeks.

1.2.4 Immune Assays

Multiple immune assays have been developed in the LTIB to better define the mechanism(s) involved in the use of specific novel agents, as monotherapy or in combination therapies, both for preclinical and clinical studies. In addition to analyses of biopsies, analyses of the peripheral immunome can provide valuable information in that multiple samples can be analyzed over the course of a given therapy vs. pre-therapy. The LTIB has now developed and employed [14, 15, 30, 34] a flow cytometry–based assay that can analyze 123 immune cell subsets in human PBMC from one vial of processed PBMC (approximately 107 cells). This assay will be used in this clinical trial to detect multiple (n=32) subsets of CD4+ T cells, CD8+ T cells (n=29), Tregs (n=7), B cells (n=5), NK (n=20), NKT (n=4), DC (n=10), and MDSC (n=16) to better understand the role of each agent. We also plan to evaluate changes in TCR clonal diversity both in biopsies and the periphery. One example involves an ongoing first-in-human trial of the NHS-IL12 tumor-targeting immunocytokine. TCR diversity increased 6-14–fold in biopsies of patients with a high or intermediate IFN-γ response but were unchanged or decreased with a low IFN-γ response. These findings also correlated with TIL in biopsies. We are also currently employing NanoString analyses to identify a gene signature in biopsies and PBMC, pre- and post-treatment. The GMB will conduct multiplexed, multispectral imaging of FFPE tissue to evaluate multiple immune parameters within the TME before and after treatment in patients with available tissue.

1.2.5 Summary

- We are proposing a novel I/O combination to help overcome multiple barriers in an effective anti-tumor immune response.
    - (1) HPV therapeutic vaccine targeting tumor associated antigens to prime an anti-tumor immune response
    - (2) NHS-IL12 and M7824 to enable activated immune cells to work better in the TME
- Preclinical studies have shown that treatment with the combination of an HPV therapeutic vaccine, M7824 and NHS-IL12 leads to greater anti-tumor activity than any single or double combination of these agents

2 ELIGIBILITY ASSESSMENT AND ENROLLMENT

2.1 ELIGIBILITY CRITERIA

2.1.1 Inclusion Criteria

2.1.1.1 Subjects with cytologically or histologically confirmed locally advanced or metastatic HPV associated malignancies:

- Cervical cancers;
- P16+ Oropharyngeal cancers;
- Anal cancers;
- Vulvar, vaginal, penile, and squamous cell rectal cancers;
- Other locally advanced or metastatic solid tumors (e.g., lung, esophagus) that are known HPV+.

2.1.1.2 Subjects must have measurable disease, per RECIST 1.1. See Section 6.3 for the evaluation of measurable disease.

2.1.1.3 Subjects must have received prior first line systemic therapy unless the patient is not eligible to receive standard therapy or declines standard treatment.

2.1.1.4 Age ≥18 years.

2.1.1.5 ECOG performance status ≤2.

2.1.1.6 Adequate hematologic function at screening, as follows:

- Absolute neutrophil count (ANC) ≥1 x 10^9/L;
- Hemoglobin ≥ 9 g/dL;
- Platelets ≥ 75,000/microliter.

2.1.1.7 Adequate renal and hepatic function at screening, as follows:

- Serum creatinine ≤ 1.5 x upper limit of normal (ULN) OR
  Measured or calculated creatinine clearance ≥ 40 mL/min for participant with creatinine levels > 1.5 X institutional ULN (GFR can also be used in place of creatinine or CrCl);
- Bilirubin ≤ 1.5 x ULN OR in subjects with Gilbert's syndrome, a total bilirubin ≤ 3.0 x ULN;
- Alanine aminotransferase (ALT) and aspartate aminotransferase (AST) ≤ 2.5 x ULN, unless liver metastases are present, then values must be ≤ 3 x ULN).

2.1.1.8 The effects of the immunotherapies on the developing human fetus are unknown. For this reason and because immunotherapeutic agents as well as other therapeutic agents used in this trial are known to be teratogenic, women of child-bearing potential and men must agree to use highly effective contraception (hormonal or barrier method of birth control; abstinence) prior to study entry and for two months after study treatment. Should a woman become pregnant or suspect she is pregnant while she or her partner is participating in this study, she should inform her treating physician immediately.

2.1.1.9 Patients serologically positive for HIV, Hep B, Hep C are eligible as long as the viral loads are undetectable by quantitative PCR. HIV positive patients must have CD4 count ≥ 200 cells per cubic millimeter at enrollment, be on stable antiretroviral therapy for at least 4 weeks and have no reported opportunistic infections or castleman's disease within 12 months prior to enrollment.

2.1.2 Exclusion Criteria

2.1.2.1 Patients with prior investigational drug, chemotherapy, immunotherapy or any prior radiotherapy (except for palliative bone directed therapy) within the past 28 days prior to the first drug administration except if the investigator has assessed that all residual treatment-related toxicities have resolved or are minimal and feel the patient is

otherwise suitable for enrollment. Patients may continue adjuvant hormonal therapy in the setting of a definitively treated cancer (e.g. breast cancer).

2.1.2.2 Prior checkpoint inhibitor therapy.

2.1.2.3 Major surgery within 28 days prior to the first drug administration (minimally invasive procedures such as diagnostic biopsies are permitted).

2.1.2.4 Known active brain or central nervous system metastasis (less than a month out from definitive radiotherapy or surgery), seizures requiring anticonvulsant treatment (<3 months) or clinically significant cerebrovascular accident (<3 months). In order to be eligible patients must have repeat CNS imaging at least a month after definitive treatment showing stable CNS disease. Patients with evidence of intratumoral or peritumoral hemorrhage on baseline imaging are also excluded unless the hemorrhage is grade ≤ 1 and has been shown to be stable on two consecutive imaging scans.

2.1.2.5 Pregnant women are excluded from this study because these drugs have not been tested in pregnant women and there is potential for teratogenic or abortifacient effects. Because there is an unknown but potential risk for adverse events in nursing infants secondary to treatment of the mother with these immunotherapies, breastfeeding should be discontinued if the mother is treated on this protocol.

2.1.2.6 Active autoimmune disease that might deteriorate when receiving an immunostimulatory agent with exception of:

- Diabetes type I, eczema, vitiligo, alopecia, psoriasis, hypo- or hyperthyroid disease or other mild autoimmune disorders not requiring immunosuppressive treatment;
- Subjects requiring hormone replacement with corticosteroids are eligible if the steroids are administered only for the purpose of hormonal replacement and at doses ≤ 10 mg of prednisone or equivalent per day;
- Administration of steroids for other conditions through a route known to result in a minimal systemic exposure (topical, intranasal, intro-ocular, or inhalation) is acceptable;
- Subjects on systemic intravenous or oral corticosteroid therapy with the exception of physiologic doses of corticosteroids (≤ the equivalent of prednisone 10 mg/day) or other immunosuppressives such as azathioprine or cyclosporin A are excluded on the basis of potential immune suppression. For these subjects these excluded treatments must be discontinued at least 1 weeks prior to enrollment for recent short course use (≤ 14 days) or discontinued at least 4 weeks prior to enrollment for long term use (> 14 days). In addition, the use of corticosteroids as premedication for contrast-enhanced studies is allowed prior to enrollment and on study.

2.1.2.7 Subjects with a history of serious intercurrent chronic or acute illness, such as cardiac or pulmonary disease, hepatic disease, bleeding diathesis or recent (within 3 months) clinically significant bleeding events, or other illness considered by the Investigator as high risk for investigational drug treatment.

2.1.2.8 History of non-HPV associated second malignancy within 3 years of enrollment except localized malignancy which has been adequately treated or malignancy which does not

require active systemic treatment (e.g,, low risk CCL). Patients taking adjuvant hormonal therapy for definitively treated cancers (e.g. breast cancer) are eligible.

2.1.2.9 Subjects with a known severe hypersensitivity reaction to a monoclonal antibodies (grade >/= 3 NCI-CTCAE v5) will be evaluated by the allergy/immunology team prior to enrollment.

2.1.2.10 Receipt of prior lymphodepleting chemotherapy (e.g. cyclophosphamide, fludarabine) or any organ transplantation requiring ongoing immunosuppression.

2.1.3 Recruitment Strategies

This study will be posted on www.clinicaltrials.gov, NIH websites and on NIH social media forums. After IRB approval of the recruitment material, this study may be advertised at national conferences including AACR and ASCO and may also be shared with the HPV and Anal Cancer Foundation who may advertise it further.

Participants will be recruited from the current patient population at NIH. Patients with HPV related cancers are currently being treated on another trial through LTIB, CCR, NIH, where 2-3 patients per month are being screening and enrolled. Some of these patients come from established community networks, some of these patients are self-referrals, and some of these patients are referred by another group (Dr. Hinrichs's group, ETIB, CCR, NIH) who also have a large referral base for HPV related cancers.

2.2 SCREENING EVALUATION

2.2.1 Screening activities prior to the obtaining informed consent

Minimal risk activities that may be performed before the subject has signed a consent include the following:

- Email, written, in person or telephone communications with prospective subjects
- Review of existing medical records to include H&P, laboratory studies, etc.
- Review of existing MRI, x-ray, or CT images
- Review of existing photographs or videos
- Review of existing pathology specimens/reports from a specimen obtained for diagnostic purposes

2.2.2 Screening activities performed after a consent for screening has been signed

The following activities will be performed only after the subject has signed the consent for study # 01C0129 on which screening activities will be performed within 28 days prior to enrollment:

- Complete medical history and physical examination (including height, weight, vital signs, and ECOG performance status).
- CT of chest, abdomen and pelvis or MRI.
- A brain CT / MRI scan if clinically indicated.
- Nuclear bone scan if clinically indicated.

- For skin lesions, documentation by color photography, including a ruler to estimate the size of the lesion.
- EKG
- Clinical laboratory tests (within 16 days prior to enrollment).
  - Chemistry: sodium, potassium, chloride, bicarbonate, calcium, glucose, BUN, creatinine, ALT, AST, alkaline phosphatase, total protein, albumin, and total and direct bilirubin.
  - Hematology: complete blood count (CBC) with differential.
  - CD4 (if clinically indicated).
- Urine or serum pregnancy test (β-HCG) for females of childbearing-potential and women < 12 months since the onset of menopause (within 16 days prior to enrollment).
- Urinalysis.
- HBV, HCV, HIV testing including viral load if clinically indicated (within 3 months prior to enrollment).
- Histologic confirmation (at any time point prior to enrollment). If there is no available tumor sample or pathology report, a biopsy will be performed to confirm the diagnosis.

2.3 REGISTRATION PROCEDURES

Patient registration will be a single step process as these patients are screened on the general NIH screening protocol (01-C-0129). Authorized staff must register an eligible candidate with NCI Central Registration Office (CRO) within 24 hours of signing consent. A registration Eligibility Checklist from the web site (http://home.ccr.cancer.gov/intra/eligibility/welcome.htm) must be completed and sent via encrypted email to: NCI Central Registration Office [redacted] fter confirmation of eligibility at Central Registration Office, CRO staff will call pharmacy to advise them of the acceptance of the patient on the protocol prior to the release of any investigational agents. Verification of Registration will be forwarded electronically via e-mail to the research team. A recorder is available during non-working hours.

Subjects that do not meet screening criteria should be removed from the study following the procedure in Section **3.7.1**.

2.3.1 Treatment Assignment Procedures

Cohorts

Number	Name	Description
1	Cohort 1	Subjects with HPV associated malignancies

Arms

Number	Name	Description
1	Arm 1	HPV vaccine + NHS-IL12 + M7824

Arm Assignment

Patients in Cohort 1 will be assigned to Arm 1. Assuming that more than three of eight patients have an objective response then accrual will be expanded to enroll 20 evaluable patients.

2.4 BASELINE EVALUATION

All subjects are required to complete baseline evaluations within one week prior to the first planned dosing of the study drug (any screening evaluation done within this time period can also serve for the baseline evaluation):

- Physical exam including weight, ECOG performance status and vital signs.
- Concomitant Medications and Baseline Signs and Symptoms evaluation.
- Urine or serum pregnancy test (β-HCG) for females of childbearing-potential and women < 12 months since the onset of menopause.
- Chemistry: sodium, potassium, chloride, bicarbonate, calcium, glucose, BUN, creatinine, ALT, AST, alkaline phosphatase, total protein, albumin, and total and direct bilirubin.
- ACTH, TSH, free T4, lipase, amylase, CRP
- Hematology: CBC with differential
- Coagulation panel: PT, INR, and PTT

3 STUDY IMPLEMENTATION

3.1 STUDY DESIGN

- This is a phase I/II trial of combination immunotherapy.
- The trial will be conducted using a Simon optimal two-stage design.
- Patients will receive: HPV vaccine + NHSIL12 + M7824 (**Figure 19**).
- The first six patient will be evaluable for dose limiting toxicities (DLTs) and accrual will only continue to 8 patients if less than 2 out of the first 6 pts experience a DLT.
- If three or more out of eight patients have objective responses then accrual will be expanded to enroll 20 evaluable patients (**Figure 20**).
- There will be a minimum of six days between the first and second patient. The third through sixth patient may enroll no sooner than 48 hours after the most recent enrollee. Once six patients have enrolled and been found to be safe further patients may enroll at any time.
- Although positive HPV testing will not be required prior to enrolling, HPV testing will be offered as an exploratory endpoint and patients testing negative for HPV after enrolling or whose HPV status cannot be confirmed may be replaced with other patients for the primary

efficacy analysis. Patients testing negative for HPV after enrolling or whose HPV status cannot be confirmed may continue to receive treatment on study.

- The combination of PDS0101+ NHSIL12+ M7824 will be evaluated in all HPV serotypes given the clinical remissions PDS Biotechnology has observed in CIN studies with PDS0101 in non-HPV 16 serotypes. However, if it is found that the ORR with non-HPV-16 serotypes is noticeably less than the ORR observed with HPV 16 serotypes than patients having disease related to non-HPV-16 serotypes may be replaced with patients having disease related to the HPV-16 serotype for the first and second stage of the primary efficacy analyses.
- In addition, while the primary focus of this protocol is to evaluate the response rate of this I/O combination in patients with a wide array of HPV associated malignancies, if the I/O combination in not deemed to be promising in this larger population (less than three out of eight evaluable patients have a response) than the study will be amended to evaluate this I/O combination specifically in patients with cervical cancer (and patients who have already been enrolled with non-cervical cancers may be replaced with cervical cancer patients for the first stage of the primary efficacy analyses).

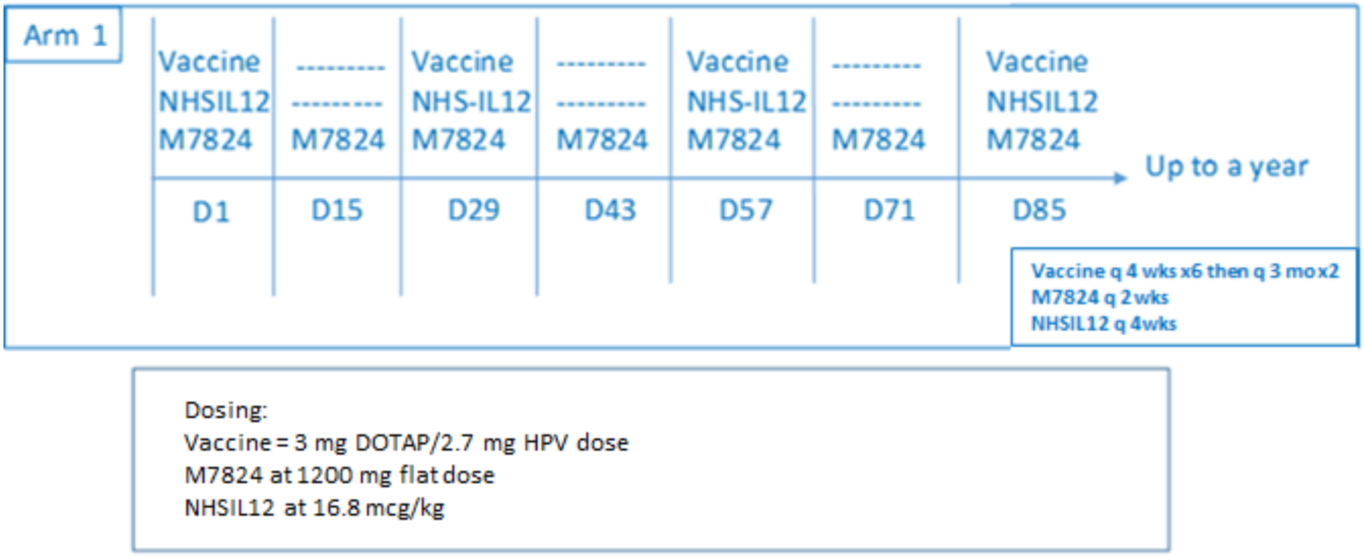

Figure 19.

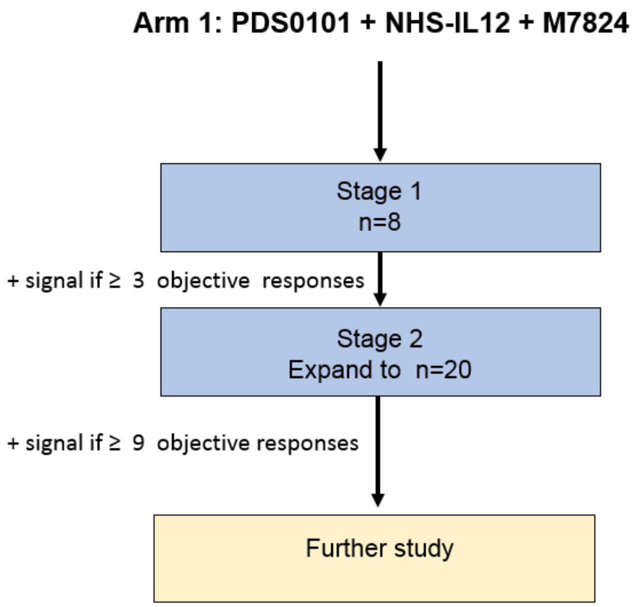

Figure 20.

3.1.1 Dose Limiting Toxicity

DLT for first 6 patients:

Dose-limiting toxicity (DLT) will be defined as any one of the following adverse events, possibly attributable to study drugs, that occur within 28 days of the start of therapy:

- Any Grade 4 or higher adverse drug reactions (ADRs) as defined by CTCAE v5.0 and assessed as possibly related to that agent by the Investigator, except for laboratory values that are asymptomatic or resolve to Grade ≤ 1 or baseline grade within 7 days without medical intervention..
- Any Grade 3 ADRs possibly attributed to an agent except for any of the following:
  - Grade 3 flu-like symptoms or fever, as well as associated symptoms of fatigue, headaches, nausea, emesis which can be controlled with conservative medical management.
  - Tumor flare phenomenon defined as local pain, irritation, or rash localized at sites of known or suspected tumor.
  - Grade 3 Hgb decrease (< 8.0 g/dL) that is clinically manageable with blood transfusions or erythroid growth factor use does not require treatment discontinuation.
  - Grade 3 laboratory values that are asymptomatic or resolve to Grade ≤ 1 or baseline grade within 7 days without medical intervention.
  - Keratoacanthoma and squamous cell carcinoma of the skin.
  - Any endocrinopathy that can be medically managed with hormone replacement
  - Any grade 3 adverse drug reaction which can be medically managed with minimal risk to the patient (e.g. placement of a pleural catheter for recurrent inflammatory pleural effusions) and resolves to at least grade one or baseline grade within 72 hours.

Criteria are based on the NCI Common Terminology Criteria for Adverse Events, Version 5.

Where clinically appropriate subjects will receive one year of treatment during which subjects will be followed with surveillance scans every 6-12 weeks. On a case by case basis treatment beyond a year is allowed per investigator discretion. Subjects with evidence of disease progression after completing a year of treatment will be allowed retreatment.

Patents might be taken off treatment for disease progression prior to completing one year of treatment, but where clinically appropriate, treatment beyond radiographic progression is allowed if in the opinion of the investigator the subject is benefiting from treatment.

Subjects will be taken off treatment if unacceptable toxicity occurs and is attributed to all therapeutic agents. If a single agent is tolerated that agent may be continued.

This trial will be conducted using a Simon two-stage phase II trial design in which 8 evaluable patients will enroll and if 3 or more of the 8 have an objective response, then accrual would continue until a total of 20 evaluable patients have been treated. If there are 9 or more of 20 (45.0%) who experience a response, this would be sufficiently interesting to warrant further study.

In the event that a patient develops a response (>30% decrease in target lesions as compared with baseline scan) following initial progression or “pseudo-progression”, this response will be considered as an objective response in the determination to expand accrual.

3.2 DRUG ADMINISTRATION

General Rule:

A window of +/- 14 days for a scheduled treatment is allowed in the event of scheduling issues (i.e., holiday, bad weather or other scheduling issues). The minimum time between administrations of a similar agent is 11 days. Where feasible PDS0101 should be administered first, followed by NHS-IL12 (when given), followed by M7824.

PDS0101

PDS0101 will be administered every 4 weeks for 6 doses followed by every three months (i.e. every 12 weeks) for an additional two doses.

Dosage Form:	Sterile injectable liquid
Number of vials:	2-vial formulation
Preparation:	1:1 v/v mixture of the 2 vial formulation. 0.6mL of vial #1, ImmunoMAPK-RDOTAP added to vial# 2, HPVmix (0.6 mL)
Total Administered dose:	2.7 mg of total peptide and 3 mg of R-DOTAP
Total Injection Volume:	1.0mL (administered as two individual 0.5mL injections)
Injection route:	Subcutaneous

- 0.6 ml will be drawn up from each vial (Vial #1 Immuno MAPK-RDOTAP, Vial #2 HPV mix) and mixed (total = 1.2ml). Two separate 0.5 ml doses will be injected into the patient. These can be given in separate limbs or preferably ~ 6cm apart in the same limb.

M7824

Subjects will receive M7824 via IV infusion over 1 hour (-10 minutes / +20 minutes, that is, over 50 to 80 minutes) once every 2 weeks. M7824 will be administered as a "flat" dose of 1,200 mg independent of body weight. M7824 is administered as an intravenous infusion with a mandatory 0.2 micron in-line filter.

In order to mitigate potential infusion-related reactions, premedication with an antihistamine and with acetaminophen (for example, 25-50 mg diphenhydramine and 500-650 mg acetaminophen) within approximately 30 to 60 minutes prior to dosing of M7824 is optional and at the discretion of the Investigator.

NHS-IL12

NHS-IL12 will be administered at as dose of 16.8 μg/kg by SC injection every 4 weeks. The dose of NHS-IL12 will be calculated based on the weight of the subject determined within 72 hours prior to the day of drug administration. The dose of NHS-IL12 used for the previous

administration can be repeated if the change in the subject's weight is 10% or less than the weight used for the last dose calculation.

3.3 STUDY INTERVENTION COMPLIANCE

All study interventions will be given at NIH and documented in the electronic medical record.

3.4 DOSE MODIFICATIONS

Discontinuation

Treatment with individual agents will be discontinued in the case of:

- Any Grade 4 or higher adverse drug reactions (ADRs) as defined by CTCAE v5.0 and assessed as possibly related to that agent by the Investigator, except for laboratory values that are asymptomatic or resolve to Grade ≤ 1 or baseline grade within 7 days without medical intervention".
- Any Grade 3 ADRs possibly attributed to an agent except for any of the following:
    - Grade 3 flu-like symptoms or fever, as well as associated symptoms of fatigue, headaches, nausea, emesis which can be controlled with conservative medical management.
    - Tumor flare phenomenon defined as local pain, irritation, or rash localized at sites of known or suspected tumor.
    - Grade 3 Hgb decrease (< 8.0 g/dL) that is clinically manageable with blood transfusions or erythroid growth factor use does not require treatment discontinuation.
    - Grade 3 laboratory values that are asymptomatic or resolve to Grade ≤ 1 or baseline grade within 7 days without medical intervention.
    - Keratoacanthoma and squamous cell carcinoma of the skin.
    - Any endocrinopathy that can be medically managed with hormone replacement
    - Any grade 3 adverse drug reaction which can be medically managed with minimal risk to the patient (e.g., placement of a pleural catheter for recurrent inflammatory pleural effusions) and resolves to at least grade one or baseline grade within 72 hours.

Dose Delay

Individual agents should be withheld for any Grade 2 or 3 ADR possibly attributed to that agent until resolution to Grade ≤ 1 unless the ADR in the opinion of the investigator is not clinically relevant or can be medically managed with minimal risk to the patient. Should a clinically relevant grade 2 or 3 ADR persist for more than 4 weeks, consideration should be given to discontinuing treatment with that individual agent at the discretion of the investigator.

For non-medical logistical reasons, unrelated acute illnesses, or palliative radiation, scheduled assessments and dosing can be delayed up to 2 months. Where at all possible, dosing should be restarted to keep in line with the original treatment schedule.

Dose Modification

During DLT period dose modifications are not allowed.

HPV vaccine: Vaccine doses will not be modified but doses may be skipped per investigator discretion.

M7824: Dose reductions are not allowed but doses may be skipped per investigator discretion.

NHS-IL12: Dose reductions to 8 or 12 mcg/kg for toxicities attributed to NHS-IL12 will be allowed at the discretion of the investigator.

Stopping Rule: Any death at least possibly related to study regimen will prompt a suspension of accrual and reassessment of available safety information prior to reopening study to accrual.

Guidelines for Management of Infusion Reactions

**NCI-CTCAE Grade**	**Treatment Modification for Infused Agent (M7824)**
**Grade 1 – mild**<br>Mild transient reaction; infusion interruption not indicated; intervention not indicated.	Consider decreasing the infusion rate of the particular agent by 50% and monitoring closely for any worsening.
**Grade 2 – moderate**<br>Therapy or infusion interruption indicated but responds promptly to symptomatic treatment (e.g., antihistamines, NSAIDs, narcotics, i.v. fluids); prophylactic medications indicated for ≤ 24 hours.	Consider temporarily discontinuing infusion of the particular agent.<br>Consider resuming infusion of the particular agent at 50% of previous rate once infusion related reaction has resolved or decreased to at least Grade 1 in severity, and monitor closely for any worsening.
**Grade 3 or Grade 4 – severe or life-threatening**<br>*Grade 3:* Prolonged (e.g., not rapidly responsive to symptomatic medication and/or brief interruption of infusion); hospitalization indicated for clinical sequelae.<br>*Grade 4*: Life-threatening consequences; urgent intervention indicated.	Stop the infusion immediately and disconnect infusion tubing from the subject.<br>For grade 3 events: Consider withdrawing immediately from treatment with that particular agent and not offering any further treatment with that agent based upon if the clinical condition can be safely managed.<br>For grade 4 events: Withdraw immediately from treatment and do not offer further treatment with that agent.

If the infusion rate of M7824 has been decreased by 50% or interrupted due to an infusion reaction, keep it decreased for the next scheduled infusion. If no infusion reaction is observed in the next scheduled infusion, the infusion rate may be returned to baseline at the subsequent infusions based on investigator's medical judgment.

If hypersensitivity reaction occurs, the subject should be treated according to the best available medical practice.

Guidelines for Management of Immune-Mediated Adverse Reactions

(In addition to the below guidelines, investigators may also use NCCN, ASCO, SITC or FDA guidelines for Immune Related Adverse Events)

**Gastrointestinal irAEs**		
**Severity of Diarrhea/Colitis (NCI-CTCAE v5)**	**Initial Management**	**Follow-up Management**
**Grade 1**<br>Diarrhea: < 4 stools/day over Baseline<br>Colitis: asymptomatic	Consider continuing M7824<br>Symptomatic treatment (e.g. loperamide)	Consider close monitoring for worsening symptoms<br>Consider educating subject to report worsening immediately<br>If worsens:<br>Treat as Grade 2, 3 or 4.
**Grade 2**<br>Diarrhea: 4 to 6 stools per day over Baseline; IV fluids indicated < 24 hours; not interfering with ADL<br>Colitis: abdominal pain; blood in stool	Consider withholding M7824<br>Symptomatic treatment	If improves to Grade ≤ 1:<br>Consider resuming therapy<br><br>If persists > 7 days or recurs:<br>Consider treating as Grade 3 or 4.
**Grade 3 to 4**<br>Diarrhea (Grade 3): ≥ 7 stools per day over Baseline; incontinence; IV fluids ≥ 24 h; interfering with ADL<br>Colitis (Grade 3): severe abdominal pain, medical intervention indicated, peritoneal signs<br>Grade 4: life-threatening, perforation	Consider withholding or permanently discontinuing M7824 for Grade 3 events based upon if the clinical condition can be safely managed. For grade 4 events permanently discontinue treatment.<br><br>Consider 1.0 to 2.0 mg/kg/day prednisone IV or equivalent<br><br>Consider adding prophylactic antibiotics for opportunistic infections<br><br>Consider lower endoscopy	If improves:<br>Consider continuing steroids until Grade ≤ 1, then tapering over at least 1 month; consider resuming therapy following steroids taper (for initial Grade 3).<br><br>If worsens, persists > 7 days, or recurs after improvement:<br>Consider adding infliximab 5mg/kg (if no contraindication).<br>Note: infliximab should not be used in cases of perforation or sepsis.

Dermatological irAEs		
**Grade of Rash (NCI-CTCAE v5)**	**Initial Management**	**Follow-up Management**
**Grade 1 to 2**<br>Covering ≤ 30% body surface area	Consider continuing M7824<br><br>Consider symptomatic therapy (for example, antihistamines, topical steroids)	If persists > 1 to 2 weeks or recurs:<br>Consider withholding M7824 therapy<br>Consider skin biopsy<br><br>Consider 0.5-1.0 mg/kg/day prednisone or equivalent. Once improving, consider tapering steroids over at least 1 month, consider prophylactic antibiotics for opportunistic infections, and consider resuming M7824 therapy following steroids taper.<br>If worsens:<br>Consider treating as Grade 3 to 4.
**Grade 3 to 4**<br>Grade 3: Covering > 30% body surface area;<br>Grade 4: Life threatening consequences	Consider withholding or permanently discontinuing M7824 for Grade 3 events based upon if the clinical condition can be safely managed. For grade 4 events permanently discontinue treatment.<br>Consider skin biopsy<br>Consider dermatology consult<br>Consider 1.0 to 2.0 mg/kg/day prednisone or equivalent<br><br>Consider adding prophylactic antibiotics for opportunistic infections	If improves to Grade ≤ 1:<br>Consider tapering steroids over at least 1 month; consider resuming therapy following steroids taper (for initial Grade 3).
**Pulmonary irAEs**		
**Grade of Pneumonitis (NCI-CTCAE v5)**	**Initial Management**	**Follow-up Management**
**Grade 1**<br>Radiographic changes only	Consider withholding M7824<br>Consider Pulmonary and Infectious Disease consults	Consider re-assessing at least every 3 weeks<br>If worsens:<br>Consider treating as Grade 2 or Grade 3 to 4.
**Grade 2**<br>Mild to moderate new symptoms	Consider withholding M7824	If improves:

	Consider pulmonary and Infectious Disease consults<br>Consider monitoring symptoms daily; consider hospitalization<br>Consider 1.0 to 2.0 mg/kg/day prednisone or equivalent<br>Consider adding prophylactic antibiotics for opportunistic infections<br>Consider bronchoscopy, lung biopsy	When symptoms return to Grade ≤ 1, consider tapering steroids over at least 1 month, and then consider resuming therapy following steroids taper<br>If not improving after 2 weeks or worsening:<br>Consider treating as Grade 3 to 4.
**Grade 3 to 4**<br>Grade 3: Severe new symptoms; New/worsening hypoxia;<br>Grade 4: Life-threatening	Consider withholding or permanently discontinuing M7824 for Grade 3 events based upon if the clinical condition can be safely managed. For grade 4 events permanently discontinue treatment.<br>Consider hospitalization.<br>Consider pulmonary and Infectious Disease consults.<br>Consider 1.0 to 2.0 mg/kg/day prednisone or equivalent<br>Consider adding prophylactic antibiotics for opportunistic infections<br>Consider bronchoscopy, lung biopsy	If improves to Grade ≤ 1:<br>Consider tapering steroids over at least 1 month<br>If not improving after 48 hours or worsening:<br>Consider adding additional immunosuppression (for example, infliximab, cyclophosphamide, IV immunoglobulin, or mycophenolate mofetil)
**Hepatic irAEs**		
**Grade of Liver Test Elevation (NCI-CTCAE v5)**	**Initial Management**	**Follow-up Management**
**Grade 1**<br>Grade 1 AST or ALT > ULN to 3.0 x ULN and/or Total bilirubin > ULN to 1.5 x ULN	Consider continuing M7824	Consider continued liver function monitoring<br>If worsens:<br>Consider treating as Grade 2 or 3 to 4.
**Grade 2**<br>AST or ALT > 3.0 to ≤ 5 x ULN and/or total bilirubin > 1.5 to ≤ 3 x ULN	Consider withholding M7824	If returns to Grade ≤ 1:<br>Consider resuming therapy.<br>If elevation persists > 7 days or worsens:<br>Consider treating as Grade 3 to 4.
**Grade 3 to 4**<br>AST or ALT > 5 x ULN and/or total bilirubin > 3 x ULN	Consider withholding or permanently discontinuing M7824 for Grade 3 events based upon if the clinical condition can be safely managed. For grade 4 events permanently discontinue treatment.	If returns to Grade ≤ 1:<br>Consider tapering steroids over at least 1 month<br>If does not improve in > 7 days, worsens or rebounds:

	Consider 1.0 to 2.0 mg/kg/day prednisone or equivalent<br>Consider adding prophylactic antibiotics for opportunistic infections<br>Consider consulting gastroenterologist/ hepatologist<br>Consider obtaining MRI/CT scan of liver and liver biopsy if clinically warranted	Consider adding mycophenolate mofetil 1 gram (g) twice daily<br>If no response within an additional 7 days, consider other immunosuppressants per local guidelines.
**Renal irAEs**		
**Grade of Creatinine Increased (NCI-CTCAE v5)**	**Initial Management**	**Follow-up Management**
**Grade 1**<br>Creatinine increased > ULN to 1.5 x ULN	Consider continuing M7824	Continue renal function monitoring<br>If worsens:<br>Consider treating as Grade 2 to 3 or 4.
**Grade 2**<br>Creatinine increased > 1.5 and ≤ 6 x ULN	Consider withholding M7824<br>Consider 1.0 to 2.0 mg/kg/day prednisone or equivalent.<br>Consider adding prophylactic antibiotics for opportunistic infections<br>Consider renal biopsy	If returns to Grade ≤1:<br>Consider tapering steroids over at least 1 month, and consider resuming therapy following steroids taper.<br>If worsens:<br>Treat as Grade 4.
**Grade 3-4**<br>Creatinine increased > 6 x ULN	Consider withholding or permanently discontinuing M7824 for Grade 3 events based upon if the clinical condition can be safely managed. For grade 4 events permanently discontinue treatment.<br>Consider 1.0 to 2.0 mg/kg/day prednisone or equivalent.<br>Consider adding prophylactic antibiotics for opportunistic infections<br>Consider renal biopsy<br>Consider Nephrology consult	If returns to Grade ≤1:<br>Consider tapering steroids over at least 1 month.

Cardiac irAEs		
**Myocarditis**	**Initial Management**	**Follow-up Management**
New onset of cardiac signs or symptoms and / or new laboratory cardiac biomarker elevations (e.g. troponin, CK-MB, BNP) or cardiac imaging abnormalities suggestive of myocarditis.	Consider withholding or permanently discontinuing M7824 based upon if the clinical condition can be safely managed.<br>Consider hospitalization<br>In the presence of life threatening cardiac decompensation, consider transfer to a facility experienced in advanced heart failure and arrhythmia management.<br>Consider cardiology consult to establish etiology and rule-out immune-mediated myocarditis.<br>Consider myocardial biopsy if recommended per cardiology consult.	If symptoms improve and immune-mediated etiology is ruled out, consider re-starting therapy.<br><br>If symptoms do not improve/worsen, viral myocarditis is excluded, and immune-mediated etiology is suspected or confirmed following cardiology consult, consider managing as immune-mediated myocarditis.
Immune-mediated myocarditis	Consider withholding or permanently discontinuing M7824 based upon if the clinical condition can be safely managed.<br>Consider guideline based supportive treatment as appropriate as per cardiology consult.<br>Consider 1.0 to 2.0 mg/kg/day prednisone or equivalent<br>Consider adding prophylactic antibiotics for opportunistic infections.	Once improving, consider tapering steroids over at least 1 month.<br><br>If no improvement or worsening, consider additional immunosuppressants (e.g. azathioprine, cyclosporine A).
*Local guidelines, or eg. ESC or AHA guidelines<br>ESC guidelines website: https://www.escardio.org/Guidelines/Clinical-Practice-Guidelines<br>AHA guidelines website:<br>http://professional.heart.org/professional/GuidelinesStatements/searchresults.jsp?q=&y=&t=1001		
**Endocrine irAEs**		
**Endocrine Disorder**	**Initial Management**	**Follow-up Management**
**Grade 1 or Grade 2 endocrinopathies (hypothyroidism, hyperthyroidism, adrenal insufficiency, type I diabetes mellitus)**	Consider continuing M7824<br>Consider endocrinology consult if needed	Consider continuing hormone replacement/suppression and monitoring of endocrine function as appropriate.

	Consider starting thyroid hormone replacement therapy (for hypothyroidism), anti-thyroid treatment (for hyperthyroidism), corticosteroids (for adrenal insufficiency) or insulin (for Type I diabetes mellitus) as appropriate.<br><br>Consider ruling-out secondary endocrinopathies (i.e. hypopituitarism / hypophysitis)	
**Grade 3 or Grade 4 endocrinopathies (hypothyroidism, hyperthyroidism, adrenal insufficiency, type I diabetes mellitus)**	Consider withholding or permanently discontinuing M7824 for Grade 3-4 events based upon if the clinical condition can be safely managed.<br>Consider hospitalization<br>Consider endocrinology consult<br><br>Consider starting thyroid hormone replacement therapy (for hypothyroidism), anti-thyroid treatment (for hyperthyroidism), corticosteroids (for adrenal insufficiency) or insulin (for type I diabetes mellitus) as appropriate.<br><br>Consider ruling-out secondary endocrinopathies (i.e. hypopituitarism / hypophysitis)	Consider resuming therapy once symptoms and/or laboratory tests improve to Grade ≤ 1 (with or without hormone replacement/suppression).<br><br>Consider continuing hormone replacement/suppression and monitoring of endocrine function as appropriate.
**Hypopituitarism/Hypophysitis (secondary endocrinopathies)**	If secondary thyroid and/or adrenal insufficiency is confirmed (i.e. subnormal serum FT4 with inappropriately low TSH and/or low serum cortisol with inappropriately low ACTH):<br>• Consider referring to endocrinologist for dynamic testing as indicated and measurement of other hormones (FSH, LH, GH/IGF-1, PRL, testosterone in men, estrogens in women)<br>• Consider hormone replacement/suppressive therapy as appropriate<br>• Consider performing pituitary MRI and visual field examination as indicated<br><br>**If hypophysitis confirmed:**	Consider resuming therapy once symptoms and hormone tests improve to Grade ≤ 1 (with or without hormone replacement).<br><br>In addition, for hypophysitis with abnormal MRI, consider resuming M7824 only once shrinkage of the pituitary gland on MRI/CT scan is documented.<br><br>Consider continuing hormone replacement/suppression therapy as appropriate.

	• Continue M7824 if mild symptoms<br>• Consider withholding M7824 and if moderate, severe or life-threatening symptoms of hypophysitis. Consider hospitalization. Consider initiating corticosteroids (1 to 2 mg/kg/day prednisone or equivalent) followed by corticosteroids taper during at least 1 month.<br>• Consider adding prophylactic antibiotics for opportunistic infections.	
**Other irAEs (not described above)**		
**Grade of other irAEs (NCI-CTCAE v5)**	**Initial Management**	**Follow-up Management**
**Grade 2 or Grade 3 clinical signs or symptoms suggestive of a potential irAE**	Consider withholding M7824 pending clinical investigation	If irAE is ruled out, consider managing as appropriate according to the diagnosis and consider re-starting therapy<br>If irAE is confirmed, consider treating as Grade 2 or 3 irAE.
**Grade 2 irAE or first occurrence of Grade 3 irAE**	Consider withholding M7824 based upon if the clinical condition can be safely managed.<br>Consider 1.0 to 2.0 mg/kg/day prednisone or equivalent<br>Consider adding prophylactic antibiotics for opportunistic infections<br>Specialty consult as appropriate	If improves to Grade ≤ 1:<br>Consider tapering steroids over at least 1 month and resuming therapy following steroids taper.
**Recurrence of same Grade 3 irAEs**	Consider permanently discontinuing M7824 based upon if the clinical condition can be safely managed;<br>Consider<br>1.0 to 2.0 mg/kg/day<br>prednisone or equivalent<br>Consider adding prophylactic antibiotics for opportunistic infections<br>Specialty consult as appropriate	If improves to Grade ≤ 1:<br>Consider tapering steroids over at least 1 month.

**Grade 4**	Permanently discontinue M7824<br>Consider to 2.0 mg/kg/day prednisone or equivalent and/or other immunosuppressant as needed<br>Consider adding prophylactic antibiotics for opportunistic infections<br>Consider specialty consult as appropriate	If improves to Grade ≤ 1:<br>Consider tapering steroids over at least 1 month
**Requirement for 10 mg per day or greater prednisone or equivalent for more than 12 weeks for reasons other than hormonal replacement for adrenal insufficiency**<br><br>**Persistent Grade 2 or 3 irAE lasting 12 weeks or longer**	Consider permanently discontinuing M7824<br>Consider specialty consult as appropriate	

Abbreviations: ACTH=adrenocorticotropic hormone; ADL=activities of daily living; ALT=alanine aminotransferase; AST=aspartate aminotransferase; BNP=B-type natriuretic peptide; CK-MB=creatine kinase MB; CT= computed tomography; FSH=follicle-stimulating hormone; GH=growth hormone; IGF-1=insulin-like growth factor 1; irAE=immune related adverse event; IV=intravenous; LH=luteinizing hormone; MRI=magnetic resonance imaging; NCI CTCAE=National Cancer Institute Common Terminology Criteria for Adverse Events; PRL=prolactin;T4=thyroxine; TSH=thyroid stimulating hormone; ULN=upper limit of normal

3.5 STUDY CALENDAR

Procedure	Screening [1]	Baseline / Week 1[2]	Week (2N+1)[3]	EOT[4]	Safety follow up[5]	Long Term follow up[6]
Treatment[3]		X	X			
NIH Advance Directives Form[7]		X				
Medical History	X					
Height	X					
Physical exam, weight, vital signs, ECOG	X	X	X	X	X	
HIV, HCV, HepB	X					
EKG	X			X	X	
CBC with differential	X	X	X	X	X	
Biochemical profile[8]	X	X	X	X	X	
Tumor Markers[14]		X	X	X		
CD4 [11]	X					
ACTH, TSH, Free T4, lipase, amylase, CRP		X	X[9]			
Urinalysis	X					
Urine or serum pregnancy testing in women of childbearing potential	X	X	X			
PT, INR, aPTT		X				
Tumor evaluation (CT Scan / MRI)[10]	X		X			X
Brain CT/MRI [11]	X					
Nuclear bone scan [11]	X					
Concomitant Medications		X	X	X	X	
Adverse events		X	X	X	X	
Optional biopsy for immune analysis [12]		X	X			

**Procedure**	**Screening** 1	**Baseline / Week 1**[2]	**Week (2N+1)**[3]	**EOT**[4]	**Safety follow up**[5]	**Long Term follow up**[6]
Research Blood [13]		X	X	X		
Telephone Follow Up					X	X

1. Screening evaluations performed within 28 days prior to the first drug administration, unless specified in Section **2.2.**

2. Baseline evaluations performed within 1 week prior to first drug administration.

3. Schedule of therapeutic agents administered will be as outlined in Section **3.1**, **Figure 19.**. NHS-IL12 will be given every 4 weeks, M7824 infusion will be every two weeks and HPV vaccine will be given every 4 weeks for the first 6 doses followed by every 12 weeks thereafter. (The first administration of HPV vaccine should preferably be given 1-2 weeks before the first administration of NHS-IL12 and M7824.) Administration of study agents and indicated evaluations can be done up to 14 days earlier or delayed up to 14 days due to holidays, inclement weather, conflicts, or similar reasons. The timing of subsequent administrations is adjusted to maintain a minimum of 11 days between treatments of similar agents.

4. EOT – End of treatment visit: Where feasible, on the day of or within 7 days of the decision to discontinue treatment prematurely before completion of one year of treatment. Does not need to be completed if drug is withheld after one year of treatment.

5. 28 days (+/- 7 days) after last treatment. If subjects are not willing to come to NIH to FU visit, they will be contacted by phone to assess adverse events.

6. Patients who have come off treatment for disease progression will be followed by phone annually for survival. Patients who have not progressed on treatment will continue to be followed and scanned per investigator discretion until progression. Those that completed one year of treatment will be invited for an additional year of treatment at the time of progression. Initial and follow up courses of treatment may extend beyond a year per investigator discretion.

7. As indicated in Section **12.3**, all subjects will be offered the opportunity to complete an NIH advanced directives form. This should be done preferably at baseline but can be done at any time during the study as long as the capacity to do so is retained. The completion of the form is strongly recommended, but is not required

8. Biochemical profile: sodium, potassium, chloride, bicarbonate, calcium, glucose, BUN, creatinine, ALT, AST, alkaline phosphatase, total protein, albumin, and total and direct bilirubin

9. Every 8 weeks.

10. Every 8 weeks. In the event of a PR or CR tumor imaging assessments may be performed every 3 months (+/- 2 weeks) at the discretion of the investigator. Tumor assessment should be continued beyond end of treatment in patients who have not experienced PD until they experience PD in order to assess PFS.

11. If clinically indicated

12. Optional biopsies at baseline and as close as possible to first imaging restaging.

13. Where feasible, research blood for study assessments (see Section **5**) will be collected at baseline, 3 days and 1 week after the first M7824 + NHS-IL12 dose) as well as at weeks 3 and 7 and at restaging visits in selected patients, per PI discretion.

14. Evaluate CEA, CA19-9, CA125 and CA15-3 at baseline and follow elevated values thereafter

3.6 Cost and Compensation

3.6.1 Costs

NIH does not bill health insurance companies or participants for any research or related clinical care that participants receive at the NIH Clinical Center. If some tests and procedures performed outside the NIH Clinical Center, participants may have to pay for these costs if they are not covered by insurance company. Medicines that are not part of the study treatment will not be provided or paid for by the NIH Clinical Center.

3.6.2 Compensation

There will be no compensation provided on this study as per informed consent.

3.6.3 Reimbursement

This study offers subject reimbursement or payment for travel, lodging and/or meals while participating in the research. The amount, if any, is guided by NIH policies and guidelines.

On this study, the NCI will cover the cost for some of the expenses. Some of the costs may be paid directly by the NIH and some may be reimbursed to the subject. Someone will work with subjects to provide more information.

3.7 Criteria for Removal from Protocol Therapy and Off Study Criteria

Regardless of reason for removal from study therapy, patients will be asked to have a 28 day follow up safety visit. Patients who refuse to return for this visit will be asked to review any safety concerns by phone within this time period.

3.7.1 Criteria for Removal from Protocol Therapy

- Clinical or radiographic progression of disease except when the investigator feels the subject is still benefiting from treatment. (It is generally preferable for patients to remain on treatment past initial radiographic progression in case there is pseudo -progression, except when the investigator feels that the clinical picture warrants changing therapy at initial progression).
- Unacceptable Toxicity possibly attributed to all active therapies.
- Participant requests to be withdrawn from active therapy.
- Start of another systemic anticancer treatment or participation in another investigational therapeutic trial. Focal palliative radiotherapy, ablation, or surgery to a site of disease will not necessitate removal from protocol therapy.
- Investigator discretion.
- Positive pregnancy test.

3.7.2 Off-Study Criteria

- Screen failure.
- PI decision to end the study.

- Participant requests to be withdrawn from study.
- Participant lost to follow up.
- Investigator discretion.
- Death.

3.7.3 Lost to Follow-up

A participant will be considered lost to follow-up if he or she fails to return for 4 scheduled visits and is unable to be contacted by the study site staff.

The following actions must be taken if a participant fails to return to the clinic for a required study visit:

- The site will attempt to contact the participant and reschedule the missed visit for within the next 2 weeks and counsel the participant on the importance of maintaining the assigned visit schedule and ascertain if the participant wishes to and/or should continue in the study.
- Before a participant is deemed lost to follow-up, the investigator or designee will make every effort to regain contact with the participant (where possible, 3 telephone calls and, if necessary, a certified letter to the participant's last known mailing address or local equivalent methods). These contact attempts should be documented in the participant's medical record or study file.
- Should the participant continue to be unreachable, he or she will be considered to have withdrawn from the study with a primary reason of lost to follow-up.

3.7.4 Off Protocol Therapy and Off-Study Procedure

Authorized staff must notify Central Registration Office (CRO) when a subject is taken off protocol therapy and when a subject is taken off-study. A Participant Status Update Form from the website (http://home.ccr.cancer.gov/intra/eligibility/welcome.htm) main page must be completed and sent via encrypted email to: NCI Central Registration Office [redacted]

4 CONCOMITANT MEDICATIONS/MEASURES

Any medications (other than those excluded by the clinical trial protocol) that are considered necessary to protect subject welfare or alleviate symptoms and will not interfere with the trial medication may be given at the Investigator's discretion.

Palliative radiotherapy delivered in a normal organ-spearing technique may be administered during the trial. The assessment of PD will not be based on the necessity for palliative radiotherapy.

4.1 THE FOLLOWING TREATMENTS SHOULD NOT BE ADMINISTERED DURING THE TRIAL

- Other immunotherapies or immunosuppressive drugs (for example, chemotherapy or systemic corticosteroids except for prophylaxis or treatment of allergic reactions, endocrine replacement therapy at low dose prednisone [≤ 10 mg daily] or equivalent, for the treatment of irAEs, or for short courses (≤ 14 days) as appropriate medical therapy for

unrelated medical conditions (e.g., asthma). Steroids with no or minimal systemic effect (topical, inhalation) are allowed.

- Prophylactic use of corticosteroids for infusion related reactions. Corticosteroid administration prior to CT scans in patients with intravenous contrast allergy is allowed.
- Any live vaccine therapies for the prevention of infectious disease. Administration of inactivated vaccines is allowed (for example, inactivated influenza vaccines)
- Systemic anticancer treatment.

5 BIOSPECIMEN COLLECTION

5.1 Correlative Studies for Research/Pharmacokinetic Studies

Test/assay	Volume (approx.) per Timepoint	Type of tube	Collection point	Location of specimen analysis*
Standard and 123 immune cell subsets by FACS	60-80 mL blood for PBMCs	Sodium heparin (green top) tubes	See study calendar 3.5	LTIB
Functional Analysis of immune cell subsets by FACS				LTIB
Antigen Specific Immune Response by cytokine staining assay				LTIB
T cell clonality by immunseq platform				LTIB and NCI Frederick Genomic Core Facility
RNA expression level of 770 genes				LTIB and NCI Frederick Genomic Core Facility
Soluble Factors (to include sCD27 and sCD40 ligand) by ELISA	16 mL blood for serum	SST		LTIB

Test/assay	Volume (approx.) per Timepoint	Type of tube	Collection point	Location of specimen analysis*
Immune Markers by IHC	Tumor samples	N/A		Dr. Houssein Sater's Lab
RNA expression level of 770 genes	Tumor samples	N/A		LTIB and NCI Frederick Genomic Core Facility
HPV status by PCR of DNA	Tumor samples	N/A		Dr. Hinrich's Lab
Circulating tumor DNA (ctDNA) by PCR system	Plasma	EDTA (lavender top)		Dr. Liang Cao Lab
T cell clonality by immunoseq platform	Tumor samples	N/A		LTIB and NCI Frederick Genomic Core Facility

*Research blood samples will be sent to the Clinical Services Program – Leidos Biomedical Research, Inc. (CSP) (Section 5.3.1) for barcoding, initial processing and storage. Tissue will be sent to the Laboratory of Pathology as described in Section 5.3.2. From these facilities, coded, linked samples will be sent to the designated labs for analysis upon request.

5.1.1 Immune Phenotyping

Exploratory immunologic studies will be conducted to evaluate the study drug's effect on the immune response before and after treatment, to gain insight into potential biomarkers, and help improve the administered therapy. Blood will be collected as per Study Calendar 3.5. The following immune assays may be performed at the Laboratory of Tumor Immunology and Biology (LTIB) at the NCI's Center for Cancer Research (CCR) in select patients where adequate samples are available:

1. PBMCs may be analyzed for changes in standard immune cell types (CD4 and CD8 T cells, natural killer [NK] cells, regulatory T cells [Tregs], myeloid-derived suppressor cells [MDSCs], and dendritic cells) as well as 123 immune cell subsets, using multi-color flow cytometry.

2. PBMCs from selected subjects may be analyzed for function of specific immune cell subsets, including CD4 and CD8 T cells, NK cells, Tregs, and MDSCs using flow-based assays.

3. PBMCs may be analyzed for tumor antigen-specific immune responses using an intracellular cytokine staining assay. PBMCs will be stimulated in vitro with overlapping 15-mer peptide pools encoding the tumor-associated antigens such as CEA, MUC-1 and Brachyury; control peptide

pools will involve the use of human leukocyte antigen peptide as a negative control and CEFT peptide mix as a positive control. CEFT is a mixture of peptides of CMV, Epstein-Barr virus, influenza, and tetanus toxin. Post-stimulation analyses of CD4 and CD8 T cells will involve the production of IFN-γ, IL-2, TNF, and the degranulation marker CD107a. If sufficient PBMCs are available, assays may also be performed for the development of T cells to other tumor-associated antigens.

5.1.2 Soluble Factors

Samples for soluble factor analysis will be collected as per Study Calendar **3.5**.

-Sera may be analyzed pre- and post-therapy for the following soluble factors: sCD27, sCD40 ligand using commercial ELISA kits.

-Sera may be analyzed for changes in cytokines (IFN-γ, IL-10, IL-12, IL-2, IL-4, etc.), chemokines, antibodies, tumor-associated antigens, and/or other markers using ELISA or multiplexed assays (e.g., Mesoscale, Luminex, cytokine bead array).

5.1.3 Analyses of Tumor Tissue for Immune Markers

Study of immune infiltration as well as PD-L1 status within the tumor microenvironment pre vs. post treatment by IHC and/or multiplex immunofluorescence may be performed by Dr. Houssein Sater (NCI, GMB). Where available, archival tumor samples will be requested (preferably tissue samples from the last 6 months). For patients with lesions amenable to biopsy, two optional biopsies may be performed at baseline and at first restaging. Tumor samples will be sent to the Laboratory of Pathology for disease evaluation; remaining samples will be used for research. Tissue samples for research may also be stored in CSP as described in Section **5.3.1**.

5.1.4 Assessment of HPV status

In patients with available tumor tissue (either archival or by optional biopsy), HPV testing will be performed using the Roche Cobas or Becton Dickinson HPV PCR based DNA assay, if no prior HPV testing of the tumor has been performed. This will be done through collaboration with Dr. Hinrich's Lab.

5.1.5 Circulating tumor DNA

If sufficient plasma is available, selected patient samples may be analyzed for circulating tumor DNA (Collaboration with Dr. Liang Cao). Plasma DNA will be isolated with an automated purification system by Promega Corp using a system-attached bar-code reader to track samples and DNA products. The circulating tumor/HPV DNA will be quantified with a digital droplet PCR system from Bio-Rad to obtain precise quantification. This is just quantification by PCR. There is no DNA sequencing.

5.2 Samples for Genetic/Genomic Analysis

5.2.1 Description of the scope of genetic/genomic analysis

5.2.1.1 RNA and T-cell Receptor Clonality Analysis of Blood and Tumor Tissue

Where possible, RNA expression and T-cell receptor clonality analysis will be done on the peripheral blood as well as archived tumor tissue or optional biopsies to help further evaluate

changes in immune response and RNA expression levels with treatment as well as to determine tumor and infiltrating lymphocyte characteristics which may be predictive of response to treatment. In addition, these analyses will also be used to gauge resistance mechanisms and additional targets for future therapy. Coded, linked samples may be analyzed for RNA expression levels using the Nanostring platform and T-cell receptor clonality using the ImmunoSeq platform (LTIB and NCI Frederick Genomic Core Facility).

NCI Fredrick Genomic Core Facility:
Leidos Biomedical Research, Inc:
[redacted]

5.2.2 Description of how privacy and confidentiality of medical information/biological specimens will be maximized

Confidentiality for genetic samples will be maintained as described (Section **5.3.1** and **5.3.2**).

5.3 SAMPLE STORAGE, TRACKING AND DISPOSITION

Samples will be ordered in CRIS and tracked through a Clinical Trial Data Management system. Should a CRIS screen not be available, the CRIS downtime procedures will be followed. Samples will not be sent outside the National Institutes for Health (NIH) without IRB notification and an executed material transfer agreement.

5.3.1 Sample Management and Storage at Clinical Services Program – Leidos Biomedical Research, Inc. (CSP)

Clinical Services Program - Leidos Biomedical Research, Inc.
[redacted]

On days samples are drawn, [redacted] at CSP (part of NCI Frederick Central Repositories) should be notifie [redacted]. She will arrange same-day courier delivery of the specimens.

All data associated with the patient samples is protected by using a secure database. All Clinical Support Laboratory Staff receive annual training in maintaining records' confidentiality. All samples drawn at the NIH Clinical Center will be transported to the Clinical Support Laboratory at the Frederick National Laboratory for Cancer Research by couriers.

Samples will be tracked and managed by Central Repository database, where there is no link to personal identifiable information. All samples will be stored in either a -80ºC freezer or vapor phase liquid nitrogen. These freezers are located at NCI Frederick Central Repository in Frederick, Maryland.

NCI Frederick Central Repositories (managed under a subcontract) store, among other things, biological specimens in support of NIH clinical studies. All specimens are stored in secure, limited-access facilities with sufficient security, backup, and emergency support capability and monitoring to ensure long-term integrity of the specimens for research.

Specimens are stored in accordance with applicable HHS and FDA Protection of Human Subjects Regulations in accordance with the subcontractor's Federal-wide Assurance. The subcontractor's role limited to clinical research databases and repositories containing patient specimens. The subcontractor does not conduct or have any vested interest in research on human subjects, but does provide services and support the efforts of its customers, many of which are involved in research on human subjects.

It is the intent and purpose of the subcontractor to accept only coded, linked samples and sample information. To the limit of our ability, every effort will be made to ensure that protected information is not sent electronically or by hard copy or on vial labels.

Sample data is stored in the BioSpecimen Inventory System II (BSI). This inventory tracking system is used to manage the storage and retrieval of specimens as well as to maintain specimen data. BSI is designed for controlled, concurrent access. It provides a real-time, multi-user environment for tracking millions of specimens. The system controls how and in what order database updates and searches are performed. This control prevents deadlocks and race conditions. For security, BSI has user password access, 3 types of user access levels, and 36 user permissions (levels of access) that can be set to control access to the system functions. BSI provides audit tracking for processes that are done to specimens including shipping, returning to inventory, aliquoting, thawing, additives, and other processes. BSI tracks the ancestry of specimens as they are aliquoted, as well as discrepancies and discrepancy resolution for specimens received by the repository. If a specimen goes out of the inventory, the system maintains data associated with the withdrawal request. Vials are labeled with a unique BSI ID which is printed in both eye-readable and bar-coded format. No patient-specific information is encoded in this ID.

Investigators are granted view, input, and withdrawal authority only for their specimens. They may not view specimen data or access specimens for which they have not been authorized. Access to specimen storage is confined to repository staff. Visitors to the repositories are escorted by repository staff at all times.

5.3.2 Procedures for Storage of Tissue Specimens in the Laboratory of Pathology

Tissues designated for clinical diagnostics are transported to the Laboratory of Pathology (LP) where they are examined grossly and relevant portions are fixed, embedded in paraffin and sectioned and stained for diagnostic interpretation. Unutilized excess tissues are stored for up to three months, in accordance with College of American Pathologists/Joint Commission on Accreditation of Healthcare Organizations (CAP/JCAHO) guidelines, and then discarded. Following completion of the diagnostic workup, the slides and tissue blocks are stored indefinitely in the LP's clinical archives. All specimens are catalogued and retrieved utilizing the clinical laboratory information systems, in accordance with CAP/JCAHO regulations. The use of any stored specimens for research purposes is only allowed when the appropriate IRB approval has been obtained. In some cases, this approval has been obtained via the original protocol on which the patient was enrolled.

5.3.3 Protocol Completion/Sample Destruction

All specimens obtained in the protocol are used as defined in the protocol. Any specimens that are remaining at the completion of the protocol will be stored in the conditions described above. The study will remain open so long as sample or data analysis continues. Samples from consenting subjects will be stored until they are no longer of scientific value or if a subject withdraws consent for their continued use, at which time they will be destroyed. The PI will report any loss or unanticipated destruction of samples per Section **7.2.1**. If the patient withdraws consent the participant's data will be excluded from future distributions, but data that have already been distributed for approved research use will not be able to be retrieved.

Samples will also be reported as lost if they are lost in transit between facilities or misplaced by a researcher. Freezer problems, lost samples, or other problems associated with samples that meet expedited reporting requirements (see Section **7.2.1**) will also be reported.

6 DATA COLLECTION AND EVALUATION

6.1 DATA COLLECTION

The PI will be responsible for overseeing entry of data into an in-house password protected electronic system *(C3D)* and ensuring data accuracy, consistency and timeliness. The principal investigator, associate investigators/research nurses and/or a contracted data manager will assist with the data management efforts. All data obtained during the conduct of the protocol will be kept in secure network drives or in approved alternative sites that comply with NIH security standards. Primary and final analyzed data will have identifiers so that research data can be attributed to an individual human subject participant.

All adverse events, including clinically significant abnormal findings on laboratory evaluations, regardless of severity, will be followed until return to baseline or stabilization of event.

Document AEs from the first study intervention, Study Day 1 through 28 days after removal from study treatment or until off-study, whichever comes first. Adverse events that are serious need to be recorded through 28 days after the last intervention. Beyond 28 days after the last intervention and through long term follow up (survival of subject), only adverse events which are serious and related to the study intervention need to be recorded.

An abnormal laboratory value will be recorded in the database as an AE **only** if the laboratory abnormality is characterized by any of the following:

- Results in discontinuation from the study
- Is associated with clinical signs or symptoms
- Requires treatment or any other therapeutic intervention
- Is associated with death or another serious adverse event, including hospitalization.
- Is judged by the Investigator to be of significant clinical impact
- If any abnormal laboratory result is considered clinically significant, the investigator will provide details about the action taken with respect to the test drug and about the patient's outcome.

End of study procedures: Data will be stored according to HHS, FDA regulations, and NIH Intramural Records Retention Schedule as applicable.

Loss or destruction of data: Should we become aware that a major breach in our plan to protect subject confidentiality and trial data has occurred, this will be reported expeditiously per requirements in Section **7.2.1**.

6.2 DATA SHARING PLANS

6.2.1 Human Data Sharing Plan

What data will be shared?

I will share human data generated in this research for future research as follows:

- Coded, linked data in an NIH-funded or approved public repository.
- Coded, linked data in BTRIS (automatic for activities in the Clinical Center)
- Identified or coded, linked data with approved outside collaborators under appropriate agreements.

How and where will the data be shared?

Data will be shared through:

- An NIH-funded or approved public repository: clinicaltrials.gov
- BTRIS (automatic for activities in the Clinical Center).
- Approved outside collaborators under appropriate individual agreements.
- Publication and/or public presentations.

When will the data be shared?

- Before publication.
- At the time of publication or shortly thereafter.

6.2.2 Genomic Data Sharing Plan

Unlinked genomic data will be deposited in public genomic databases such as dbGaP in compliance with the NIH Genomic Data Sharing Policy.

6.3 RESPONSE CRITERIA

6.3.1 Antitumor Response

Tumor assessments may include the following evaluations: physical examination (with photograph and measurement of skin lesions, as applicable); cross-sectional imaging using computed tomography (CT) or magnetic resonance imaging (MRI) scan of the chest, abdomen, and pelvis (pelvis scan is optional unless known pelvic disease is present at baseline); nuclear bone scan for subjects with known/suspected bone lesions; and CT or MRI scan of the brain (only as clinically warranted based on symptoms/findings). The preferred method of disease assessment is CT with contrast. If CT with contrast is contraindicated, CT of the chest without contrast and MRI scan of the abdomen/pelvis is preferred.

At baseline, tumor lesions will be selected and categorized as target or non-target lesions. Target lesions include those lesions that can be accurately measured in at least 1 dimension as ≥ 20 mm

with conventional techniques or ≥ 10 mm with CT scan. Malignant lymph nodes with a short axis diameter ≥ 15 mm can be considered target lesions. Up to a maximum of 2 target lesions per organ and 5 target lesions in total will be identified at baseline. These lesions should be representative of all involved organs and selected based on their size (those with the longest diameter) and their suitability for accurate repeated measurements. A sum of the longest lesion diameter (LLD) for all target lesions will be calculated and reported as the baseline sum LLD. For malignant lymph nodes identified as target lesions, the short axis diameter will be used in the sum of LLD calculation. All other lesions (or sites of disease) should be identified as non-target lesions (including bone lesions).

All post-baseline response assessments should follow the same lesions identified at baseline. The same mode of assessment (e.g., CT) used to identify/evaluate lesions at baseline should be used throughout the course of the study unless subject safety necessitates a change (e.g., allergic reaction to contrast media).

For the primary endpoint antitumor activity will be evaluated with target and/or non-target lesions according to RECIST Version 1.1.

6.3.2 Disease Parameters

Measurable disease: Measurable lesions are defined as those that can be accurately measured in at least one dimension (longest diameter to be recorded) as:

- By chest x-ray: ≥ 20 mm;
- By CT scan:
  - Scan slice thickness 5 mm or under: as ≥ 10 mm
  - Scan slice thickness > 5 mm: double the slice thickness
- With calipers on clinical exam: ≥ 10 mm.

All tumor measurements must be recorded in millimeters (or decimal fractions of centimeters).

Malignant lymph nodes. To be considered pathologically enlarged and measurable, a lymph node must be ≥ 15 mm in short axis when assessed by CT scan (CT scan slice thickness recommended to be no greater than 5 mm). At baseline and in follow-up, only the short axis will be measured and followed.

Non-measurable disease. All other lesions (or sites of disease), including small lesions (longest diameter < 10 mm or pathological lymph nodes with ≥ 10 to < 15 mm short axis), are considered non-measurable disease. Bone lesions, leptomeningeal disease, ascites, pleural/pericardial effusions, lymphangitis cutis/pulmonitis, inflammatory breast disease, and abdominal masses (not followed by CT or MRI), are considered as non-measurable.

Note: Cystic lesions that meet the criteria for radiographically defined simple cysts should not be considered as malignant lesions (neither measurable nor non-measurable) since they are, by definition, simple cysts.

'Cystic lesions' thought to represent cystic metastases can be considered as measurable lesions, if they meet the definition of measurability described above. However, if non-cystic lesions are present in the same patient, these are preferred for selection as target lesions.

Target lesions. All measurable lesions up to a maximum of 2 lesions per organ and 5 lesions in total, representative of all involved organs, should be identified as **target lesions** and recorded and measured at baseline. Target lesions should be selected on the basis of their size (lesions with the longest diameter), be representative of all involved organs, but in addition should be those that lend themselves to reproducible repeated measurements. It may be the case that, on occasion, the largest lesion does not lend itself to reproducible measurement in which circumstance the next largest lesion which can be measured reproducibly should be selected. A sum of the diameters (longest for non-nodal lesions, short axis for nodal lesions) for all target lesions will be calculated and reported as the baseline sum diameters. If lymph nodes are to be included in the sum, then only the short axis is added into the sum. The baseline sum diameters will be used as reference to further characterize any objective tumor regression in the measurable dimension of the disease.

Non-target lesions. All other lesions (or sites of disease) including any measurable lesions over and above the 5 target lesions should be identified as **non-target lesions** and should also be recorded at baseline. Measurements of these lesions are not required, but the presence, absence, or in rare cases unequivocal progression of each should be noted throughout follow-up.

6.3.3 Methods for Evaluation of Measurable Disease

All measurements should be taken and recorded in metric notation using a ruler or calipers. All baseline evaluations should be performed as closely as possible to the beginning of treatment and never more than 4 weeks before the beginning of the treatment.

The same method of assessment and the same technique should be used to characterize each identified and reported lesion at baseline and during follow-up. Imaging-based evaluation is preferred to evaluation by clinical examination unless the lesion(s) being followed cannot be imaged but are assessable by clinical exam.

Clinical lesions: Clinical lesions will only be considered measurable when they are superficial (e.g., skin nodules and palpable lymph nodes) and ≥10 mm diameter as assessed using calipers (e.g., skin nodules). In the case of skin lesions, documentation by color photography, including a ruler to estimate the size of the lesion, is recommended.

Chest x-ray: Lesions on chest x-ray are acceptable as measurable lesions when they are clearly defined and surrounded by aerated lung. However, CT is preferable.

Conventional CT and MRI: This guideline has defined measurability of lesions on CT scan based on the assumption that CT slice thickness is 5 mm or less. If CT scans have slice thickness greater than 5 mm, the minimum size for a measurable lesion should be twice the slice thickness. MRI is also acceptable in certain situations (e.g., for body scans).

Use of MRI remains a complex issue. MRI has excellent contrast, spatial, and temporal resolution; however, there are many image acquisition variables involved in MRI, which greatly impact image quality, lesion conspicuity, and measurement. Furthermore, the availability of MRI is variable globally. As with CT, if an MRI is performed, the technical specifications of the scanning sequences used should be optimized for the evaluation of the type and site of disease. Furthermore, as with CT, the modality used at follow-up should be the same as was used at baseline and the lesions should be measured/assessed on the same pulse sequence. It is beyond the scope of the RECIST guidelines to prescribe specific MRI pulse sequence parameters for all

scanners, body parts, and diseases. Ideally, the same type of scanner should be used and the image acquisition protocol should be followed as closely as possible to prior scans. Body scans should be performed with breath-hold scanning techniques, if possible.

6.3.4 Response Criteria

All the scans performed at Baseline and other imaging performed as clinically required (other supportive imaging) need to be repeated at subsequent visits. In general, lesions detected at Baseline need to be followed using the same imaging methodology and preferably the same imaging equipment at subsequent tumor evaluation visits.

Brain CT / MRI scan should be performed, if clinically indicated by development of new specific symptoms or on the discretion of the Principal Investigator. For each subject, the Investigator will designate 1 or more of the following measures of tumor status to follow for determining response: CT or MRI images of primary and / or metastatic tumor masses, physical examination findings, and the results of other assessments. All available images collected during the trial period will be considered. The most appropriate measures to evaluate the tumor status of a subject should be used. The measure(s) to be chosen for sequential evaluation during the trial have to correspond to the measures used to document the progressive tumor status that qualifies the subject for enrollment. The tumor response assessment will be assessed and listed according to the Study Calendar **3.5**.

The foreseen treatment duration is until disease progression verified by a scan subsequent to the initial documentation of PD, unacceptable toxicity, or any criterion for withdrawal from the trial occurs (see Section **6.3**). Before stopping the treatment, progressive disease should be confirmed by imaging 4 to 6 weeks (preferably 6 weeks, but not later) after progression has been diagnosed according to RECIST 1.1 [35]. If progression is based on the occurrence of a new lesion in an area not scanned at Baseline, a further on-study scan 6 weeks later should be considered before performing the 28-Day Safety Follow-up visit. Treatment may be continued despite progression according to RECIST 1.1 at any time if:

- There are no new or concerning symptoms.
- There is no decrease in ECOG PS.
- The Investigator does not consider it necessary to administer a salvage therapy.

The treatment should be stopped immediately, if the subject does not tolerate M7824 or if therapeutic failure occurs, which requires urgent treatment with an additional drug or results in clinically significant progression / deterioration.

Tumor responses to treatment will be assigned based on the evaluation of the response of target, non-target, and new lesions according to RECIST 1.1 (all measurements should be recorded in metric notation).

•To assess objective response, the tumor burden at baseline will be estimated and used for comparison with subsequent measurements. At baseline, tumor lesions will be categorized in target and non-target lesions according to RECIST 1.1.

Results for these evaluations will be recorded with as much specificity as possible so that pre- and post-treatment results will provide the best opportunity for evaluating tumor response.

Any CR or PR should be confirmed according to RECIST 1.1. In the case of a PR or CR, a confirmatory CT or MRI scan should be done no sooner than 4 weeks (preferably at the scheduled 6-week interval).

The Investigator may perform scans in addition to a scheduled trial scan for medical reasons or if the Investigator suspects PD.

As an exploratory endpoint antitumor activity will also be evaluated according to iRECIST [36].

Using iRECIST criteria the following will be incorporated into assessment:

1. An increase in the sum of target lesions of more than 20%, unequivocal increase in non-target lesions or new lesions result in iUPD (unconfirmed progressive disease); iUPD can be assigned multiple times as long as iCPD (confirmed progressive disease) is not confirmed at the next assessment.

2. Progression is confirmed in the target lesion category if the next imaging assessment after iUPD (4–8 weeks later) confirms a further increase in sum of measures of target disease from iUPD, with an increase of at least 5 mm. Progression is confirmed in the non-target lesion category if subsequent imaging, done 4–8 weeks after iUPD, shows a further unequivocal increase in non-target lesions. Progression is confirmed in the new lesions category if at next assessment additional new lesions appear or an increase in size of previously seen new lesions is seen (≥5 mm for sum of new lesion target).

3. However, the criteria for iCPD (after iUPD) are not considered to have been met if complete response, partial response, or stable disease criteria (compared with baseline and as defined by RECIST 1.1) are met at the next assessment after iUPD. The status is reset (unlike RECIST 1.1, in which any progression precludes later complete response, partial response, or stable disease). iCR, iPR, or iSD should then be assigned; and if no change is detected, then the timepoint response is iUPD.

6.3.5 Response Criteria by RECIST 1.1

6.3.5.1 Evaluation of Target Lesions

Complete Response (CR): Disappearance of all target lesions. Any pathological lymph nodes (whether target or non-target) must have reduction in short axis to <10 mm.

Partial Response (PR): At least a 30% decrease in the sum of the diameters of target lesions, taking as reference the baseline sum of diameters.

Progressive Disease (PD): At least a 20% increase in the sum of the diameters of target lesions, taking as reference the smallest sum on study (this includes the baseline sum if that is the smallest on study). In addition to the relative increase of 20%, the sum must also demonstrate an absolute increase of at least 5 mm. (Note: the appearance of one or more new lesions is also considered progressions).

Stable Disease (SD): Neither sufficient shrinkage to qualify for PR nor sufficient increase to qualify for PD, taking as reference the smallest sum of diameters while on study.

6.3.5.2 Evaluation of Non-Target Lesions

Complete Response (CR): Disappearance of all non-target lesions and normalization of tumor marker level. All lymph nodes must be non-pathological in size (<10 mm short axis).

Note: If tumor markers are initially above the upper normal limit, they must normalize for a patient to be considered in complete clinical response.

Non-CR/Non-PD: Persistence of one or more non-target lesion(s) and/or maintenance of tumor marker level above the normal limits.

Progressive Disease (PD): Appearance of one or more new lesions and/or *unequivocal progression* of existing non-target lesions. *Unequivocal progression* should not normally trump target lesion status. It must be representative of overall disease status change, not a single lesion increase.

Although a clear progression of “non-target” lesions only is exceptional, the opinion of the treating physician should prevail in such circumstances, and the progression status should be confirmed at a later time by the review panel (or Principal Investigator).

6.3.5.3 Evaluation of Best Overall Response

The best overall response is the best response recorded from the start of the treatment until disease progression/recurrence (taking as reference for progressive disease the smallest measurements recorded since the treatment started). The patient's best response assignment will depend on the achievement of both measurement and confirmation criteria.

For Patients with Measurable Disease (i.e., Target Disease)

<table>
<tr><th>Target Lesions</th><th>Non-Target Lesions</th><th>New Lesions</th><th>Overall Response</th><th>Best Overall Response when Confirmation is Required*</th></tr>
<tr><td>CR</td><td>CR</td><td>No</td><td>CR</td><td>≥4 wks. Confirmation**</td></tr>
<tr><td>CR</td><td>Non-CR/Non-PD</td><td>No</td><td>PR</td><td rowspan="3">≥4 wks. Confirmation**</td></tr>
<tr><td>CR</td><td>Not evaluated</td><td>No</td><td>PR</td></tr>
<tr><td>PR</td><td>Non-CR/Non-PD/not evaluated</td><td>No</td><td>PR</td></tr>
<tr><td>SD</td><td>Non-CR/Non-PD/not evaluated</td><td>No</td><td>SD</td><td>Documented at least once ≥4 wks. from baseline**</td></tr>
</table>

Target Lesions	Non-Target Lesions	New Lesions	Overall Response	Best Overall Response when Confirmation is Required*
PD	Any	Yes or No	PD	no prior SD, PR or CR
Any	PD***	Yes or No	PD	
Any	Any	Yes	PD	

* See RECIST 1.1 manuscript for further details on what is evidence of a new lesion.

** Only for non-randomized trials with response as primary endpoint.

*** In exceptional circumstances, unequivocal progression in non-target lesions may be accepted as disease progression.

Note: Patients with a global deterioration of health status requiring discontinuation of treatment without objective evidence of disease progression at that time should be reported as "*symptomatic deterioration.*" Every effort should be made to document the objective progression even after discontinuation of treatment.

For Patients with Non-Measurable Disease (i.e., Non-Target Disease)

Non-Target Lesions	New Lesions	Overall Response
CR	No	CR
Non-CR/non-PD	No	Non-CR/non-PD*
Not all evaluated	No	not evaluated
Unequivocal PD	Yes or No	PD
Any	Yes	PD

* 'Non-CR/non-PD' is preferred over 'stable disease' for non-target disease since SD is increasingly used as an endpoint for assessment of efficacy in some trials so to assign this category when no lesions can be measured is not advised

6.3.5.4 Duration of Response

Duration of overall response: The duration of overall response is measured from the time measurement criteria are met for CR or PR (whichever is first recorded) until the first date that recurrent or progressive disease is objectively documented (taking as reference for progressive disease the smallest measurements recorded since the treatment started).

The duration of overall CR is measured from the time measurement criteria are first met for CR until the first date that progressive disease is objectively documented.

Duration of stable disease: Stable disease is measured from the start of the treatment until the criteria for progression are met, taking as reference the smallest measurements recorded since the treatment started, including the baseline measurements.

6.3.5.5 Progression-Free Survival

PFS is defined as the duration of time from start of treatment to time of progression or death, whichever occurs first.

6.4 TOXICITY CRITERIA

The following adverse event management guidelines are intended to ensure the safety of each patient while on the study. The descriptions and grading scales found in the revised NCI Common Terminology Criteria for Adverse Events (CTCAE) version 5.0 will be utilized for AE reporting. All appropriate treatment areas should have access to a copy of the CTCAE version 5.0. A copy of the CTCAE version 5.0 can be downloaded from the CTEP web site (http://ctep.cancer.gov/protocolDevelopment/electronic_applications/ctc.htm).

7 NIH REPORTING REQUIREMENTS / DATA AND SAFETY MONITORING PLAN

7.1 DEFINITIONS

Please refer to definitions provided in Policy 801: Reporting Research Events found at: https://irbo.nih.gov/confluence/display/IRBO/Policies+and+SOPs.

7.2 OHSRP OFFICE OF COMPLIANCE AND TRAINING / IRB REPORTING

7.2.1 Expedited Reporting

Please refer to the reporting requirements in Policy 801: Reporting Research Events and Policy 802 Non-Compliance Human Subjects Research found at: https://irbo.nih.gov/confluence/display/IRBO/Policies+and+SOPs.

Note: Only IND Safety Reports that meet the definition of an unanticipated problem will need to be reported per these policies.

7.2.2 IRB Requirements for PI Reporting at Continuing Review

Please refer to the reporting requirements in Policy 801: Reporting Research Events found at: https://irbo.nih.gov/confluence/display/IRBO/Policies+and+SOPs.

7.2.3 NCI Clinical Director Reporting

Problems expeditiously reported to the OHSRP in iRIS will also be reported to the NCI Clinical Director. A separate submission is not necessary as reports in iRIS will be available to the Clinical Director.

In addition to those reports, all deaths that occur within 30 days after receiving a research intervention should be reported via email to the Clinical Director unless they are due to progressive disease. To report these deaths, please send an email describing the circumstances of

the death to Dr. Dahut at NCICCRQA@mail.nih.gov within one business day of learning of the death.

7.3 NIH Required Data and Safety Monitoring Plan

7.3.1 Principal Investigator/Research Team

The clinical research team will meet on a regular basis (approximately weekly) when patients are being actively treated on the trial to discuss each patient. Decisions about dose level enrollment and dose escalation if applicable will be made based on the toxicity data from prior patients.

All data will be collected in a timely manner and reviewed by the principal investigator or a lead associate investigator. Events meeting requirements for expedited reporting as described in Section **7.2.2** will be submitted within the appropriate timeline.

The principal investigator will review adverse event and response data on each patient to ensure safety and data accuracy. The principal investigator will personally conduct or supervise the investigation and provide appropriate delegation of responsibilities to other members of the research staff.

7.3.2 Safety Monitoring Committee (SMC)

This protocol will be periodically reviewed by an intramural Safety Monitoring Committee. Initial review will occur as soon as possible after the annual NIH Intramural IRB continuing review date. Subsequently, each protocol will be reviewed as close to annually as the quarterly meeting schedule permits or more frequently as may be required by the SMC based on the risks presented in the study. For initial and subsequent reviews, protocols will not be reviewed if there is no accrual within the review period.

The SMC review will focus on unexpected protocol safety issues that are identified during the conduct of the clinical trial.

Written outcome letters will be generated in response to the monitoring activities and submitted to the Principal investigator and Clinical Director or Deputy Clinical Director, CCR, NCI.

8 SPONSOR PROTOCOL SAFETY REPORTING

8.1 Definitions

8.1.1 Adverse Event

Any untoward medical occurrence in a patient or clinical investigation subject administered a pharmaceutical product and which does not necessarily have a causal relationship with this treatment. An adverse event (AE) can therefore be any unfavorable and unintended sign (including an abnormal laboratory finding), symptom, or disease temporally associated with the use of a medicinal (investigational) product, whether or not related to the medicinal (investigational) product (ICH E6 (R2)).

8.1.2 Serious Adverse Event (SAE)

An adverse event or suspected adverse reaction is considered serious if in the view of the investigator or the sponsor, it results in any of the following:

- Death;
- A life-threatening adverse event (see Section **8.1.3**);
- Inpatient hospitalization or prolongation of existing hospitalization
    - A hospitalization/admission that is pre-planned (i.e., elective or scheduled surgery arranged prior to the start of the study), a planned hospitalization for pre-existing condition, or a procedure required by the protocol, without a serious deterioration in health, is not considered a serious adverse event.
    - A hospitalization/admission that is solely driven by non-medical reasons (e.g., hospitalization for patient convenience) is not considered a serious adverse event.
    - Emergency room visits or stays in observation units that do not result in admission to the hospital would not be considered a serious adverse event. The reason for seeking medical care should be evaluated for meeting one of the other serious criteria.
- Persistent or significant incapacity or substantial disruption of the ability to conduct normal life functions;
- A congenital anomaly/birth defect;
- Important medical events that may not result in death, be life-threatening, or require hospitalization may be considered a serious adverse drug experience when, based upon appropriate medical judgment, they may jeopardize the patient or subject and may require medical or surgical intervention to prevent one of the outcomes listed in this definition.

8.1.3 Life-threatening

An adverse event or suspected adverse reaction is considered "life-threatening" if, in the view of either the investigator or sponsor, its occurrence places the patient or subject at immediate risk of death. It does not include an adverse event or suspected adverse reaction that, had it occurred in a more severe form, might have caused death (21CFR312.32).

8.1.4 Severity

The severity of each Adverse Event will be assessed utilizing the CTCAE version 5.0.

8.1.5 Relationship to Study Product

All AEs will have their relationship to study product assessed using the terms: related or not related.

- <u>Related</u> – There is a reasonable possibility that the study product caused the adverse event. Reasonable possibility means that there is evidence to suggest a causal relationship between the study product and the adverse event.

- <u>Not Related</u> – There is not a reasonable possibility that the administration of the study product caused the event.

8.2 Assessment of Safety Events

AE information collected will include event description, date of onset, assessment of severity and relationship to study product and alternate etiology (if not related to study product), date of resolution of the event, seriousness and outcome. The assessment of severity and relationship to the study product will be done only by those with the training and authority to make a diagnosis and listed on the Form FDA 1572 as the site principal investigator or sub-investigator. AEs occurring during the collection and reporting period will be documented appropriately regardless of relationship. AEs will be followed through resolution.

SAEs will be:

- Assessed for severity and relationship to study product and alternate etiology (if not related to study product) by a licensed study physician listed on the Form FDA 1572 as the site principal investigator or sub-investigator.
- Recorded on the appropriate SAE report form, the medical record and captured in the clinical database.
- Followed through resolution by a licensed study physician listed on the Form FDA 1572 as the site principal investigator or sub-investigator.

For timeframe of recording adverse events, please refer to Section **6.1**. All serious adverse events recorded must be reported to the sponsor. All serious adverse events recorded from the time of first investigational product administration must be reported to the sponsor with the exception of any listed in section **8.4**.

8.3 Reporting of Serious Adverse Events

Any AE that meets protocol-defined serious criteria or meets the definition of Adverse Event of Special Interest that require expedited reporting must be submitted immediately (within 24 hours of awareness) to OSRO Safety using the CCR SAE report form. Any exceptions to the expedited reporting requirements are found in section **8.4**.

All SAE reporting must include the elements described in Section **8.2**.

SAE re orts will be submitted to the Center for Cancer Research (CCR) at: [redacted] and to the CCR PI and study coordinator. CCR SAE report form and instructions can be found at: https://ccrod.cancer.gov/confluence/pages/viewpage.action?pageId=157942842

Following the assessment of the SAE by OSRO, other supporting documentation of the event may be requested by the OSRO Safety and should be provided as soon as possible.

8.4 Waiver of Expedited Reporting to CCR

As overall survival, which includes death due to disease progression and hospitalization due to disease progression are part of the study objectives, and captured as an endpoint in this study, death/hospitalization due to disease progression will not be reported in expedited manner to the sponsor. However, if there is evidence suggesting a causal relationship between the study drug and the event, report the event in an expedited manner according to section **8.3**.

8.5 Safety Reporting Criteria to the Pharmaceutical Collaborators

Reporting will be per the collaborative agreement.

8.6 Reporting Pregnancy

8.6.1 Maternal exposure

If a patient becomes pregnant during the course of the study, the study treatment should be discontinued immediately and the pregnancy reported to the Sponsor. The potential risk of exposure of the fetus to the investigational agent(s) or chemotherapy agents (s) should be documented in box B5 of the MedWatch form "Describe Event or Problem".

Pregnancy itself is not regarded as an SAE. However, congenital abnormalities or birth defects and spontaneous miscarriages that meet serious criteria (Section **8.1.2**) should be reported as SAEs.

The outcome of all pregnancies (spontaneous miscarriage, elective termination, ectopic pregnancy, normal birth, or congenital abnormality) should be followed up and documented.

8.6.2 Paternal exposure

Male patients should refrain from fathering a child or donating sperm during the study and for 2 months after the last dose of M7824.

Pregnancy of the patient's partner is not considered to be an AE. However, the outcome of all pregnancies (spontaneous miscarriage, elective termination, ectopic pregnancy, normal birth, or congenital abnormality) occurring from the date of the first dose until 2 months after the last dose should, if possible, be followed up and documented.

8.7 Regulatory Reporting for Studies Conducted Under CCR-Sponsored IND

Following notification from the investigator, CCR, the IND sponsor, will report any suspected adverse reaction that is both serious and unexpected in expedited manner to the FDA in accordance to 21 CFR 31.2.32. CCR will report an AE as a suspected adverse reaction only if there is evidence to suggest a causal relationship between the study product and the adverse event. CCR will notify FDA and all participating investigators (i.e., all investigators to whom the sponsor is providing drug under its INDs or under any investigator's IND) in an IND safety report of potential serious risks from clinical trials or any other source, as soon as possible, in accordance to 21 CFR Part 312.32.

All serious events will be reported to the FDA at least annually in a summary format.

8.8 Sponsor Protocol Non-Adherence Reporting

Protocol non-adherence is defined as any noncompliance with the clinical trial protocol, GCP, or protocol-specific procedural requirements on the part of the participant, the Investigator, or the study site staff inclusive of site personnel performing procedures or providing services in support of the clinical trial.

It is the responsibility of the study Staff to document any protocol non-adherence identified by the Staff or the site Monitor on the OSRO Site Protocol Non-Adherence Log. The protocol-specific, cumulative non-adherence log should be maintained in the site essential documents file

and submitted to OSRO vi [redacted] n the **first business day of each month over the duration of the study**. In addition, any non-adherence to the protocol should be documented in the participant's source records and reported to the local IRB per their guidelines. OSRO required protocol non-adherence reporting is consistent with E6(R2) GCP: Integrated Addendum to ICH E6(R1): 4.5 Compliance with Protocol; 5.18.3 (a), and 5.20 Noncompliance; and ICH E3 16.2.2 Protocol deviations.

9 CLINICAL MONITORING PLAN

Clinical site monitoring is conducted to ensure that the rights of the participants are protected, that the study is implemented per the approved protocol, Good Clinical Practice and standard operating procedures, and that the quality and integrity of study data and data collection methods are maintained. Monitoring for this study will be performed by NCI CCR Office of Sponsor and Regulatory Oversight (OSRO) Monitoring based on OSRO standards, FDA Guidance E6(R2) Good Clinical Practice: Integrated Addendum to ICH E6(R1) March 2018, and applicable regulatory requirements.

Details of clinical site monitoring will be documented in a Clinical Monitoring Plan (CMP) developed by OSRO. CMPs will be protocol-specific, risk-based and tailored to address human subject protections and integrity of the study data. The intensity and frequency of monitoring will be based on several factors, including study type, phase, risk, complexity, expected enrollment rate, and any unique attributes of the study and the site. OSRO Monitoring visits and related activities will be conducted throughout the life cycle of each protocol, with the first activity being before study start to conduct a Site Assessment Visit (SAV) (as warranted), followed by a Site Initiation Visit (SIV), Interim Monitoring Visit(s) (IMVs), and a study Close-Out Visit (COV).

Some monitoring activities may be performed remotely, while others will take place at the study site(s). Monitoring visit reports will describe visit activities, observations, findings of protocol non-adherence and associated action items or follow-up required for resolution of findings. Monitoring reports will be distributed to the study PI, NCI CCR QA, coordinating center (if applicable) and the OSRO regulatory file.

If protocol non-adherence is identified by the Monitor (i.e., any noncompliance with the clinical trial protocol, GCP, or protocol-specific procedural requirements on the part of the participant, the Investigator, or the site Staff) the Monitor will note the observation, review with site Staff and if unresolved, request that the Staff document the non-adherence on the protocol-specific OSRO Site Protocol Non-Adherence Log (see Section 8.8).

10 STATISTICAL CONSIDERATIONS

10.1 Statistical Hypothesis

The primary objective of this trial is to determine the objective response rate of combination immunotherapy in patients with HPV associated malignancies. A secondary hypothesis (objective) will be to obtain preliminary data on duration of response, PFS, OS and ratio of patients that are hospitalized because of adverse events attributed to disease progression. An exploratory

hypothesis (objective) will be to evaluate exploratory immunologic studies to understand and improve the administered treatment.

10.2 Sample Size Determination

Data from the ongoing trial indicates that a response rate for M7824 may be approximately 30%. This trial will try to demonstrate if the proposed combination therapies may be associated with an improved response rate.

The trial will treat patients with HPV related cancers with HPV vaccine + M7824 + NHS-IL12. The objective is to determine if this treatment can be associated with an acceptable response rate. To establish the efficacy of this combination treatment, the primary objective would be to determine if using the proposed agents would rule out a 30% response rate and result in a response rate consistent with 60%. As such, this trial in patients with HPV associated malignancies will be conducted using a Simon optimal two-stage phase II trial design (Simon R, Controlled Clinical Trials 10:1-10, 1989) to rule out an unacceptably low PR+CR rate of 30% (p0=0.30) in favor of an improved response rate of 60% (p1=0.60). With alpha=0.10 (probability of accepting a poor treatment=0.10) and beta = 0.10 (probability of rejecting a good treatment=0.10), the first stage will enroll 8 evaluable patients, and if 0 to 2 of the 8 have a clinical response, then no further patients will be accrued. If 3 or more of the first 8 patients have a response, then accrual would continue until a total of 20 evaluable patients have been treated. As it may take up to several months to determine if a patient has experienced a response, a temporary pause in the accrual may be necessary to ensure that enrollment to the second stage is warranted. If there are 3 to 8 patients with a response out of 20 patients, this would be an uninterestingly low response rate. If there were 9 or more of 20 (45.0%) who experienced a response, this would be sufficiently interesting to warrant possible further study of the combination in later trials in this disease type; decisions about future use of the combination will incorporate response as well as toxicity profile information. Under the null hypothesis (30% response rate), the probability of early termination is 55.2%.

It is expected that if 2 patients per month enroll on the trial, in order to enroll up to a total of 20 evaluable patients up to 1 year may be required. To allow for a number of inevaluable patients and potential replacements based on HPV status, the accrual ceiling will be set at 29 patients.

10.3 Populations for Analyses

All patients who receive any investigational treatment will be evaluable for safety and toxicity evaluations. All patients will be evaluable for toxicity from the time of their first treatment with any agent.

For efficacy, a modified intention to treat population will be used. Only those patients who have measurable disease present at baseline and have had their disease re-evaluated will be considered evaluable for response. (Note: Patients who exhibit objective disease progression prior to the end of cycle 1 will also be considered evaluable.)

10.4 Statistical Analyses

10.4.1 General Approach

Patients will be evaluated with respect to the grades and types of toxicities obtained. The results will be presented descriptively and tabled if appropriate. Further planned analyses are described below.

10.4.2 Analysis of the Primary Endpoints

The percentage of subjects that achieve an objective confirmed complete or partial overall tumor response using RECIST Version 1.1 will be evaluated. If anti-tumor responses are observed, the 95% confidence interval of the response rate will be evaluated.

10.4.3 Analysis of the Secondary Endpoints

Data will be obtained on duration of response, PFS, OS and ratio of patients that are hospitalized because of adverse events attributed to disease progression

10.4.3.1 Duration of Response

The duration of overall response is measured from the time measurement criteria are met for CR or PR (whichever is first recorded) until the first date that PD is objectively documented and is evaluated using the Kaplan-Meier method.

10.4.3.2 Progression-Free Survival

PFS will be evaluated using Kaplan-Meier methods. PFS will be defined as the time from the date of first treatment to the date of disease progression or death (any cause) whichever occurs first. Subjects who do not have disease progression or have not died at the end of follow up will be censored at the last known date the subject was progression free.

10.4.3.3 Objective Response

Objective response is a complete or partial radiographic response as defined by RECIST 1.1 (Section **6.3.5**).

10.4.3.4 Overall Survival

OS will be evaluated using Kaplan-Meier methods. OS will be defined as the time from the date of first treatment to the date of death (any cause). Subjects who are alive at the end of follow up will be censored at the last known date alive.

10.4.4 Safety Analyses

Safety endpoints will be analyzed as summary statistics during treatment and/or as change scores from baseline assessments. AEs will be coded as defined in the Medical Dictionary for Regulatory Activities (MedDRA). All AEs will be recorded and tabulated following each treatment. AEs will be recorded by severity, frequency, and relationship to the study intervention and will be presented by System Organ Class (SOC) designations and preferred term groupings. Information on each AE will include start date, stop date, severity, relationship, expectedness, outcome, and duration. Adverse events leading to premature discontinuation from the study intervention and serious treatment-emergent AEs will be presented either in a Table or a Listing.

In addition, overall safety will be assessed by descriptive analyses using tabulated frequencies of AEs by grade using CTCAE Version 5 within dose cohorts and for the overall study population in terms of treatment-emergent AEs, SAEs, and clinically significant changes in safety laboratory tests, physical examinations, ECGs, and vital signs.

10.4.5 Baseline Descriptive Statistics

Baseline Characteristics will be described.

10.4.6 Planned Interim Analyses

Interim assessment of efficacy will be made according to the Simon two stage phase II designs of each cohort described in Section **10.4.6.**

10.4.7 Tabulation of Individual Participant Data

Individual responses in a cohort may be depicted within a larger group using a waterfall plot or spider diagram.

10.4.8 Exploratory Analyses (Immune Responses)

Where feasible exploratory immune analyses will be conducted to evaluate anti-tumor immune responses. Immune response will be assessed among all subjects treated in each cohort. The magnitude of immune responses will be described. A subject will be considered evaluable for immune response if they receive at least one dose of treatment. The percentage of subjects with a positive immune response will be evaluated by cohort. For flow cytometry analyses on PBMC samples, Student T tests or Kruskal-Wallis and Wilcoxon rank sum tests as appropriate will be performed on percentages of TNF-α and/or IFN-γ expressing cells among the different cohorts to determine any significant differences in cell populations. For antigen specific T cell responses, a positive immune response is defined by CMI reactivity in *ex vivo* stimulation using a flow cytometric readout (cytokine production or CD107 expression). Antigen-specific peptide challenge assays require a readout of >250 reactive T-cells/million cells above the background [37].

11 COLLABORATIVE AGREEMENTS

11.1 Cooperative Research and Development Agreement (CRADA)

A CRADA (02666) is in place with EMD Serono for the supply of M7824 and NHS-IL12.

A CRADA (03279) is in place with PDS Biotechnology for the supply of PDS0101.

12 HUMAN SUBJECTS PROTECTIONS

12.1 Rationale For Subject Selection

Subjects from all racial/ethnic groups are eligible for this study if they meet the eligibility criteria. Efforts will be made to extend accrual to a representative population. Due to impaired cellular immunity which may affect the efficacy of treatment, patients with poorly controlled HIV as well as patents with detectable viral loads of hepatitis B and C will be excluded.

12.2 PARTICIPATION OF CHILDREN

Individuals under the age of 18 will not be eligible to participate in this study because of unknown toxicities in pediatric patients.

12.3 PARTICIPATION OF SUBJECTS UNABLE TO GIVE CONSENT

Adults unable to give consent are excluded from enrolling in the protocol. However, re-consent may be necessary and there is a possibility, though unlikely, that subjects could become decisionally impaired. For this reason and because there is a prospect of direct benefit from research participation (Section **12.4.2**), all subjects ≥ age 18 will be offered the opportunity to fill in their wishes for research and care, and assign a substitute decision maker on the "NIH Advance Directive for Health Care and Medical Research Participation" form so that another person can make decisions about their medical care in the event that they become incapacitated or cognitively impaired during the course of the study. Note: The PI or AI will contact the NIH Ability to Consent Assessment Team (ACAT) for evaluation as needed for the following: an independent assessment of whether an individual has the capacity to provide consent; assistance in identifying and assessing an appropriate surrogate when indicated; and/or an assessment of the capacity to appoint a surrogate. For those subjects that become incapacitated and do not have pre-determined substitute decision maker, the procedures described in NIH HRPP SOP 14E for appointing a surrogate decision maker for adult subjects who are (a) decisionally impaired, and (b) who do not have a legal guardian or durable power of attorney, will be followed.

12.4 RISKS/BENEFIT ASSESSMENT

Patients will receive evaluation of their disease at the National Cancer Institute's Clinical Center. The potential benefit to a patient that goes onto study is a reduction in the bulk of their tumor which may or may not have a favorable impact on symptoms and/or survival.

Potential adverse reactions attributable to the administration of the study drug utilized in this trial are discussed in Section **14**. All care will be taken to minimize side effects, but they can be unpredictable in nature and severity. Patients will be examined and evaluated prior to enrollment. All evaluations to monitor the treatment of patients will be recorded in the medical record. If patients suffer any physical injury as a result of the participation in this study, immediate medical treatment is available at the Clinical Center, National Cancer Institute, Bethesda, Maryland.

Although no compensation is available, any injury will be evaluated and treated in keeping with the benefits or care to which patients are entitled under applicable regulations.

12.4.1 Risks

12.4.1.1 Risk of Optional Biopsies

All care will be taken to minimize risks that may be incurred by tumor sampling. However, there are procedure-related risks (such as bleeding, infection and visceral injury) that will be explained fully during informed consent.

12.4.1.2 Risks of Exposure to Ionizing Radiation

This research study involves two CT guided biopsies collected for research purposes. Subjects undergoing two optional biopsies collections will be exposed to 1.6 rem. This amount of radiation

is below the guideline of 5 rem per year allowed for adult research subjects by the NIH Radiation Safety Committee.

12.4.1.3 Research Blood Collection Risks

Risks of blood draws include pain and bruising in the area where the needle is placed, lightheadedness, and rarely, fainting. When large amounts of blood are collected, low red blood cell count (anemia) can develop.

12.4.1.4 Other Risks

Risks include the possible occurrence of any of a range of side effects which are listed in the Consent Document or this protocol document. Frequent monitoring for adverse effects will help to minimize the risks associated with administration of the study agents.

12.4.2 Benefits

The potential benefit to a patient that goes onto study is a reduction in the bulk of their tumor which may or may not have favorable impact on symptoms and/or survival.

12.4.3 Assessment of Potential Risks and Benefits

Metastatic or refractory/recurrent HPV associated malignancies (cervical, anal, oropharyngeal cancers, etc.) are in need of improved therapy options. Preclinical studies suggest that the use of a combination of multiple immunotherapy agents may have improved anti-tumor efficacy.

A number of clinically appropriate strategies to minimize risks to patients have been built into the protocol through the means of inclusion/exclusion criteria, monitoring strategies, and management guidelines. Overall, the potential benefits of the combination of a therapeutic vaccine against HPV positive cancers (PDS0101), a tumor targeted immunocytokine (NHS-IL12) and a bifunctional fusion protein targeting PD-L1 and TGF beta (M7824) in subjects with advanced HPV associated malignancies for patients retaining the ability to consent and those who lose capacity to consent during the course of the trial outweigh the risks associated with this combination immunotherapy proposed in this study.

The potential benefit to a patient that goes onto this study is a reduction in the bulk of their tumor which may or may not have favorable impact on symptoms and/or survival.

Potential adverse reactions attributable to the administration of the study drug utilized in this trial are discussed in Section **14**. All care will be taken to minimize side effects, but they can be unpredictable in nature and severity.

12.5 Consent and Assent Process and Documentation

The investigational nature and research objectives of this trial, the procedures and treatments involved and their attendant risks and discomforts and benefits, and potential alternative therapies will be carefully explained to the patient, and a signed informed consent document will be obtained by a study investigator prior to entry onto the study.

The PI or associate investigator will meet with the patient to discuss the protocol treatment and alternative options in detail. It will be stated clearly that participation in the research study is voluntary and that participants can withdraw from the study without losing benefits they would

otherwise be entitled to. The patient will be encouraged to ask questions, and additional meetings to discuss the treatment options will be arranged as necessary.

As there is an optional biopsy for research in this protocol, the patient will be asked to sign a separate consent at the time of the procedure. If the patient refuses the optional biopsy at that time, the refusal will be documented in the medical record and in the research record.

12.5.1 Telephone Re-consent Procedure

Re-consent on this study may be obtained via telephone according to the following procedure: the informed consent document will be sent to the subject. An explanation of the study will be provided over the telephone after the subject has had the opportunity to read the consent form. The subject will sign and date the informed consent.

The original informed consent document will be sent back to the consenting investigator who will sign and date the consent form with the date the consent was obtained via telephone.

A fully executed copy will be returned via mail for the subject's records.

The informed consent process will be documented in the medical record.

13 REGULATORY AND OPERATIONAL CONSIDERATIONS

13.1 STUDY DISCONTINUATION AND CLOSURE

This study may be temporarily suspended or prematurely terminated if there is sufficient reasonable cause. Written notification, documenting the reason for study suspension or termination, will be provided by the suspending or terminating party to study participants, investigator, the Investigational New Drug (IND) and regulatory authorities. If the study is prematurely terminated or suspended, the Principal Investigator (PI) will promptly inform study participants, the Institutional Review Board (IRB), and sponsor and will provide the reason(s) for the termination or suspension. Study participants will be contacted, as applicable, and be informed of changes to study visit schedule.

Circumstances that may warrant termination or suspension include, but are not limited to:

- Determination of unexpected, significant, or unacceptable risk to participants
- Demonstration of efficacy that would warrant stopping
- Insufficient compliance to protocol requirements
- Data that are not sufficiently complete and/or evaluable
- Determination that the primary endpoint has been met
- Determination of futility

Study may resume once concerns about safety, protocol compliance, and data quality are addressed, and satisfy the sponsor, IRB and/or Food and Drug Administration (FDA).

13.2 QUALITY ASSURANCE AND QUALITY CONTROL

Each clinical site will perform internal quality management of study conduct, data and biological specimen collection, documentation and completion. An individualized quality management plan will be developed to describe a site's quality management.

Quality control (QC) procedures will be implemented beginning with the data entry system and data QC checks that will be run on the database will be generated. Any missing data or data anomalies will be communicated to the site(s) for clarification/resolution.

Following written Standard Operating Procedures (SOPs), the monitors will verify that the clinical trial is conducted and data are generated and biological specimens are collected, documented (recorded), and reported in compliance with the protocol, International Conference on Harmonisation Good Clinical Practice (ICH GCP), and applicable regulatory requirements (e.g., Good Laboratory Practices (GLP), Good Manufacturing Practices (GMP).

The investigational site will provide direct access to all trial related sites, source data/documents, and reports for the purpose of monitoring and auditing by the sponsor, and inspection by local and regulatory authorities.

13.3 CONFLICT OF INTEREST POLICY

The independence of this study from any actual or perceived influence, such as by the pharmaceutical industry, is critical. Therefore, any actual conflict of interest of persons who have a role in the design, conduct, analysis, publication, or any aspect of this trial will be disclosed and managed. Furthermore, persons who have a perceived conflict of interest will be required to have such conflicts managed in a way that is appropriate to their participation in the design and conduct of this trial. The study leadership in conjunction with the National Cancer Institute has established policies and procedures for all study group members to disclose all conflicts of interest and will establish a mechanism for the management of all reported dualities of interest.

14 PHARMACEUTICAL AND INVESTIGATIONAL DEVICE INFORMATION

14.1 PDS0101 (IND # 019267)

14.1.1 Source

PDS0101 will be provided by PDS Biotechnology through a CRADA.

14.1.2 Acquisition and Accountability

R-DOTAP and HPVmix will be provided to the clinical trial site by the manufacturer. The investigator or designee (e.g., pharmacist) will maintain an ongoing inventory of the investigational product supply according to standard site procedures. The investigational product will be dispensed at the direction of an investigator for administration to a study participant enrolled on the clinical trial. Disposal of expired or unused product will be returned to the manufacturer or disposed of according to standard site procedures based on agreement between the manufacturer and the site.

14.1.3 Toxicity

In 2014-2016, a Phase I/IIA human clinical trial was performed at 3 sites in the US in 12 subjects with high-risk HPV infection and biopsy-proven CIN1 to study the safety and immunogenicity of PDS0101. Administration of PDS0101 was well tolerated. The majority of AEs were due to administration site reactions. Most administration site reactions were mild or moderate in severity. Some more severe administration site reactions including swelling and redness were reported in the 3- and 10-mg R-DOTAP cohorts. Most administration site reactions resolved the same day or

within a few days. Per the subject symptom diary, administration site reactions were more severe and of longer duration in subjects in the 10-mg R-DOTAP cohort. No clinically significant differences in the types and pattern of TEAEs were observed between Vaccinations 1, 2, or 3. No DLTs were observed; thus, the MTD was the 10-mg R-DOTAP dose. No serious adverse events (SAEs), study discontinuations due to adverse events, or deaths occurred. No clinically relevant abnormal hematology, blood chemistry, urinalysis, or physical findings were observed.

14.1.4 Formulation and Preparation

PDS0101 is a 2-vial formulation which is mixed 1:1 v/v just prior to administration.

Vial #1 (ImmunoMAPK-RDOTAP): is a sterile, turbid pharmaceutical suspension of liposomal nanoparticles composed of the pure R-enantiomer of the cationic (positively charged) lipid DOTAP (dioleoyl-trimethylammonium propane). The particle size is between 100-200nm. RDOTAP is a novel immune modulator which induces the production of important cytokines and chemokines and also enhances the cytolytic T-Lymphocyte response when combined with the antigenic peptides. ImmunoMAPK-RDOTAP is has been evaluated at 3 dose levels, 1.0, 3.0 and 10.0mg.

Vial #2 (HPVmix): is a sterile, clear and slightly viscous suspension consisting of 6 nononcogenic lipidated peptides selected from immunogenic regions of the human papillomavirus strain 16 E6 and E7 proteins. The peptides exist in a mixed micellar particulate state. The HPVmix formulation contains 20% by volume of dimethyl sulfoxide (DMSO). HPVmix is being evaluated at a single dose level of 2.7 mg.

The 1:1 mixture of R-DOTAP and HPVmix (antigens) yields a mixture of liposomes and peptide mixed micelles in 10% DMSO. The nanoparticulate vaccine induces antigen specific T-cell therapeutic responses.

14.1.5 Stability and Storage

ImmunoMAPK-RDOTAP (Vial #1) and HPV Mix (Vial #2)

Storage: Store at -70°C ± 10°C.

Shipping: Shipped on dry ice in insulated container. Always check the temperature monitoring device to ensure maintenance of required storage condition during shipment.

R-DOTAP Thawing: Place at room temperature for a minimum of 90 minutes (1.5 hours) prior to mixing with HPVmix.

HPVmix Thawing: Remove from freezer at least 60 minutes (1 hour) after removal of RDOTAP. Place at room temperature for a minimum of 30 minutes (0.5 hours) and a maximum of 90 minutes (1.5 hours) prior to mixing with RDOTAP.

Mixture: PDS0101 should not be administered if held in vial for more than 120 minutes (2 hours) at ambient temperature after mixing.

14.1.6 Administration Procedures

Subcutaneous administration in upper thigh(s)
Two separate 0.5mL injections of Vaccine
Total Dose: 3.0mg R-DOTAP/2.7 mg HPV Peptides

14.1.7 Incompatibilities

Not available.

14.2 M7824 (MSB0011359C) (IND # 019267)

14.2.1 Source

M7824 is manufactured and supplied for the trial by EMD Serono Research and Development Institute.

14.2.2 Acquisition and Accountability

M7824 will be provided to the clinical trial site by the manufacturer. The investigator or designee (e.g., pharmacist) will maintain an ongoing inventory of the investigational product supply according to standard site procedures. The investigational product will be dispensed at the direction of an investigator for administration to a study participant enrolled on the clinical trial. Disposal of expired or unused product will be returned to the manufacturer or disposed of according to standard site procedures based on agreement between the manufacturer and the site.

14.2.3 Toxicity

The immunoglobulin portion of M7824 molecule is identical to avelumab (Bavencio). Respective warnings and precautions for grade 2 or higher immune-mediated pneumonitis, immune-mediated colitis, immune-mediated endocrinopathies, immune-mediated hepatitis) and infusion reactions are included in the prescribing for Bavencio (bavencio .com). Patients will be pre-medicated to prophylax against infusions reactions. The following additionally significant immune-mediated adverse reactions have occurred in less than 1% of 1738 patients treated with BAVENCIO: myocarditis with fatal cases, myositis, psoriasis, arthritis, exfoliative dermatitis, erythema multiforme, pemphigoid, hypopituitarism, uveitis, Guillain-Barré syndrome, and systemic inflammatory response. The above irAEs are all considered an anticipated risk of treatment with M7824 and thus will not be considered DLTs.

In a phase 1, open-label 3+3 dose-escalation study of M7824 in 16 patients, 3 patients experienced grade 3 drug-related adverse events including skin infection secondary to grade 2 bullous pemphigoid, lipase increased, and colitis with associated anemia. There were no grade 4 – 5 treatment related adverse events. Please see table below for details.

Treatment-related adverse events

	3 mg/kg (n = 3)		*10 mg/kg (n = 3)*		*20 mg/kg (n = 7)*		*Total (n = 16)*	
	Any Grade	Grade 3	Any Grade	Grade 3	Any Grade	Grade 3	Any Grade	Grade 3
**Patients with any event****	**2 (66.7)**	**1 (33.3)**	**1 (33.3)**	**0 (0.0)**	**4 (57.1)**	**2 (28.6)**	**7 (43.8)**	**3 (18.8)**
Anemia					1 (14.3)	1 (14.3)	1 (6.3)	1 (6.3)
Bullous pemphigoid	1 (33.3)						1 (6.3)	
Colitis					1 (14.3)	1 (14.3)	1 (6.3)	1 (6.3)
Dermatitis acneiform			1 (33.3)				1 (6.3)	
Dyspnea exertional***					1 (14.3)		1 (6.3)	
Hyperthyroidism					1 (14.3)		1 (6.3)	
Hypophosphatemia					1 (14.3)		1 (6.3)	
Hypothyroidism			1 (33.3)		1 (14.3)		2 (12.5)	
Infusion-related reaction					1 (14.3)		1 (6.3)	
Keratoacanthoma					1 (14.3)		1 (6.3)	
Lipase increase					1 (14.3)	1 (14.3)	1 (6.3)	1 (6.3)
Nausea	1 (33.3)						1 (6.3)	
Pruritus	1 (33.3)						1 (6.3)	
Rash maculo-papular	1 (33.3)		1 (33.3)				2 (12.5)	
Skin infection	1 (33.3)	1 (33.3)					1 (6.3)	1 (6.3)
Vomiting	1 (33.3)						1 (6.3)	

**There were no treatment-related AEs in the 3 patients treated with 1 mg/kg M7824.

***The differential for this dyspnea was pneumonitis vs. lymphangitic spread of disease (disease progression).

As of August 2017, > 500 patients have been treated with M7824 across multiple solid tumor expansion cohorts. The safety profile is consistent with other monotherapy checkpoint inhibitors, with the exception of keratoacanthomas and cutaneous squamous cell carcinomas which have occurred in approximately 3-5% of patients, and are well managed with surgical excision. These lesions have not been a criterion for treatment discontinuation, but thus far have all spontaneously regressed following treatment discontinuation.

In addition, after discussion among NCI investigators on multiple protocols using M7824, multiple bleeding events ranging from low grade gingival bleeding and epistaxis to more serious hemoptysis, GI bleeding, and hematuria have been observed. Some of these events can be attributed to bleeding events related to cancer. However, there remains the possibility that M7824 may increase the overall risk of bleeding in ways that may not be directly related to direct tumor bleeding or inflammatory bleeding events described with checkpoint inhibitors like M7824. It is hypothesized that this possible increased bleeding risk may be due to TGF beta inhibition which has an effect on angiogenesis. Accordingly, patients will be notified of the same possible risk in the informed consent document for this study (e.g., gum bleeding, nose bleeds, coughing up blood, blood in their urine, or blood in the stool).

14.2.4 Formulation and Preparation

M7824 is provided as a sterile liquid formulation and packaged at a 10 mg/mL concentration in USP/ Ph Eur type I 50R vials that are filled with drug product solution to allow an extractable volume of 60 mL (600 mg/60 mL). The vials are closed with rubber stoppers in serum format complying with USP and Ph Eur with an aluminum crimp seal closure. Each single-use vial contains 600mg of M7824, formulated as 10mg/mL of active, 6% (w/v) Trehalose, 40 mM NaCl, 5 mM Methionine, 0.05% (w/v) Tween 20, 10 mM L-Histidine at pH 5.5.

The liquid formulation is diluted directly with 0.9% sodium chloride solution for injection. The estimated volumes of delivery are anticipated to be no more than 250mL. The verified concentration range in the infusion solution is 0.16 mg/mL to 9.6 mg/mL.

14.2.5 Stability and Storage

M7824 must be stored at 2℃ to 8℃ until use. Product stored at room temperature for extended periods of time might be subject to degradation. M7824 must not be frozen. Rough shaking of the reconstituted solution must be avoided.

The chemical and physical in-use stability for the infusion solution of M7824 in 0.9% sodium chloride for injection has been demonstrated for a total of 72 hours at room temperature; however, from a microbiological point of view, the diluted solution should be used immediately and is not intended to be stored unless dilution has taken place in controlled and validated aseptic conditions. No other drugs should be added to the infusion containers containing M7824. See Manual of Preparation of approved ancillary supplies.

14.2.6 Administration Procedures

See Section **3.2**.

14.2.7 Incompatibilities

Not available.

14.3 NHS-IL12 (IND # 019267)

14.3.1 Source

NHS-IL12 is manufactured and supplied by EMD Serono.

14.3.2 Acquisition and Accountability

NHS-IL12 will be provided to the clinical trial site by the manufacturer. The investigator or designee (e.g., pharmacist) will maintain an ongoing inventory of the investigational product supply according to standard site procedures. The investigational product will be dispensed at the direction of an investigator for administration to a study participant enrolled on the clinical trial. Disposal of expired or unused product will be returned to the manufacturer or disposed of according to standard site procedures based on agreement between the manufacturer and the site.

14.3.3 Toxicity

Subjects (n=59) were treated subcutaneously with NHS-IL12 in a single ascending dose cohort followed by a multiple ascending dose cohort (n=37 with every 4-week dosing). The primary objective of this trial was to determine MTD as defined by the number of DLTs. None of the subjects treated with single or multiple doses up to 12.0 µg/kg experienced a DLT. At 16.8 µg/kg, 1/6 subjects had a DLT (grade 3 increase in alanine transaminase [ALT]). At 21.8 µg/kg, 2/6 subjects had a DLT (grade 3 increase in aspartate transaminase [AST] and ALT; grade 3 increase in lipase without clinical signs of pancreatitis). MTD was 16.8 µg/kg. The most frequently observed treatment-related adverse event (TRAE) was decreased lymphocyte count (27/59 subjects; 45.8%). Other TRAEs included decreased white blood cells (WBCs) (24/59; 40.7%), fever and elevated AST (21 each; 35.6%), elevated ALT (20; 3.9%), and anemia and flu-like symptoms (18 each; 30.5%) (Table 2; Supplemental Figure 5). Among the cohort receiving 16.8 µg/kg, the most frequently reported TRAEs were elevated AST (75%), decreased WBCs and elevated ALT (68.8% each), and decreased lymphocyte count and fever (62.5% each). At least one grade ≥ 3 TRAE was seen in 12/59 subjects (20.3%). These included decreased lymphocyte count (5; 8.5%), decreased neutrophil count (4; 6.8%), elevated ALT (3; 5.1%), decreased WBCs (2; 3.4%), and hypokalemia, hyperhidrosis, elevated alkaline phosphatase, AST, and lipase (1 each; 1.7%). All grade ≥ 3 TRAEs were transient; only hyperhidrosis was symptomatic. One grade 4 TRAE was observed (asymptomatic decreased lymphocyte count); no grade 5 TRAE was observed.

14.3.4 Formulation and Preparation

NHS-IL12 is formulated as a 1.5 mg/mL solution and is supplied by the Sponsor in single-use glass vials with a rubber stopper and sealed with an aluminum Flip Off® crimp seal closure.

The contents of the NHS-IL12 vials are sterile and nonpyrogenic, and do not contain bacteriostatic preservatives. Any spills that occur should be cleaned up using the facility's standard cleanup procedures for biologic products.

Rough shaking of the solution must be avoided.

NHS-IL12 drug product is ready-to-use. However, for doses below 300 µg, NHS-IL12 drug product must be diluted with 0.9% saline solution (sodium chloride injection). Detailed information on medical devices to be used for the preparation and administration of NHS-IL12 will be provided in the Manual of Operations.

Any unused portion of the solution should be discarded in biohazard waste disposal with final disposal by accepted local and national standards of incineration.

14.3.5 Stability and Storage

NHS-IL12 drug product must be stored at 2°C to 8°C until use, with a temperature log maintained daily. All medication boxes supplied to the study site must be stored carefully, safely, and separately from other drugs.

The chemical and physical in-use stability of NHS-IL12 diluted and undiluted solution in syringes has been demonstrated for 24 hours at 25°C. However, to reduce the risk of microbial contamination, the solution should be used immediately unless medication preparation has been performed in controlled and validated aseptic conditions. Rough shaking of the solution must be avoided.

14.3.6 Administration Procedures

NHS-IL12 is administered subcutaneously. It can be given in the arm or leg. Where feasible it should be given in a different limb than the vaccine.

14.3.7 Incompatibilities

Not available.

15 REFERENCES

1. Viens LJ, Henley SJ, Watson M, Markowitz LE, Thomas CC, Thompson TD, Razzaghi H, Saraiya M: **Human Papillomavirus-Associated Cancers - United States, 2008-2012**. *MMWR Morb Mortal Wkly Rep* 2016, **65**(26):661-666.

2. Long HJ, 3rd, Bundy BN, Grendys EC, Jr., Benda JA, McMeekin DS, Sorosky J, Miller DS, Eaton LA, Fiorica JV, Gynecologic Oncology Group S: **Randomized phase III trial of cisplatin with or without topotecan in carcinoma of the uterine cervix: a Gynecologic Oncology Group Study**. *J Clin Oncol* 2005, **23**(21):4626-4633.

3. Vermorken JB, Mesia R, Rivera F, Remenar E, Kawecki A, Rottey S, Erfan J, Zabolotnyy D, Kienzer HR, Cupissol D *et al*: **Platinum-based chemotherapy plus cetuximab in head and neck cancer**. *N Engl J Med* 2008, **359**(11):1116-1127.

4. Long HJ, 3rd: **Management of metastatic cervical cancer: review of the literature**. *J Clin Oncol* 2007, **25**(20):2966-2974.

5. Eng C, Chang GJ, You YN, Das P, Rodriguez-Bigas M, Xing Y, Vauthey JN, Rogers JE, Ohinata A, Pathak P *et al*: **The role of systemic chemotherapy and multidisciplinary management in improving the overall survival of patients with metastatic squamous cell carcinoma of the anal canal**. *Oncotarget* 2014, **5**(22):11133-11142.

6. Monk BJ, Sill MW, McMeekin DS, Cohn DE, Ramondetta LM, Boardman CH, Benda J, Cella D: **Phase III trial of four cisplatin-containing doublet combinations in stage IVB, recurrent, or persistent cervical carcinoma: a Gynecologic Oncology Group study**. *J Clin Oncol* 2009, **27**(28):4649-4655.

7. Tewari KS, Sill MW, Long HJ, 3rd, Penson RT, Huang H, Ramondetta LM, Landrum LM, Oaknin A, Reid TJ, Leitao MM *et al*: **Improved survival with bevacizumab in advanced cervical cancer**. *N Engl J Med* 2014, **370**(8):734-743.

8. Monk BJ, Sill MW, Burger RA, Gray HJ, Buekers TE, Roman LD: **Phase II trial of bevacizumab in the treatment of persistent or recurrent squamous cell carcinoma of the cervix: a gynecologic oncology group study**. *J Clin Oncol* 2009, **27**(7):1069-1074.

9. van Herpen CM, van der Voort R, van der Laak JA, Klasen IS, de Graaf AO, van Kempen LC, de Vries IJ, Boer TD, Dolstra H, Torensma R *et al*: **Intratumoral rhIL-12 administration in head and neck squamous cell carcinoma patients induces B cell activation**. *Int J Cancer* 2008, **123**(10):2354-2361.

10. Grohmann U, Belladonna ML, Bianchi R, Orabona C, Ayroldi E, Fioretti MC, Puccetti P: **IL-12 acts directly on DC to promote nuclear localization of NF-kappaB and primes DC for IL-12 production**. *Immunity* 1998, **9**(3):315-323.

11. Del Vecchio M, Bajetta E, Canova S, Lotze MT, Wesa A, Parmiani G, Anichini A: **Interleukin-12: biological properties and clinical application**. *Clin Cancer Res* 2007, **13**(16):4677-4685.

12. Massarelli E, William W, Johnson F, Kies M, Ferrarotto R, Guo M, Feng L, Lee JJ, Tran H, Kim YU *et al*: **Combining Immune Checkpoint Blockade and Tumor-Specific**

Vaccine for Patients With Incurable Human Papillomavirus 16-Related Cancer: A Phase 2 Clinical Trial. *JAMA Oncol* 2019, **5**(1):67-73.

13. Lonez C, Vandenbranden M, Ruysschaert JM: **Cationic liposomal lipids: from gene carriers to cell signaling**. *Prog Lipid Res* 2008, **47**(5):340-347.

14. Boyerinas B, Jochems C, Fantini M, Heery CR, Gulley JL, Tsang KY, Schlom J: **Antibody-Dependent Cellular Cytotoxicity Activity of a Novel Anti-PD-L1 Antibody Avelumab (MSB0010718C) on Human Tumor Cells**. *Cancer Immunol Res* 2015, **3**(10):1148-1157.

15. Heery CR, O'Sullivan-Coyne G, Madan RA, Cordes L, Rajan A, Rauckhorst M, Lamping E, Oyelakin I, Marte JL, Lepone LM *et al*: **Avelumab for metastatic or locally advanced previously treated solid tumours (JAVELIN Solid Tumor): a phase 1a, multicohort, dose-escalation trial**. *Lancet Oncol* 2017, **18**(5):587-598.

16. Donahue RN, Lepone LM, Grenga I, Jochems C, Fantini M, Madan RA, Heery CR, Gulley JL, Schlom J: **Analyses of the peripheral immunome following multiple administrations of avelumab, a human IgG1 anti-PD-L1 monoclonal antibody**. *J Immunother Cancer* 2017, **5**:20.

17. Blobe GC, Schiemann WP, Lodish HF: **Role of transforming growth factor beta in human disease**. *N Engl J Med* 2000, **342**(18):1350-1358.

18. Dumont N, Arteaga CL: **Transforming growth factor-beta and breast cancer: Tumor promoting effects of transforming growth factor-beta**. *Breast Cancer Res* 2000, **2**(2):125-132.

19. Formenti SC, Lee P, Adams S, Goldberg JD, Li X, Xie MW, Ratikan JA, Felix C, Hwang L, Faull KF *et al*: **Focal Irradiation and Systemic TGFbeta Blockade in Metastatic Breast Cancer**. *Clin Cancer Res* 2018, **24**(11):2493-2504.

20. Ivanovic V, Melman A, Davis-Joseph B, Valcic M, Geliebter J: **Elevated plasma levels of TGF-beta 1 in patients with invasive prostate cancer**. *Nat Med* 1995, **1**(4):282-284.

21. Shen M, Tsai Y, Zhu R, Keng PC, Chen Y, Chen Y, Lee SO: **FASN-TGF-beta1-PD-L1 axis contributes to the development of resistance to NK cell cytotoxicity of cisplatin-resistant lung cancer cells**. *Biochim Biophys Acta Mol Cell Biol Lipids* 2018, **1863**(3):313-322.

22. Tsushima H, Kawata S, Tamura S, Ito N, Shirai Y, Kiso S, Imai Y, Shimomukai H, Nomura Y, Matsuda Y *et al*: **High levels of transforming growth factor beta 1 in patients with colorectal cancer: association with disease progression**. *Gastroenterology* 1996, **110**(2):375-382.

23. Terabe M, Robertson FC, Clark K, De Ravin E, Bloom A, Venzon DJ, Kato S, Mirza A, Berzofsky JA: **Blockade of only TGF-beta 1 and 2 is sufficient to enhance the efficacy of vaccine and PD-1 checkpoint blockade immunotherapy**. *Oncoimmunology* 2017, **6**(5):e1308616.

24. Vanpouille-Box C, Formenti SC: **Dual Transforming Growth Factor-beta and Programmed Death-1 Blockade: A Strategy for Immune-Excluded Tumors?** *Trends Immunol* 2018, **39**(6):435-437.

25. Grenga I, Donahue RN, Gargulak ML, Lepone LM, Roselli M, Bilusic M, Schlom J: **Anti-PD-L1/TGFbetaR2 (M7824) fusion protein induces immunogenic modulation of human urothelial carcinoma cell lines, rendering them more susceptible to immune-mediated recognition and lysis**. *Urol Oncol* 2018, **36**(3):93 e91-93 e11.

26. Jochems C, Tritsch SR, Pellom ST, Su Z, Soon-Shiong P, Wong HC, Gulley JL, Schlom J: **Analyses of functions of an anti-PD-L1/TGFbetaR2 bispecific fusion protein (M7824)**. *Oncotarget* 2017, **8**(43):75217-75231.

27. Knudson KM, Hicks KC, Luo X, Chen JQ, Schlom J, Gameiro SR: **M7824, a novel bifunctional anti-PD-L1/TGFbeta Trap fusion protein, promotes anti-tumor efficacy as monotherapy and in combination with vaccine**. *Oncoimmunology* 2018, **7**(5):e1426519.

28. Lan Y, Zhang D, Xu C, Hance KW, Marelli B, Qi J, Yu H, Qin G, Sircar A, Hernandez VM *et al*: **Enhanced preclinical antitumor activity of M7824, a bifunctional fusion protein simultaneously targeting PD-L1 and TGF-beta**. *Sci Transl Med* 2018, **10**(424).

29. Ravi R, Noonan KA, Pham V, Bedi R, Zhavoronkov A, Ozerov IV, Makarev E, A VA, Wysocki PT, Mehra R *et al*: **Bifunctional immune checkpoint-targeted antibody-ligand traps that simultaneously disable TGFbeta enhance the efficacy of cancer immunotherapy**. *Nat Commun* 2018, **9**(1):741.

30. David JM, Dominguez C, McCampbell KK, Gulley JL, Schlom J, Palena C: **A novel bifunctional anti-PD-L1/TGF-beta Trap fusion protein (M7824) efficiently reverts mesenchymalization of human lung cancer cells**. *Oncoimmunology* 2017, **6**(10):e1349589.

31. Strauss J, Heery C, Schlom J, Madan RA, Cao L, Kang Z, Lamping E, Marte JL, Donahue RN, Grenga I *et al*: **Phase 1 trial of M7824 (MSB0011359C), a bifunctional fusion protein targeting PD-L1 and TGF-beta, in advanced solid tumors**. *Clin Cancer Res* 2018.

32. Fallon J, Tighe R, Kradjian G, Guzman W, Bernhardt A, Neuteboom B, Lan Y, Sabzevari H, Schlom J, Greiner JW: **The immunocytokine NHS-IL12 as a potential cancer therapeutic**. *Oncotarget* 2014, **5**(7):1869-1884.

33. Strauss J, Heery CR, Kim JW, Jochems C, Donahue RN, Montgomery AS, McMahon S, Lamping E, Marte JL, Madan RA *et al*: **First-in-Human Phase I Trial of a Tumor-Targeted Cytokine (NHS-IL12) in Subjects with Metastatic Solid Tumors**. *Clin Cancer Res* 2019, **25**(1):99-109.

34. Lepone LM, Donahue RN, Grenga I, Metenou S, Richards J, Heery CR, Madan RA, Gulley JL, Schlom J: **Analyses of 123 Peripheral Human Immune Cell Subsets: Defining Differences with Age and between Healthy Donors and Cancer Patients Not Detected in Analysis of Standard Immune Cell Types**. *J Circ Biomark* 2016, **5**:5.

35. Eisenhauer EA, Therasse P, Bogaerts J, Schwartz LH, Sargent D, Ford R, Dancey J, Arbuck S, Gwyther S, Mooney M *et al*: **New response evaluation criteria in solid tumours: revised RECIST guideline (version 1.1)**. *Eur J Cancer* 2009, **45**(2):228-247.

36. Seymour L, Bogaerts J, Perrone A, Ford R, Schwartz LH, Mandrekar S, Lin NU, Litiere S, Dancey J, Chen A *et al*: **iRECIST: guidelines for response criteria for use in trials testing immunotherapeutics**. *Lancet Oncol* 2017, **18**(3):e143-e152.

37. Zhestianikov VD, Savel'eva GE, Kalinin VL: **[The adaptive response to mitomycin C exposure in the hyper-radioresistant mutant Escherichia coli Gamr444].**

Adaptivnyi otvet na vozdeistvie mitomitsinom C u giperradiorezistentnogo mutanta Escherichia coli Gamr444. *Tsitologiia* 1991, **33**(1):88-96.

16 APPENDICIES

16.1 APPENDIX A PERFORMANCE STATUS CRITERIA

ECOG Performance Status Scale		Karnofsky Performance Scale	
Grade	Descriptions	Percent	Description
0	Normal activity. Fully active, able to carry on all pre-disease performance without restriction.	100	Normal, no complaints, no evidence of disease.
		90	Able to carry on normal activity; minor signs or symptoms of disease.
1	Symptoms, but ambulatory. Restricted in physically strenuous activity, but ambulatory and able to carry out work of a light or sedentary nature (e.g., light housework, office work).	80	Normal activity with effort; some signs or symptoms of disease.
		70	Cares for self, unable to carry on normal activity or to do active work.
2	In bed <50% of the time. Ambulatory and capable of all self-care, but unable to carry out any work activities. Up and about more than 50% of waking hours.	60	Requires occasional assistance, but is able to care for most of his/her needs.
		50	Requires considerable assistance and frequent medical care.
3	In bed >50% of the time. Capable of only limited self-care, confined to bed or chair more than 50% of waking hours.	40	Disabled, requires special care and assistance.
		30	Severely disabled, hospitalization indicated. Death not imminent.
4	100% bedridden. Completely disabled. Cannot carry on any self-care. Totally confined to bed or chair.	20	Very sick, hospitalization indicated. Death not imminent.
		10	Moribund, fatal processes progressing rapidly.
5	Dead.	0	Dead.

17 INVESTIGATOR AGREEMENT

I have received and reviewed the Investigator Brochures for PDS0101, M7824, and NHS-IL12.

I have read this protocol and agree that the study is ethical.

I agree to conduct the study as outlined and in accordance with all applicable regulations and guidelines.

I agree to maintain the confidentiality of all information received or developed in connection with this protocol.

Protocol Title: **Phase I/II Trial of Combination Immunotherapy in Subjects with Advanced HPV Associated Malignancies**

Protocol Version Date: **February 03, 2020**

____ ____________________

Signature of Principal Investigator Date

Julius Strauss, MD

Name of Principal Investigator (printed or typed)

Amendment A February 26, 2020

Protocol Changes:

1) Header: The version date has been updated.
2) Title Page:
a. The CC protocol number has been added.
b. The version date and amendment letter have been updated.
c. The NCT Number was added.
3) The Table of Contents was updated.
4) Precis, Objectives and Section 1.1.1 Primary Objective: The primary objective is clarified to indicate that the population of interest is subjects with checkpoint naïve or checkpoint refractory advanced HPV associated malignancies.
5) Precis, Eligibility and Section 2.1.2 Exclusion Criteria: Exclusion of prior checkpoint inhibitor use was removed to allow patients with checkpoint refractory disease to enroll.
6) Section 1.1.3 Exploratory Objectives: An additional exploratory endpoint was added to evaluate the response rate among checkpoint refractory patients.
7) Section 1.2.2.1 HPV Vaccines: Rationale for allowing patients with checkpoint refractory disease to enroll was added.
8) Section 2.1.2.2 Exclusion Criterion: An additional exclusion criterion was added for patients with a known intolerance to or life threatening side effects resulting from prior checkpoint inhibitor therapy.
9) Section 2.4 Baseline Evaluation and Section 3.5 Study Calendar: The free T4 test was corrected to 'reflex free T4' to clarify that the test will be run only when there is an abnormal TSH result.
10) Section 3.1 Study Design: A bullet was added to clarify that response rate among checkpoint refractory patients will be an exploratory objective and not a primary objective as the response rate for checkpoint naïve patients.
11) Section 3.4 Dose Modification: Clarification that patients who require a dose modification or skipped does within the DLT period for reasons other than a DLT will be replaced.
12) Section 3.7.2 Off-Study Criteria: Screen failure was removed as an off-study criterion since subjects are not screened on this protocol.
13) Section 5.3 Sample Storage, Tracking, and Disposition: This section was updated to the correct procedure.
14) Section 6.1 Data Collection: 'Intervention' was updated to 'drug administration' to clarify the practice of documenting AEs once the first administration of study drug.
15) Section 10.2 Sample Size Determination: The accrual ceiling is increased by 5 patients to allow for inevaluable patients for the primary objective.
16) Section 12.5.1 Telephone Re-Consent Procedure and Section 14 Pharmaceutical Information:
These sections were updated to comply with current OHSRP/IRBO and CCR policies and procedures.
17) Section 17 Investigator Agreement: This section was removed as it is intended for use in muti-site studies and this is a single site study.

Amendment B July 08, 2020

Protocol Changes:

1) Header: The version date has been updated.
2) Title Page: The version date and amendment letter have been updated.
3) Precis, Design and Section 3.1.1 Dose Limiting Toxicity: The DLT language was expanded to clarify that it is possible for HPV patients to have a grade 3 AE with M7824 alone and the benefit of this drug outweighs the risk.
4) The Table of Contents was updated.
5) Section 1.2.2.1 HPV Vaccine: Figure 2 was updated for demonstration of immunogenicity across multiple HLA types and clinical benefit from the phase I study and a citation was added.
6) Section 2.2.2 Screening activities performed after a consent for screening has been signed, Section 2.3.1 Screen Failure, and Section 3.7.2 Off-Study Criteria: These sections were updated/added to clarify that patients could be consented on this protocol if the required screening procedures are not approved on the screening protocol. Patients may be lost to screen failure with the possibility of screening on protocol.
7) Section 2.2.2 Screening activities performed after a consent for screening has been signed and Section 3.5 Study Calendar: Screening activities and protocol procedures have been updated to reflect the scans that are for research purposes and remove clinically indicated only scans or add them to the footnotes (Study Calendar) for clarification.
8) Section 2.3 Participant Registration and Status Update: The section now refers to the CCR SOP for Participant Registration and Status Updates for the most up to date procedures.
9) Section 3.1.1 Dose Limiting Toxicity and Section 3.4 Dose Modification: The DLT discontinuation language was updated for correctness.
10) Section 3.2 Drug Administration and Section 14.1.5 Administration Procedures: Administration language for PDS0101 was updated for clarity. In addition, the specific dose of NHS-IL-12 was removed in favor of reference to the newly added dose levels.
11) Section 3.5 Study Calendar, Footnote 8: Mineral panel was added to the Biochemical profile; it was accidentally omitted in previous versions.
12) Section 3.7.4 Off Protocol Therapy and Off-Study Procedure: This section was removed per the changes in Section 2.3 Participant Registration and Status Update.
13) Section 5.2.3 Management of Results: This section was incorrectly removed during a revision; it has been added back in since incidental findings may be returned to the patient.
14) Section 10.2 Sample Size Determination: The expected enrollment numbers were updated and the accrual ceiling was increased to allow for replacement of HPV negative patients and screen failures.
15) Section 12.4.1 Risks: Clarification that not all study procedures are associated with risk and that those which are will be described in separate sections.
16) Section 12.4.1.2 Risks of Exposure to Ionizing Radiation: This section was updated to include all CTs scans, total radiation exposure, and risk associated with this research protocol according to current guidelines.
17) Section 12.4.1.3 Additional Risks of CT scans: The section was created to address the non-radiation risks of CT scans.
18) Section 12.5 Consent Process and Documentation: This section was updated to provide more

detail and to comply with current OHSRP/IRBO and CCR policies and procedures.

19) Section 12.5.1 Telephone Re-Consent Procedure: The information in this section is now included in the updated language in Section 12.5 Consent Process and Documentation.

Amendment C September 10, 2020

Protocol Changes:
1) Header: The version date has been updated.
2) Title Page:
a. The version date and amendment letter have been updated.
b. The CC protocol number was adjusted to the NIH Protocol number
3) Section 1.2.2.1 NHS-IL12 Immunocytokine (M9241): Added notation on prior available data for the alternate dosing schedule of every 2 weeks.
4) Section 3.4.2 Dose Delay and Section 3.4.3 Dose Modification: Clarification on the restarting schedule was added and an alternative dosing schedule was provided for NHS-IL12.

Amendment December 11, 2020

Protocol Changes:

1) Header and Title Page: The version date has been updated.
2) Throughout the protocol, the term ‘patient(s)’ has been updated to the term ‘participant(s)’ where appropriate.
3) The Table of Contents was updated.
4) 1.1.3 Exploratory Objectives: A new exploratory objective has been added to assess response to therapy by HLA type.
5) Section 1.2.3 Safety and Efficacy of I/O Combinations in Humans: The maximum dose of NHS-IL12 was reduced to 8 mcg/kg with rationale provided.
6) Section 2.1.1.10: A new inclusion criterion was added to include the ability to sign an informed consent document as patients unable to consent are excluded from enrolling on this protocol.
7) Section 2.2.2 Screening activities performed after a consent for screening has been signed: HLA typing was added to assess the new exploratory objective.
8) Section 3.1 Study Design, Figure 19: This figure was updated to reflect the change in maximum doses of NHS-IL12 from 16 to 8 mcg/kg.
9) Section 3.2 Drug Administration, NHS-IL12:
a. The maximum dose of NHS-IL12 was reduced to 8 mcg/kg.
b. The timeline for collecting subject weight prior to drug administration was extended.
10) Section 3.4.1 Discontinuation: The statement, ‘without medical intervention’ was removed.
11) Section 3.4.3 Dose Modification: This section was updated align with the change in maximum NHS-IL12 dose.
12) Section 3.5 Study Calendar: HLA typing was added as a procedure.
13) Section 3.6.3 Reimbursement: This section was updated to comply with current OHSRP/IRBO and CCR policies and procedures.
14) Section 8.1.2 Adverse Event of Special Interest (AESI): This section was added per the request of OSRO.
15) Section 10.2 Sample Size Determination: The accrual ceiling was increased to 47 patients.
16) Section 12.3 Participation of Subjects Unable to Give Consent: This section was updated to comply with current OHSRP/IRBO and CCR policies and procedures.
17) Section 12.5 Consent Process and Documentation: This section was updated to comply with current OHSRP/IRBO and CCR policies and procedures.
18) Section 12.5.1 Consent Process for Adults Who Lack Capacity to Consent to Research Participation: This section was added to comply with current OHSRP/IRBO and CCR policies and procedures.
19) Section 13.4 Confidentiality and Privacy: This section was updated to comply with current OHSRP/IRBO and CCR policies and procedures.
20) Section 14.3.5 Administration Procedures: The administration of NHS-IL12 was updated to include the abdomen as a potential injection site.

Amendment January 23, 2021

Protocol Changes:

1) Header and Title Page: The version date has been updated.
2) Precis, Design: A statement was added to address the addition of a new cohort for cervical cancer patients with prior radiation and boost brachytherapy.
3) The Table of Contents was updated.
4) Statement of Compliance: This section was updated to name the body making the consent determination of need for consent.
5) Section 1.1.2 Secondary Objective: A new objective was added to address cervical cancer patients who previously had brachytherapy boost as they appear to be prone to gross hematuria with the current dosing regimen. Section 1.1.3 Exploratory Objectives: An objective for the analysis of response to lower doses of study drug has been added.
6) Section 2.3.2 Treatment Assignment Procedures:
a. Cohort 2 and Arm 2 were added to separate out cervical cancer patients (Cohort 2) and examine the reduced doses (Arm 2).
b. The arm assignments were updated to address the new Cohort and Arm.
7) Section 3.1 Study Design: Information was added to address the increase in accrual ceiling.
8) Section 3.1 Study Design and Section 3.2 Drug Administration: It is noted that lower starting doses of M7824 and/or NHSIL12 may be used.
9) Section 3.4.3 Dose Modification: Updated to allow for dose reductions of M7824 and NHS-IL12 particularly in participants with cervical cancer who have received prior pelvic radiation.
10) Section 5 Correlative Studies for Research/Pharmacokinetic Studies and 5.1 Biospecimen Collection: These headings were updated for correctness and clarity.
11) Section 10.1 Statistical Hypothesis: Information was added to address the new Cohort 2.
12) Section 10.2: Sample Size Determination: Information was added to address the new Cohort 2 and the accrual ceiling was increased to 56 participants.
13) Section 10.4.2 Analysis of Primary Endpoints: It is clarified which subjects will be analyzed for the primary endpoint.
14) Section 10.4.4 Safety Analysis: Information was added to address the new Cohort 2.
15) 10.4.8 Exploratory Analyses: A new section was added (10.4.8.2) that includes analysis details for the new exploratory objective.
16) Section 12.5 Consent Process and Documentation: Assent was removed from the heading as there is no assent for this study.
17) Section 13.1 Study Discontinuation and Closure: A minor update to the language was made for clarity.
18) Section 13.4 Confidentiality and Privacy: An edit was made for correctness.
19) Section 14.3.3 Formulation and Preparation: This section was updated to reflect updated pharmacy preparation information for NHS-IL12.

Amendment April 26, 2021

Protocol Changes:
1) Header and Title Page: The version date has been updated.
2) Title Page: The supplier of the study drugs was added to the Investigational Agents table to provide clarification for the pharmacy as to who is responsible to provide/procure the study drugs.
3) Precis, Design: Updated to reflect changes in the protocol in Section 10.2.
4) Section 3.4.3 Dose Modification: The stopping rule was revised based on the SMC recommendation that 'stopping riles based on toxicity other than death should be included in the protocol.'
5) Section 6.1 Data Collection: The primary electronic system for the study data that complies with security standards is noted in the first sentence. This text is removed as primarily duplicative and not all drives or "alternative" sites are assessed for compliance.
6) Section 8.3 Reporting Serious Adverse Events: Location of sponsor reporting form updated.
7) Section 8.6 Reporting Pregnancy: Reference to new OSRO reporting forms for pregnancy were added.
8) Section 10.2 Sample Size Determination: The section was updated to clarify only 'checkpoint naïve patients' will be counted in the primary statistical analysis.
9) Section 10.4.2 Analysis of the Primary Endpoints: Updated to clarify checkpoint naïve subjects.
10) Section 12.3 Participation of Subjects Unable to Give Consent: Reference to the section for consent procedures for those who lose capacity was added.
11) Section 12.5 Consent Process and Documentation: Reference to policy M20-1 was added.

Amendment July 04, 2021

Protocol Changes:
1) Header and Title Page: The version date has been updated.
2) Section 2.1.2.8 Exclusion Criterion: A new exclusion criterion with added to address the situation of patients that do not accept blood products.
3) Section 4.1 The Following Treatments Should not be Administered During the Trial: Approved COVID vaccines are allowed per the updated safety related language for M7824.
4) Section 5.1 Biospecimen Collection and Section 5.1.3 Analyses of Tumor Tissue for Immune Markers: The laboratory for analysis has been updated from Dr. Houssein Sater's lab to the GMB TIME Laboratory.
5) Section 8.1.7 AESI: This section was added to reflect mucosal bleeding as an AESI requested by the Sponsor.
6) Section 12.1 Rationale for Subject Selection: Added rationale for exclusion of patients that do not accept blood products.
7) Section 12.5 Consent Process and Documentation: Information about electronic documentation of consent was added.
8) Section 14.2.2 Toxicity: An additional adverse event that has been noted in patients on M7824 was added to the toxicity information.

Amendment October 20, 2021

Protocol Changes:

1) Header and Title Page: The version date has been updated.
2) Section 1.1.2 Secondary Objectives: Removed specific starting doses in the objective. The actual doses are described within other sections of the protocol.
3) Section 1.2.3 Safety and Efficacy of I/O Combinations in Humans: The justification/rationale for the change in the NHS-IL12 dosing schedule was added.
4) Section 2.1.1.3 Inclusion Criteria: A criterion was modified to further clarify lines of prior therapy for patients with advanced HPV related cancer being enrolled on this protocol.
5) Section 3.1 Study Design, Figure 19: The figure was updated to reflect the change in NHSIL12 dosing schedule and refer to Section 3.4.3.
6) Section 3.2.3 NHS-IL12: The starting does/dosing schedule for NHS-IL12 was changed.
7) Section 3.4.3 Dose Modification, NHS-IL12, Section 10.1 Statistical Hypothesis, and Section 10.2 Sample Size Determination: The starting dose for NHS-IL12 was updated to 16.8 mcg/kg.
8) Section 3.4.3 Dose Modification, NHS-IL12: The dose modification information was updated to match the change in NHS-IL12 dosing schedule.
9) Section 7.3.2 Safety Monitoring Committee (SMC): This section has been removed as they study will no longer require monitoring by the CCR SMC as a new DSMB will take over safety monitoring.
10) Section 7.3.2 Data Safety Monitoring Board (DSMB): A data safety monitoring board has been added to the study.
11) Section 12.4.1.1 Study Drug Risks: A statement was added to match the new information provided in the consent to inform the participants of possible toxicity due to the use of M7824.
12) Section 12.5 Consent Process and Documentation: The consent process was updated to be consistent with current IRBO/OHSRP and CCR policies, procedures, and practices.
13) Section 12.5.1 Consent Process for Participants Who Decline Other Treatment Options: To ensure that subjects who decline other possible beneficial therapies received adequate informed consent, consent will be obtained in the presence of an independent consent monitor.
14) Section 14.2.2.1 HLH: This section was added at the request of the NCI SMC to expand the risks to specifically include Hemophagocytic lymphohistiocytosis (HLH).

Amendment March 07, 2022

Protocol Changes:

1) Header and Title Page: The version date has been updated.
2) Section 2.2.2 Screening activities performed after a consent for screening has been signed: The language was updated to allow for flexibility in collection of assessments performed at outside facilities as it is in Dr. Strauss' other protocols.
3) Section 3.7.3 Lost to Follow-up: Language was updated to include that the certified letter sent to participants will receive IRB approval prior to sending.
4) Section 5.1 Biospecimen Collection: An allowance was made for the substitution of tube types based on availability in order to limit potential deviations.
5) Section 5.1 Biospecimen Collection and Section 5.1.5 Circulating tumor DNA: The location of specimen analysis for Circulating tumor DNA has been changed to LTIB and NIH Core Facility from Dr. Liang Cao's Lab.
6) Section 6.1 Data Collection: The section was updated to reference CCR policies and procedures regarding eCRFs/data capture.
7) Section 8.8 Sponsor Protocol Deviation Reporting and Section 9 Clinical Monitoring: These sections were updated for correctness due to the transition of OSRO-monitored studies and implementation of the new electronic CCR PDTS.

Amendment May 12, 2023

Protocol Changes:
1) Header and Title Page: The version date has been updated.
2) Title Page:
a. The PI name, branch information, and contract information have been updated.
b. Per agreement with the Investigational Pharmacy, removed title for the "Investigational Agents" table.
3) Section 7.3 NCI Clinical Director Reporting: This section was updated to remove a specific name for the NIH eIRB system and update CCR reporting contact/designees for deaths not meeting IRB problem/expedited reporting requirements.
4) Section 7.3.2 Data Safety Monitoring Board (DSMB) (OSRO): Added 'OSRO' to the heading to clarify who the DSMB administration falls under.
5) Section 8.6 Reporting Pregnancy: Revised pregnancy reporting text to remove the listing of possible pregnancy outcomes as not required per correspondence with IRBO; intent to follow/capture outcomes was retained with an added option to enroll on a registry.
