# Supplementary material for: Novel Combination Immunotherapy and Clinical Activity in Patients With HPV-Associated Cancers: A Nonrandomized Clinical Trial
Source: JAMA Oncol. 2025 Feb 20;11(4):394–9. doi: 10.1001/jamaoncol.2024.6998 (PMC11843463; doi:10.1001/jamaoncol.2024.6998)
Supplement: Supplement 2. — eAppendix 1. Rationale for the Population, Combination, Dose and Schedule eMethods. eAppendix 2. Supplementary Results eAppendix 3. Supplementary Discussion eTable 1. Participant Characteristics eTable 2. Grade 3, Grade 4 and Serious TRAEs in the Study eTable 3. Grade 3 and 4 TRAEs by Dosing of PDS01ADC and Bintrafusp Alfa eTable 4. Overview of Objective Response Rates and Median Overall Survival Across the Patient Groups and Historic Comparator eFigure 1. Changes in Target Lesions During Treatment With Combination Immunotherapy in Patients With HPV-16–Positive Disease eFigure 2. Overall Survival (OS) by Immune Checkpoint Inhibitor (ICB) Naive or Resistant Disease Status eFigure 3. Schematic of Treatment and Sampling Schedule eFigure 4. HPV-16 Specific T Cell Responses at Baseline and During Combination Therapy in Patients With HPV-16–Positive Tumors eFigure 5. Overall Survival (OS) in the HPV-16–Positive ICB-Resistant Patient Subgroup by PDS01ADC and BA Dose eReferences. [file jamaoncol-e246998-s002.pdf]

# Supplemental Online Content

Floudas CS, Goswami M, Donahue RN, et al. Novel combination immunotherapy and clinical activity in patients with HPV-associated cancers: a nonrandomized clinical trial. *JAMA Oncol.* Published online February 20, 2025. doi:10.1001/jamaoncol.2024.6998

**eAppendix 1.** Rationale for the Population, Combination, Dose and Schedule

**eMethods.**

**eAppendix 2.** Supplementary Results

**eAppendix 3.** Supplementary Discussion

**eTable 1.** Participant Characteristics

**eTable 2.** Grade 3, Grade 4 and Serious TRAEs in the Study

**eTable 3.** Grade 3 and 4 TRAEs by Dosing of PDS01ADC and Bintrafusp Alfa

**eTable 4.** Overview of Objective Response Rates and Median Overall Survival Across the Patient Groups and Historic Comparator

**eFigure 1.** Changes in Target Lesions During Treatment With Combination Immunotherapy in Patients With HPV16-Positive Disease

**eFigure 2.** Overall Survival (OS) by Immune Checkpoint Inhibitor (ICB) Naïve or Resistant Disease Status

**eFigure 3.** Schematic of Treatment and Sampling Schedule

**eFigure 4.** HPV16 Specific T Cell Responses at Baseline and During Combination Therapy in Patients With HPV16-Positive Tumors

**eFigure 5.** Overall Survival (OS) in the HPV16-Positive ICB-Resistant Patient Subgroup by PDS01ADC and BA Dose

**eReferences.**

This supplemental material has been provided by the authors to give readers additional information about their work.

© 2025 Floudas CS et al. *JAMA Oncology*.

**eAppendix 1.** Rationale for the population, combination, dose and schedule

Our group conducted a Phase 1 trial of BA (NCT02517398) in advanced solid tumors, involving dose escalation (1, 3, 10, or 20 mg/kg once every 2 weeks) and dose expansion cohorts. Results reported a manageable safety profile, and the MTD was not reached at the highest dose level in this study, 20 mg/kg. Evidence of clinical activity with M7824 was observed across all evaluated dose levels. Patients with HACs who developed an HPV-specific immune response on M7824 had improved clinical responses. Dose levels of 3 mg/kg, 10 mg/kg, 500 mg, and 1,200 mg Q2W led to exposures in the active range and 1200 mg was chosen as RP2D for further investigation in the expansion cohorts, which included a cohort of patients with HACs. Preliminary results showed BA to have a manageable safety profile and encouraging clinical efficacy and a Phase II trial (NCT03427411) of BA 1200 mg in R/M HACs was conducted. Results from the Phase I dose expansion HAC cohort and the Phase II were reported jointly, with ORR of 30.5% (95% CI, 19.2 to 43.9) in the immune checkpoint inhibitor-naive population. An additional immune checkpoint inhibitor-resistant population was enrolled in the Phase II study, with an ORR of 10% (95% CI, 1.2% to 31.7%). [1-3]

A phase I trial of PDS01ADC (NCT01417546) was also conducted by our group, evaluating escalating 9 dose levels (0.1 mg/kg , 0.5 mg/kg, 1.0 mg/kg, 2.0 mg/kg, 4.0 mg/kg, 8.0 mg/kg, 12.0 mg/kg, 16.8 mg/kg, and 21.8 mg/kg) administered every 4 weeks, which demonstrated that PDS01ADC had an acceptable safety profile with low grade, self-limited treatment-related adverse events (TRAEs) including lymphopenia, flu-like symptoms and low-grade liver transaminases elevation and defined a maximum tolerated dose of NHS-IL12 as 16.8 μg/kg SC every 4 weeks. PDS01ADC resulted in cytokine response peaking 36 hours post-administration and returning close to baseline between day 8 and day 15 post-treatment. Two high-exposure cohorts were added, testing 12 μg/kg SC or 16.8μg/kg SC q2w every 2 weeks, and shown to be well tolerated and associated with biologic activity. [4,5] PDS01ADC 16.8 μg/kg every 4 weeks was well tolerated when combined with the PD-L1 blocking agent avelumab in a Phase 1b trial (NCT02994953). [6]

A Phase I/IIA clinical trial (NCT02065973) of PDS0101 in patients with high-risk HPV infection and biopsy-proven cervical intraepithelial neoplasia (CIN1) studied 3 escalating dose levels based on the R-DOTAP content (1 mg, 3 mg, 10 mg). Participants received 3 doses of vaccine subcutaneously, 21 days apart. PDS0101 was well tolerated, with most AEs being injection site reactions, of mild or moderate severity, commonly resolving in the same day or within a few days. Injection site reactions were more severe and of longer duration in participants receiving DL3. HPV-specific T-cell responses were elicited in 83% (10/12) participants, at all dose levels, regardless of participant HLA type, in both HPV-16+ and HPV-16- participants. Regression of CIN was noted in 60% (6/10) of participants, including CIN related to non-HPV16 high-risk types, such as HPV-18. PDS0101 3 mg was chosen for further evaluation in Phase II trials. [7]

Considering the above, the doses and administration schedule for the agents in the trial regimen were initially: BA 1200 mg every 2 weeks, PDSADC01 16.8 mcg/kg every 4 weeks, and PDS0101 3 mg (per R-DOTAP content) every 4 weeks for 6 doses then every 3 months for 2 doses. Interruptions (any drug) and dose reductions of PDSADC01 to 12 or 8 mcg/kg every 4 weeks were allowed for AE management. This regimen was later modified further as described in the manuscript and the eMethods of the Supplemental material.

© 2025 Floudas CS et al. *JAMA Oncology*.

**eMethods**

*Dosing and schedule*

The initial regimen consisted of 1200 mg of intravenous BA every 2 weeks; 16.8 mcg/kg of subcutaneous PDS01ADC every 4 weeks; and 1 mL (3 mg R-DOTAP, 2.7 mg total peptide) of PDS0101 subcutaneously (split in two 0.5 mL injections) every 4 weeks for 6 doses, then every 12 weeks for 2 additional doses. Interruptions (any drug) and dose reductions of PDS01ADC (to 12 or 8 mcg/kg every 4 weeks) were allowed for AE management.

*Subsequent modifications* allowed BA dose reductions to 600 mg or 300 mg (including starting dose); PDS0101 discontinuation in patients with non-HPV16 tumors; PDS01ADC starting dose of 16.8 mcg/kg for up to a total of 4 consecutive doses, then 8 mcg/kg every 4 weeks, with dose interruptions and reductions to 8, 6, or 4 mcg/kg. A further modification implemented for patients with cervical cancer and prior pelvic irradiation and brachytherapy due to a potential higher risk of grade 3 hematuria was to reduces the starting doses of BA to 600 mg and of PDSADC01 4 mcg/kg, with potential further reduction of BA to 300 mg depending on the occurrence of grade 3 hematuria.

Dose interruption and discontinuation was allowed for the management of treatment-related adverse events (TRAEs).

*Discontinuation* of individual agents was mandated in the case of AEs attributable to an agent of grade ≥4 except: laboratory test result abnormalities either asymptomatic or resolving to grade ≤1 or baseline within 7 days; or of grade 3 except: flu-like symptoms (controllable with standard medical management), tumor flare symptoms, anemia (<8.0 g/dL) manageable with medical management (blood transfusion or erythroid growth factor), laboratory test result abnormalities either asymptomatic or resolving to grade ≤1 or baseline within 14 days, cutaneous keratoacanthoma and squamous cell carcinoma, endocrinopathies manageable with hormone replacement, any other AE medically manageable with minimal risk to the participant and resolving to grade ≤1 or baseline within 14 days.

*Dose interruption* of individual agents was mandated for any grade 2 or 3 AE possibly attributed to that agent until resolution to grade ≤1 unless the AE was not clinically relevant or could be medically managed with minimal risk to the participant. PDSADC01 could be restarted at any scheduled visit. Discontinuation of an agent should be considered for persistence of a related clinically relevant grade 2 or 3 AE >4 weeks.

*Stopping rules*

Suspension of accrual and assessment of safety information will occur in case of permanent discontinuation of ≥1 study drugs due to grade 3 or 4 toxicity in > 1/3 of patients, or any treatment-related death.

*Procedures*

History and physical examination, complete blood cell counts, and serum laboratory tests were conducted at baseline and before each treatment. Radiographic evaluation was performed at baseline and every 8 weeks thereafter. AEs were monitored from initiation of study treatment until 28 days after removal from study treatment or until off-study and graded using NCI Common Terminology Criteria for Adverse Events version 5.0.

HPV genotype was determined by PCR (Onclarity™, Becton Dickinson, Franklin Lakes, NJ or MolecularMD, Portland, OR) on archival tumor tissue if previous testing was unavailable.

Peripheral blood was collected at baseline and on treatment (eFigure 3 in Supplement 2) and assessed for HPV16-specific T cells.

*Peripheral HPV-specific T-cell responses*

Peripheral blood mononuclear cells (PBMCs) were isolated from blood collected in sodium heparin tubes by Ficoll-Hypaque density gradient separation, cryopreserved in 90% heat-inactivated human AB serum with 10% dimethyl sulfoxide, and stored in liquid nitrogen. HPV16-specific T cells were quantified in patients at baseline and during treatment as previously described.[8] Cryopreserved PBMCs were thawed and stimulated *in vitro* with overlapping 15-mer peptide pools encoding HPV16 E6 and E7 oncoproteins. Peptide pools encoding for human leukocyte antigen (HLA) and CEFT (mix of peptides encoding cytomegalovirus, Epstein-Barr virus, influenza, and tetanus toxins) served as negative and positive controls, respectively, and were run in parallel with the HPV16 peptide pools. PBMCs received 7 days of peptide stimulation, with 10 ng/mL IL-7 and IL-15 cytokine support provided on days 3 and 5, followed by a 4-day rest and an overnight restimulation prior to staining. T-cell expression of the degranulation marker CD107a and/or the intracellular cytokines IFN-γ, TNF-α, and IL-2 assessed by flow cytometry was used as the readout of antigen specificity. Calculations of absolute number of HPV16-specific CD4+ and CD8+ T cells were performed as previously described.[8] HPV16-specific T cells were defined as the absolute number of CD4+ or CD8+ T cells positive for the degranulation marker CD107a, or the cytokines IFN-γ, TNF-α, or IL-2, after expansion per 1×106

© 2025 Floudas CS et al. *JAMA Oncology*.

PBMCs plated at the start of the *in vitro* stimulation assay. This method allows for enumeration of the total number of T cells expanding in response to HPV16 peptides spanning the entirety of E6 and E7 proteins. A patient was considered positive for pre-existing HPV16-specific T cells if they had ≥250 CD4+ or CD8+ T cells positive for CD107a, IFN-γ, TNF-α, or IL-2 per $1\times10^6$ cells, as well as a ≥2-fold increase over the HLA– control at baseline. Development of an antigen-specific T-cell response was defined as ≥250 CD4+ or CD8+ T cells positive for CD107a, IFN-γ, TNF-α, or IL-2 per $1\times10^6$ cells and a >2-fold increase in the number of positive cells at a given timepoint during treatment compared to baseline. Multifunctional HPV16-specific responses were defined as CD4+ or CD8+ T cells expressing ≥2 of the above markers and calculated in the same manner.

*Statistical Methods*

The trial was planned to enroll up to 20 evaluable ICB-naïve patients (primary endpoint) following a Simon optimal 2-stage design to rule out a 30% ORR and achieve a result consistent with 60% ORR, with 90% power to reject the null hypothesis and type 1 error (alpha) of 0.1. Assessment of ORR in ICB-resistant patients was exploratory, and no specific sample size was planned for this population. Safety was assessed in all patients who received at least one dose of any study drug. Response was assessed in all patients who received at least one dose of any drug and had disease re-evaluation or exhibited objective disease progression. Descriptive statistics were used to summarize study results and 95% CIs of ORR were calculated using the Clopper-Pearson method. OS and PFS were estimated using the Kaplan-Meier method and differences between survival curves were determined by a log-rank test. Fisher's exact tests were used to compare binary measures across 2 groups. All statistical tests were 2-tailed, and reported p-values were not corrected for multiple testing in this hypothesis-generating study. Statistical analyses were performed using R v4.3.3 (R Core Team, 2022), GraphPad Prism v10.4.0 (La Jolla, CA), and SAS v9.4 (Cary, NC).

*Serious Adverse Events*

An adverse event or suspected adverse reaction is considered serious if in the view of the investigator or the sponsor, it results in any of the following: death; a life-threatening adverse event; inpatient hospitalization or prolongation of existing hospitalization (except pre-planned hospitalizations or planned hospitalizations for pre-existing conditions or procedures required by the protocol without serious deterioration in health); persistent or significant incapacity or substantial disruption of the ability to conduct normal life functions; congenital anomaly/birth defects; important medical events that may not result in death, be life-threatening, or require hospitalization but which based upon appropriate medical judgment, may jeopardize the patient or subject and may require medical or surgical intervention to prevent one of the outcomes listed in this definition. Life-threatening adverse events are those that in the view of either the investigator or sponsor, are placing the participant at immediate risk of death, and do not include an adverse event or suspected adverse reaction that, had it occurred in a more severe form, might have caused death (21CFR312.32).

*Adverse Events of Special Interest* were included in the protocol with a subsequent modification and were defined as mucosal bleeding AEs possibly mediate by TGF-β inhibition.

© 2025 Floudas CS et al. *JAMA Oncology*.

**eAppendix 2.** Supplementary Results

*Safety*

Grade 3 and 4 TRAEs (eTable 3) occurred in 14/21 patients (67%) who received full doses of both PDS01ADC and BA, with fewer occurrences in lower-dose combinations: 7/16 patients (44%) who had reduced PDS01ADC and full BA, 2/7 patients (29%) who had full PDS01ADC and reduced BA, and 3/6 patients (50%) who had both reduced PDS01ADC and BA (see eTable 3).

Treatment-related grade 3 (no higher grade) bleeding events occurred in 9 patients (18%): specifically in 5 patients with cervical cancer (of 14 patients [36%] with cervical cancer), 2 patients with oropharyngeal cancer (of 21 [10%]) and 2 patients with anal cancer (of 10 [20%]). Individual AEs were hematuria (5 patients [10%]), vaginal bleeding (1 patient [2%]), anal bleeding (1 patient [2%]), gastric bleeding (1 patient [2%]), duodenal bleeding (1 patient [2%]), mucosal bleeding not otherwise specified (2 patients [4%]).

*Clinical activity*

In the HPV16+ ICB-resistant subgroup, ORR was 20.7% (6/29; 95% CI, 8.0–39.7%), mOS was 17.0 months (95% CI, 10.4–22.8 months) (eFig. 2B) and mPFS was 2.4 months (95% CI, 1.8–5.0 months). In this group, 8 of 29 patients (27.5%) received full doses of PDS01ADC and BA with responses in 5 of 8 vs. 1 response in 21 patients who received lower doses of PDS01ADC, BA, or both, but with comparable survival observed among these groups (eFig. 3). In the non-HPV16 disease subgroup there was no objective response by RECIST; one patient had CR by iRECIST with full doses of PDS01ADC and BA.

*HPV-specific peripheral T cell responses*

T-cell reactivity against HPV16 E6 and E7 oncoproteins was evaluated at baseline in 23 patients with HPV16+ disease and during treatment (on days 15, 43 and/or 57) in 20 of these patients with available PBMC. HPV16-specific T cells were defined as the absolute number of CD4+ or CD8+ T cells positive for the degranulation marker CD107a, or the cytokines IFN-γ, TNF-α, or IL-2, after expansion per 1×106 PBMCs plated at the start of the in vitro stimulation assay. This method allows for enumeration of the total number of T cells expanding in response to HPV16 peptides spanning the entirety of E6 and E7 proteins. T cells expressing at least 2 of the above markers were considered multifunctional T cells. Most evaluable patients with HPV16+ disease (20/23; 87%), regardless of best overall response (BOR), had detectable HPV16 specific T cells at baseline; similar numbers of patients also had detectable multifunctional HPV16 T-cell responses at baseline (eFigure 4A). However, patients with a BOR of CR or PR or stable disease (SD) were more likely to have developed increased multifunctional T-cell responses at any timepoint post-therapy versus baseline (11/13; 85%) compared to patients with progressive disease (PD) (2/7; 29%) ($p = 0.022$) (eFigure 4B). Representative examples of multifunctional HPV16-specific T cells in 3 patients are shown in eFigure 4C. Notably, of 9 patients with HPV16– disease that were tested, 7 (78%) developed T-cell responses against HPV16 following treatment; only one patient in this group had clinical antitumor activity.

© 2025 Floudas CS et al. *JAMA Oncology*.

**eAppendix 3. Supplementary Discussion**

Although the toxicity profile was manageable, efforts were made to reduce AEs by evaluating reduced starting doses of PDS01ADC and BA as part of the triple combination. For patients receiving reduced doses of PDS01ADC, BA, or both, 7/16 (43.8%), 2/7 (28.6%) and 3/6 (50.0%) had grade 3 and/or 4 toxicities, respectively, compared to patients receiving full doses of PDS01ADC and BA, where 14/21 patients (66.7%) had grade 3 and/or 4 toxicities. While the triple combination with full doses of PDS01ADC and BA did result in added toxicity, response rates appeared to be higher for patients with ICB-resistant HPV16+ disease receiving full doses of both PDS01ADC and BA (5/8; 62.5%), compared to those receiving lower doses of either PDS01ADC, BA, or both (1/12; 8.3%). Notably, in ICB-resistant HPV16+ patients the OS was comparable in patients receiving full versus lower doses of PDS01ADC or BA (eFig. 5). This may be due to overall long-term disease control or impact on tumor growth rates, or due to patient selection, and caution is warranted when interpreting this data. Another explanation is the combination, even with lower doses of PDS01ADC or BA, may have primed patients' tumors to be more sensitive to the next-line of therapy following the study treatment. These survival data are promising and merit further study in larger trials.

© 2025 Floudas CS et al. *JAMA Oncology*.

**eTable 1. Participant characteristics.**

| Characteristic | N | % |
|---|---|---|
| Sex | | |
| Male | 26 | 52% |
| Female | 24 | 48% |
| Tumor type | | |
| Oropharyngeal | 21 | 42% |
| Cervical | 14 | 28% |
| Anal/rectal | 10 | 20% |
| Vulvar/vaginal | 3 | 6% |
| Nasopharyngeal | 1 | 2% |
| Penile | 1 | 2% |
| Number of prior lines of systemic therapies | | |
| 1 | 5 | 10% |
| 2 | 22 | 44% |
| 3 or more | 23 | 46% |
| Prior radiotherapy | 45 | 90% |
| Prior PD-(L)1 blockade therapy | 36 | 72% |
| HPV status | | |
| HPV-16+ | 37 | 74% |
| HPV type other than HPV-16 | 11 | 22% |
| HPV-negative | 1 | 2% |
| Unknown | 1 | 2% |

© 2025 Floudas CS et al. *JAMA Oncology*.

## eTable 2. Grade 3, Grade 4 and Serious TRAEs in the study (N=50)

| | Patients N (%) | |
|---|---|---|
| **Adverse event (AE)** | | **Attribution[a]** |
| *Patients with any G3 TRAE* | 26 (52) | |
| Anemia | 13 (26) | BA |
| Hematuria | 5 (10) | BA |
| Lymphocyte count decreased | 4 (8) | PDSADC01 |
| Alanine aminotransferase increased | 3 (6) | PDSADC01; BA, PDSADC01 |
| Aspartate aminotransferase increased | 3 (6) | PDSADC01; BA, PDSADC01 |
| Mucosal bleeding | 2 (4) | BA |
| Flu like symptoms | 1 (2) | PDSADC01 |
| Hyponatremia | 1 (2) | PDSADC01 |
| CPK increased | 1 (2) | PDSADC01 |
| Mucositis oral | 1 (2) | PDSADC01 |
| Urinary tract obstruction | 1 (2) | BA |
| Vaginal bleeding | 1 (2) | BA |
| Neutrophil count decreased | 1 (2) | PDSADC01 |
| White blood cell decreased | 1 (2) | PDSADC01 |
| Immune-related myocarditis | 1 (2) | BA |
| Cardiac troponin increased | 1 (2) | BA |
| Vomiting | 1 (2) | PDSADC01 |
| Hemophagocytic Lymphohistiocytosis | 1 (2) | BA, PDSADC01 |
| Alkaline phosphatase increased | 1 (2) | BA |
| Anal bleeding | 1 (2) | BA |
| Gastric bleeding | 1 (2) | BA |
| Duodenal bleeding | 1 (2) | BA |
| *Patients with any G4 TRAE* | 2 (4) | |
| Neutrophil count decreased | 1 (2) | PDSADC01 |
| Aspartate aminotransferase increased | 1 (2) | BA, PDSADC01 |
| Alanine aminotransferase increased | 1 (2) | BA, PDSADC01 |
| *Patients with Serious TRAEs* | 13 (26) | |
| Hematuria | 4 (8) | BA |
| Aspartate aminotransferase increased | 2 (4) | PDSADC01; BA, PDSADC01 |
| Alanine aminotransferase increased | 2 (4) | PDSADC01; BA, PDSADC01 |
| Mucosal bleeding | 2 (4) | BA |
| Alkaline phosphatase increased | 1 (2) | PDSADC01 |
| Flu like symptoms | 1 (2) | PDSADC01 |
| Hyponatremia | 1 (2) | PDSADC01 |
| Urinary tract obstruction | 1 (2) | BA |
| Vaginal bleeding | 1 (2) | BA |
| Immune-related myocarditis | 1 (2) | BA |
| Gastroparesis | 1 (2) | BA |
| Vomiting | 1 (2) | PDSADC01 |
| Hemophagocytic Lymphohistiocytosis | 1 (2) | BA, PDSADC01 |
| Anal bleeding | 1 (2) | BA |
| Anemia | 1 (2) | BA |
| Gastric bleeding | 1 (2) | BA |
| Duodenal bleeding | 1 (2) | BA |
| [a] Attributed to BA, PDSADC01, or both | | |

© 2025 Floudas CS et al. *JAMA Oncology*.

**eTable 3. Grade 3 and 4 TRAEs by dosing of PDS01ADC and bintrafusp alfa.**

| Dosing groups | Number of patients with Grade 3 and 4 TRAEs (%) | Grade 3 and 4 TRAEs (number of patients) |
|---|---|---|
| Full-dose PDS01ADC<br>Full-dose bintrafusp alfa | 14/21 (67%) | Anemia (5)<br>Hematuria (4)<br>Alanine aminotransferase increased (2)<br>Aspartate aminotransferase increased (2)<br>Flu like symptoms (1)<br>Mucosal bleeding (2)<br>Neutrophil count decreased (1)*<br>Lymphocyte count decreased (2)<br>Hyponatremia (1)<br>CPK increased (1)<br>Mucositis oral (1)<br>Urinary tract obstruction (1)<br>Vaginal bleeding (1)<br>White blood cell decreased (1)<br>Immune-related myocarditis (1)<br>Cardiac troponin I increased (1)<br>Vomiting (1)<br>Hemophagocytic Lymphohistiocytosis (1)<br>Alkaline phosphatase increased (1) |
| Reduced-dose PDS01ADC<br>Full-dose bintrafusp alfa | 7/16 (44%) | Anemia (5)<br>Lymphocyte count decreased (2)<br>Anal Bleeding (1)<br>Duodenal bleeding (1) |
| Full-dose PDS01ADC<br>Reduced-dose bintrafusp alfa | 2/7 (29%) | Hematuria (1)<br>Anemia(1) |
| Reduced-dose PDS01ADC<br>Reduced-dose bintrafusp alfa | 3/6 (50.0%) | Anemia (2)<br>Alanine aminotransferase increased (1)*<br>Aspartate aminotransferase increased (1)*<br>Gastric bleeding (1) |
| * Grade 4 TRAE | | |

© 2025 Floudas CS et al. *JAMA Oncology*.

## eTable 4. Overview of Objective Response Rates and median Overall Survival across the patient groups and historic comparator

| | HPV Type | ORR | | Comparator ORR | mOS | Comparator mOS |
|---|---|---|---|---|---|---|
| ICB-naive | Non-HPV16 | 0/6 | 0% | 11-24%[a] | | 7-12 mos |
| | HPV16 | 5/8 | 62.5% | | NR (75% 24 mos) | |
| | Total | 5/14 | 35.7% | | 42.4 mos | |
| ICB-resistant | Non-HPV16 | 0/7 | 0% | | | 3-4 mos |
| | HPV16 | 6/29 | 20.7% | | 17 mos | |
| | Total | 6/36 | 16.7% | | 15.8 mos | |

ICB, Immune checkpoint blockade; ORR, objective response rate; mOS, median overall survival; NR, not reached. Historic comparator results referenced in Discussion section.

© 2025 Floudas CS et al. *JAMA Oncology*.

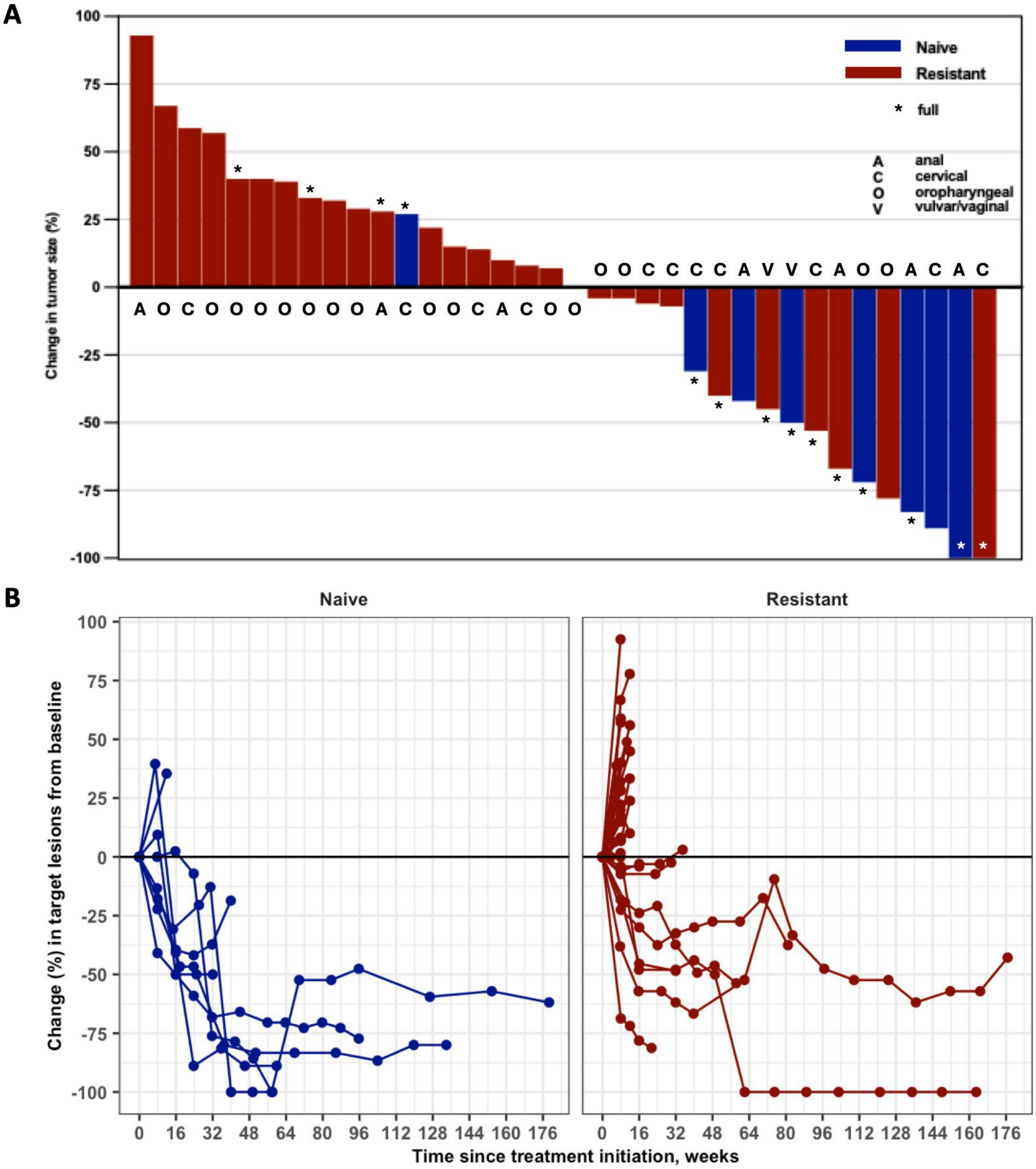


**eFigure 1. Changes in target lesions during treatment with combination immunotherapy in patients with HPV16+ disease.** **(A)** Waterfall plot demonstrating best overall response (BOR) by ICB status, cancer type, and starting dose (full/reduced) of study drugs. **(B)** Spider plots of depth and duration of response by ICB status.

© 2025 Floudas CS et al. *JAMA Oncology*.

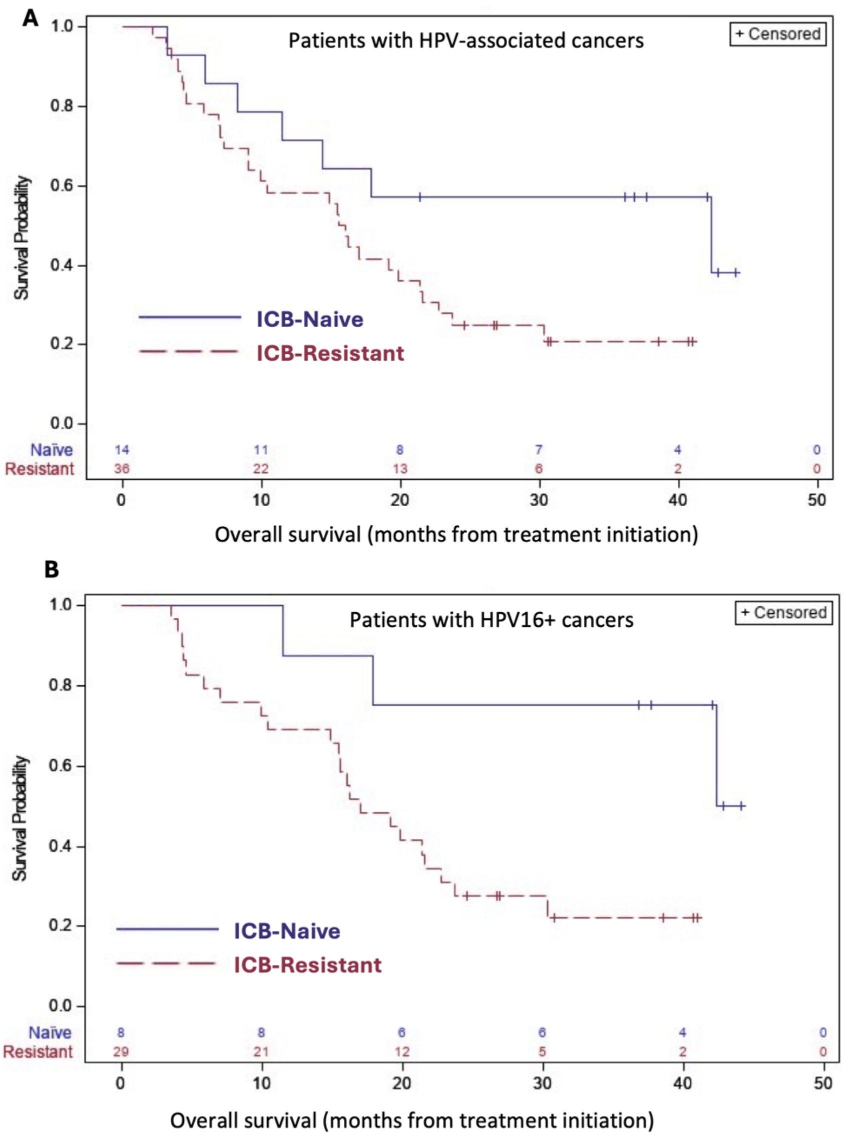


**eFigure 2. Overall Survival (OS) by Immune Checkpoint Inhibitor (ICB) Naïve or Resistant Disease Status. (A)** OS by ICB status in patients with HPV-associated cancers. **(B)** OS by ICB status in patients with HPV16+ tumors. Kaplan-Meier analyses of OS in months from treatment start date. Marks on the curves indicate patients who were censored.

© 2025 Floudas CS et al. *JAMA Oncology*.

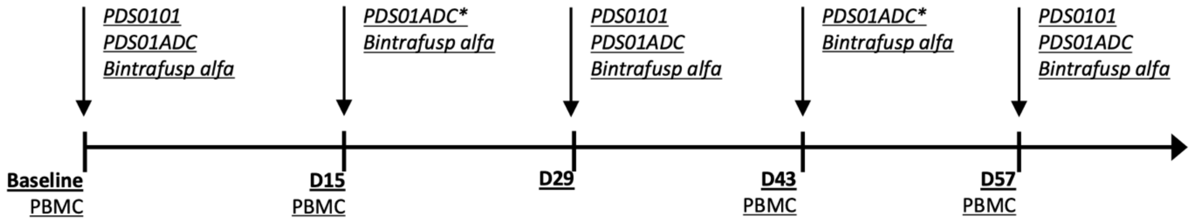


**eFigure 3. Schematic of Treatment and Sampling Schedule.** The therapeutic cancer vaccine PDS0101 was administered every 4 weeks. The immunocytokine PDS01ADC was given at 16.8 mcg/kg every 4 weeks or at 8 mcg/kg every 2 weeks (the additional doses with the q2 week schedule are indicated with asterisks). Bintrafusp alfa was administered every 2 weeks. Research blood was collected immediately prior to treatment initiation, on Day 1, Day 15 (D15), Day 43 (D43), and Day 57 (D57, corresponding to baseline, 2 weeks, 6 weeks, and 8 weeks, respectively. PBMC indicates peripheral blood mononuclear cells.

© 2025 Floudas CS et al. *JAMA Oncology*.

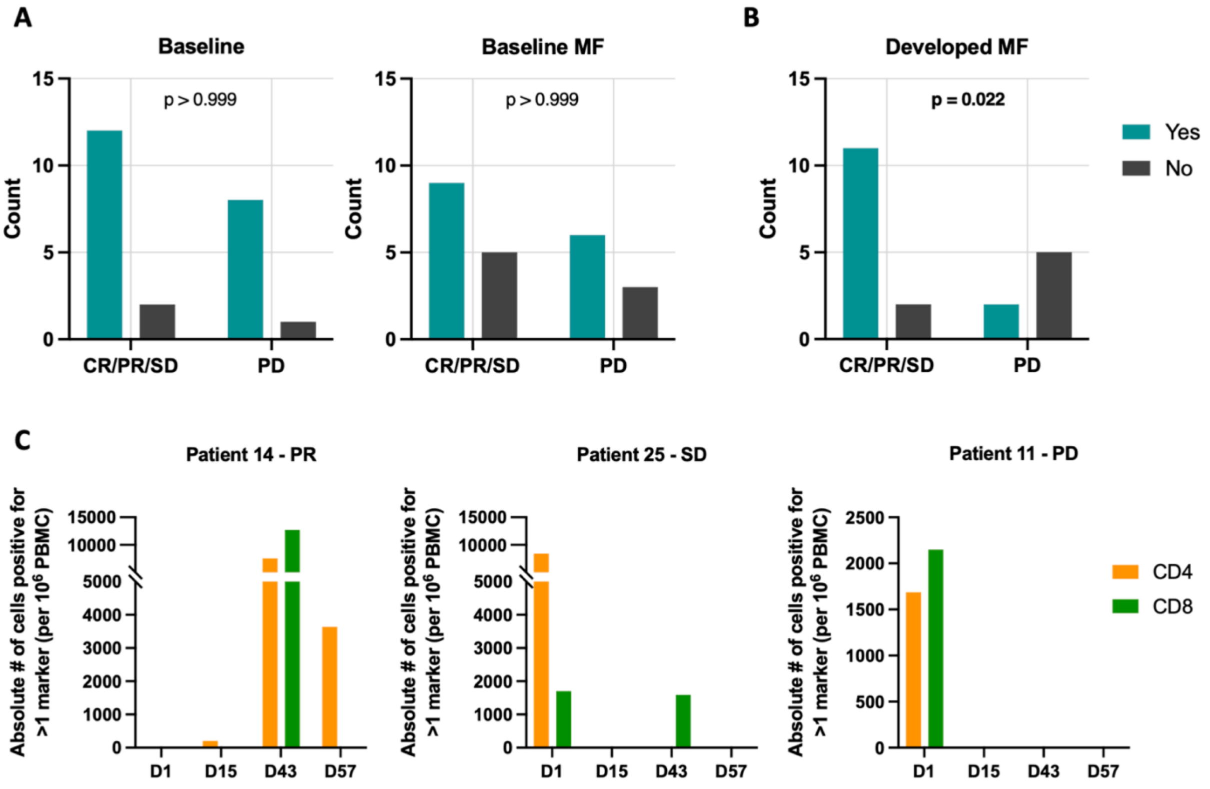


**eFigure 4. HPV16 Specific T Cell Responses at Baseline and During Combination Therapy in Patients With HPV16-Positive Tumors. (A)** Detectable pre-existing T cell responses to HPV16 at baseline, including multifunctional HPV16 T cells, stratified by clinical response. **(B)** Developed multifunctional HPV-16+ T cell responses at any evaluable timepoint post treatment start that are at least 2-fold greater than baseline, stratified by response. **(C)** Representative figures of developed multifunctional CD4+ and CD8+ T cell responses against HPV16 E6/E7 antigens. MF: multifunctional; CR, PR, SD, and PD: complete response, partial response, stable disease, and progressive disease, respectively.

© 2025 Floudas CS et al. *JAMA Oncology*.

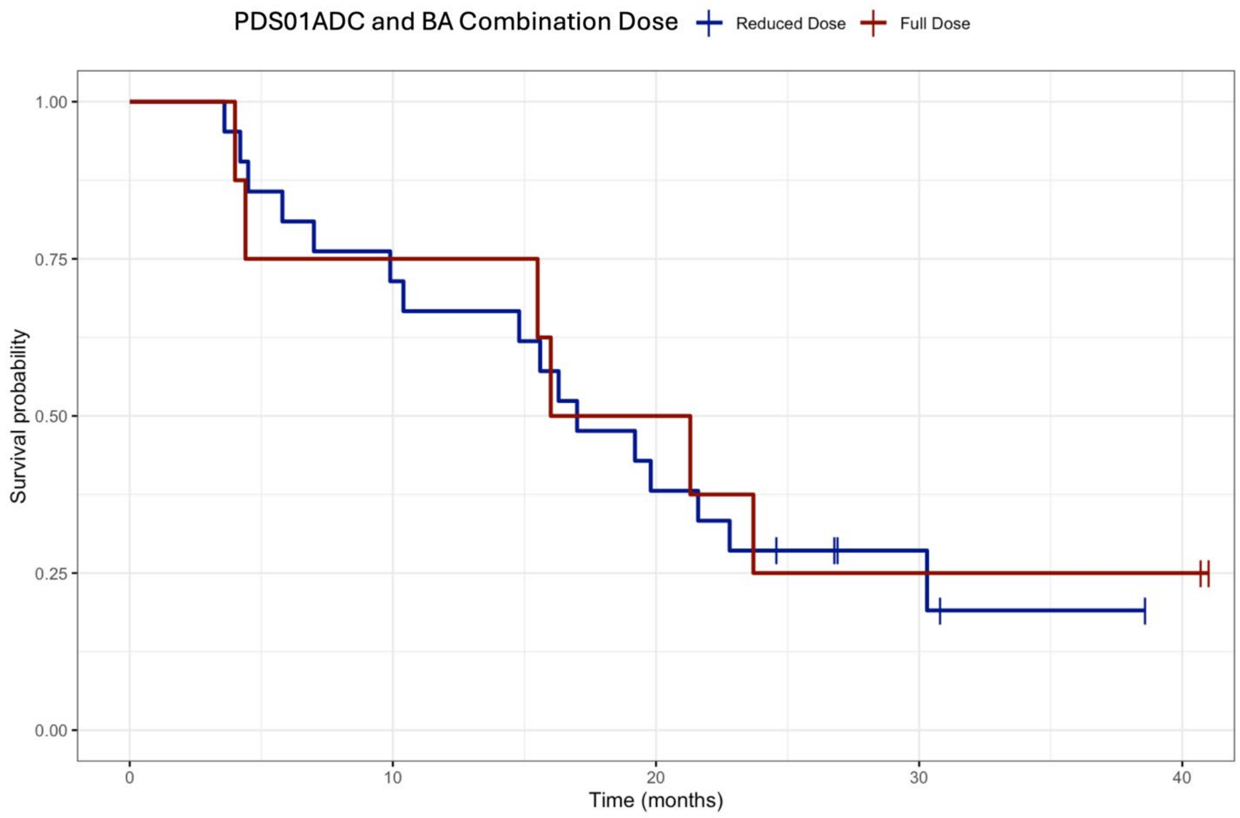


**eFigure 5. Overall Survival (OS) in the HPV16-Positive ICB-Resistant Patient Subgroup by PDS01ADC and BA Dose.** Kaplan-Meier analyses of OS in months from treatment start date by dose of PDS01ADC and BA. Marks on the curve indicate patients who were censored.

© 2025 Floudas CS et al. *JAMA Oncology*.

**eReferences**

1. Strauss J, Gatti-Mays ME, Cho BC, et al. Bintrafusp alfa, a bifunctional fusion protein targeting TGF-β and PD-L1, in patients with human papillomavirus-associated malignancies. *J Immunother Cancer*. Dec 2020;8(2)doi:10.1136/jitc-2020-001395

2. Strauss J, Gatti-Mays ME, Cho B, et al. Abstract CT075: Phase I evaluation of M7824, a bifunctional fusion protein targeting TGF-β and PD-L1, in patients with human papillomavirus (HPV)-associated malignancies. *Cancer Research*. 2019;79(13 Supplement):CT075-CT075. doi:10.1158/1538-7445.Am2019-ct075

3. Strauss J, Heery CR, Schlom J, et al. Phase I Trial of M7824 (MSB0011359C), a Bifunctional Fusion Protein Targeting PD-L1 and TGFbeta, in Advanced Solid Tumors. *Clin Cancer Res*. Mar 15 2018;24(6):1287-1295. doi:10.1158/1078-0432.CCR-17-2653

4. Gatti-Mays ME, Tschernia NP, Strauss J, et al. A Phase I Single-Arm Study of Biweekly NHS-IL12 in Patients With Metastatic Solid Tumors. *Oncologist*. Apr 6 2023;28(4):364-e217. doi:10.1093/oncolo/oyac244

5. Strauss J, Heery CR, Kim JW, et al. First-in-Human Phase I Trial of a Tumor-Targeted Cytokine (NHS-IL12) in Subjects with Metastatic Solid Tumors. *Clin Cancer Res*. Jan 1 2019;25(1):99-109. doi:10.1158/1078-0432.Ccr-18-1512

6. Strauss J, Deville JL, Sznol M, et al. First-in-human phase Ib trial of M9241 (NHS-IL12) plus avelumab in patients with advanced solid tumors, including dose expansion in patients with advanced urothelial carcinoma. *J Immunother Cancer*. May 2023;11(5)doi:10.1136/jitc-2022-005813

7. Wood L, Edwards L, Ferris D, et al. A Novel Enantio-Specific Cationic Lipid R-DOTAP + HPV16 E6 & E7 Antigens Induces Potent Antigen-Specific CD8+ T Cell Responses In-Vivo in Subjects with CIN and High-Risk Human Papillomavirus Infection. SITC 2019 Annual Meeting. 2019:O17.

8. Tsai YT, Strauss J, Toney NJ, et al. Immune correlates of clinical parameters in patients with HPV-associated malignancies treated with bintrafusp alfa. *J Immunother Cancer*. Apr 2022;10(4)doi:10.1136/jitc-2022-004601

© 2025 Floudas CS et al. *JAMA Oncology*.
