# Supplementary material for: Novel Combination Immunotherapy and Clinical Activity in Patients With HPV-Associated Cancers: A Nonrandomized Clinical Trial
Source: JAMA Oncol. 2025 Feb 20;11(4):394–9. doi: 10.1001/jamaoncol.2024.6998 (PMC11843463; doi:10.1001/jamaoncol.2024.6998)
Supplement: Supplement 3. — Data Sharing Statement [file jamaoncol-e246998-s003.pdf]

Data Sharing Statement

Floudas. Novel Combination Immunotherapy and Clinical Activity in Patients With HPV-Associated Cancers. *JAMA Oncol*. Published February 20, 2025.
doi:10.1001/jamaoncol.2024.6998

Data
Additional Information: NCT04287868
Data available: Yes
Data types: Other (please specify)
Additional Information: Deidentified aggregate participant data will be made available for sharing.
How to access data: Deidentified aggregate participant data will be available at an online repository at time of publication.
When available: With publication

Supporting Documents
Document types: None

Additional Information
Who can access the data: Anyone requesting the data.
Types of analyses: For any purpose.
Mechanisms of data availability: Without investigator support.
